# Estimating the environmental impacts of 57,000 food products

**DOI:** 10.1073/pnas.2120584119

**Published:** 2022-08-08

**Authors:** Michael Clark, Marco Springmann, Mike Rayner, Peter Scarborough, Jason Hill, David Tilman, Jennie I. Macdiarmid, Jessica Fanzo, Lauren Bandy, Richard A. Harrington

**Affiliations:** ^a^Nuffield Department of Population Health, University of Oxford, Oxford, OX3 7LFUK;; ^b^Oxford Martin School, University of Oxford, Oxford, OX1 3BDUK;; ^c^Interdisciplinary Centre of Conservation Science, Department of Zoology, University of Oxford, Oxford, OX1 3SZUK;; ^d^Smith School of Enterprise and Environment, University of Oxford, Oxford, OX1 3QYUK;; ^e^NIHR Biomedical Research Centre at Oxford, University of Oxford, Oxford, OX3 7LFUK;; ^f^Department of Bioproducts and Bioengineering, University of Minnesota, St Paul, MN 55108;; ^g^Department of Ecology, Evolution, and Behavior, University of Minnesota, St Paul, MN 55108;; ^h^Bren School of Environmental Science & Management, University of California, Santa Barbara, Santa Barbara, CA 93117;; ^i^The Rowett Institute, University of Aberdeen, Aberdeen, AB25 2ZDUK;; ^j^Nitze School of Advanced International Studies, Johns Hopkins University, Washington, D.C. 20036;; ^k^Global Food Ethics and Policy Program, Johns Hopkins University, Baltimore, MD 21205;; ^l^Nuffield Department of Primary Care, University of Oxford, Oxford, OX2 6GGUK

**Keywords:** food system sustainability, environmental impact of food, ecolabelling

## Abstract

Understanding and communicating the environmental impacts of food products is key to enabling transitions to environmentally sustainable food systems [El Bilali and Allahyari, Inf. Process. Agric. 5, 456–464 (2018)]. While previous analyses compared the impacts of food commodities such as fruits, wheat, and beef [Poore and Nemecek, *Science* 360, 987–992 (2018)], most food products contain numerous ingredients. However, because the amount of each ingredient in a product is often known only by the manufacturer, it has been difficult to assess their environmental impacts. Here, we develop an approach to overcome this limitation. It uses prior knowledge from ingredient lists to infer the composition of each ingredient, and then pairs this with environmental databases [Poore and Nemecek *Science* 360, 987–992 (2018); Gephart et al., Nature 597, 360–365 (2021)] to derive estimates of a food product’s environmental impact across four indicators: greenhouse gas emissions, land use, water stress, and eutrophication potential. Using the approach on 57,000 products in the United Kingdom and Ireland shows food types have low (e.g., sugary beverages, fruits, breads), to intermediate (e.g., many desserts, pastries), to high environmental impacts (e.g., meat, fish, cheese). Incorporating NutriScore reveals more nutritious products are often more environmentally sustainable but there are exceptions to this trend, and foods consumers may view as substitutable can have markedly different impacts. Sensitivity analyses indicate the approach is robust to uncertainty in ingredient composition and in most cases sourcing. This approach provides a step toward enabling consumers, retailers, and policy makers to make informed decisions on the environmental impacts of food products.

## Significance

One barrier to enabling transitions to more environmentally sustainable food systems is the lack of detailed environmental impact information. We provide an initial approach to overcome this barrier using publicly available information to derive first estimates of the environmental impact of >57,000 food products across four indicators: greenhouse gas emissions, land use, water stress, and eutrophication potential. Pairing it with a measure of nutrition shows a tendency for more nutritious foods to be more environmentally sustainable, and that like-for-like substitutes can have highly variable environmental and nutritional impacts. By estimating the environmental impacts of food products in a standardized way, our approach provides a step to enable informed decision making by end users such as consumers and policy makers.

Transitions to environmentally sustainable food systems are urgently needed (1, 2). If diets and food systems continue to transition along recent trajectories, then international climate and biodiversity targets would be missed in the next several decades, even if impacts from other sectors were rapidly reduced or eliminated (3, 4). These same food system transitions would also lead to increased rates of diet-related diseases such as diabetes, heart disease, stroke, and some cancers (1, 5).

One key step to enabling transitions to an environmentally sustainable food system capable of meeting international environmental targets is to estimate and then communicate the environmental impacts of food products available for purchase (6). This information is increasingly desired. Consumers increasingly want to make decisions on the environmental sustainability of foods (7), food corporations are setting ambitious net zero greenhouse gas targets (8, 9), and food retailers are beginning to implement front-of-pack ecolabels on their food products (10). While previous analyses were a step toward providing environmental impact information on foods, they focused on food commodities such as fruits, red meat, or nuts (11). This leaves a major information gap, as the majority of the tens of thousands of food products for purchase at food retail stores contain multiple ingredients. This means the environmental impacts of most food products are not readily known. There are at least two reasons for this: First, the exact amount of each ingredient and their supply chain in each food product are often considered a trade secret, and thus the quantitative composition of a product’s ingredients is not often provided on a food’s ingredient list. Second, the sheer number of food products makes the task daunting, as an individual retailer often markets tens of thousands of food products. Although environmental certification labels such as the Roundtable on Sustainable Palm Oil and the Marine Stewardship Council for seafood are an initial step to communicating the environmental impacts of foods, these certifications cover a small set of foods and do not report a quantitative measure of a food’s environmental impact. This makes it difficult to compare the sustainability of foods labeled with different environmental certifications and foods not labeled with any certification.

To begin addressing this information barrier, we developed and tested the accuracy of an algorithm that uses publicly available information to derive first estimates of the environmental impacts of food products. Using these results, we investigated trends in environmental sustainability across types of food products. We further illustrated two potential applications of this approach, first by examining the correlation between the environmental and nutritional impacts of food products, and second by investigating the variation in environmental and nutritional impacts of similar and potentially substitutable foods.

## Results

### 1.0 Estimating Impacts of Food Products.

The environmental impact of a multi-ingredient food product is determined by the mass and environmental impact of each ingredient. However, full quantitative composition information is publicly available for ∼3% of the 57,185 food products in our dataset, which includes products from 8 food retailers in the United Kingdom and Ireland. This means that the percent composition of some or all ingredients in most products must be estimated before assessing the product’s environmental impact.

**Fig. 1.**
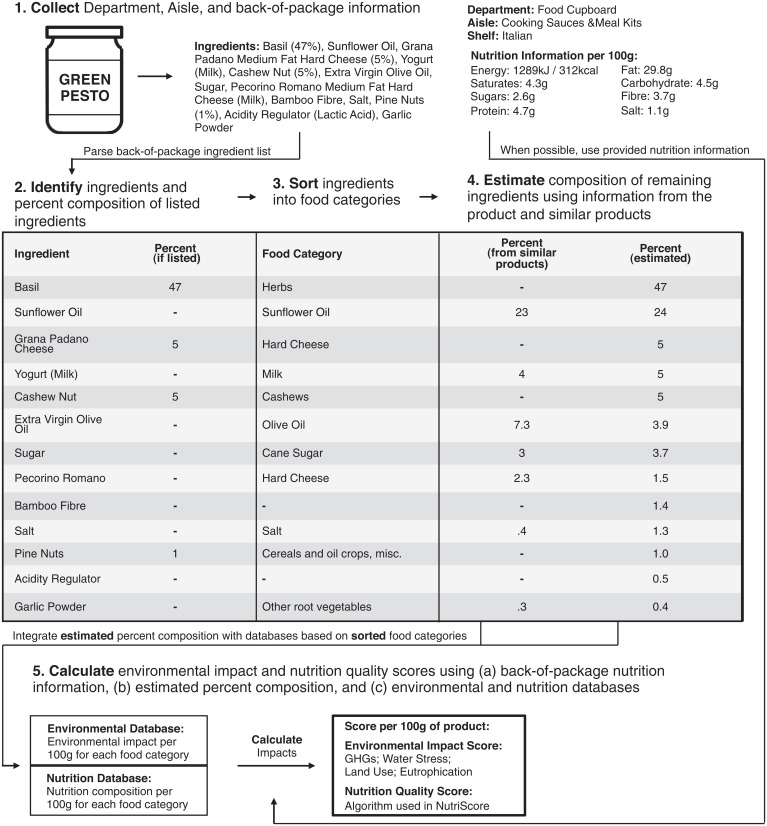
Approach used to estimate the environmental impact score for each food product. See *Methods* and *SI Appendix*, Supplementary Information Text, for more information.

To overcome this information gap, we developed and tested an algorithm that uses publicly available information to derive a first estimate of the environmental impact of food products across multiple environmental indicators (Fig. 1). Using government regulations in the United Kingdom and Ireland that require ingredients to be listed on each product in decreasing order of their abundance and for the percent composition of characterizing ingredients (e.g., the beef in beef lasagna) to be provided on packaging information, we devised an algorithm that uses prior knowledge from similar products (the 10.4% of ingredients that had a percent composition listed in the ingredients list) to infer the composition of ingredients for which such information is not provided (the remaining 89.6% of ingredients; see *SI Appendix*, Table S1). We paired the estimated composition information with environmental databases that quantify “cradle-to-retail” impacts of food production systems to derive environmental impact estimates for food products across four environmental indicators: greenhouse gas emissions, scarcity weighted water use (12), land use, and aquatic eutrophication potential (see *SI Appendix*, Table S2 for the environmental categories). The primary environmental database used is HESTIA (13), which is a growing resource that builds on the work of Poore and Nemecek (14). Because HESTIA does not include capture fish, we supplemented it with data from the Blue Food Assessment (15), assuming a 50:50 split between aquaculture and wild-caught fish as recently estimated (16). We did not identify ingredient sourcing, such as country of origin, as this was not available for most products, and this is needed to fully understand the impacts of different foods. However, we did identify organic ingredients and products when they were labeled. To incorporate how uncertainty in sourcing may affect a product’s environmental impact (e.g., as may result from differences in agricultural production location and method), we derived a first estimate of the mean environmental impact for each indicator and the variance around it using a Monte Carlo analysis. In this analysis, producer-level environmental impact estimates were randomly and repeatedly selected to pair with the composition of each ingredient in each product. The food product information in this analysis was from fooDB, which collects information from the online stores of food retailers on a weekly basis (17). See the *Methods* and *SI Appendix*, Supplementary Information Text, for more detail on how the composition of ingredients was estimated and then used to derive an environmental impact score for each food product.

To visualize the results of our analyses, and because time-restricted consumers may prefer simpler ecolabels (18), we derived a single estimated composite environmental impact score per 100 g of product that ranges from 0 (no impact) to 100 (highest impact). This composite score condensed information from the four environmental indicators, placed equal weight on each indicator, and is on a linear scale (see *SI Appendix*, Figs. S1–S6 for the impacts for each environmental indicator for products in the product classifications used by retailers; see *SI Appendix*, Supplementary Information Text for an example calculation). This means, on average, across the 4 environmental indicators, a product with an estimated environmental impact score of 10 has 5 times the impact of a product with a score of 2, but half the impact of a product with a score of 20. Pairwise correlations between the ordered environmental impacts of each indicator showed that foods with a low environmental impact for one indicator on average have low impacts for other indicators (*P* < 0.05 for all Spearman’s correlations; see also *SI Appendix*, Fig. S7 and Table S3), although there are some exceptions to these trends. For example, almond production results in relatively few greenhouse gas emissions but typically results in high levels of water stress (14), whereas fishery-caught crustaceans can result in high amounts of greenhouse gas emissions but require little to no land use (15). The environmental impact estimates for each indicator remain available for situations that are better suited to the disaggregated estimates—for instance, when companies have targets focusing on a single environmental outcome (e.g., as in net zero greenhouse gas emissions policies).

We report impacts per 100 g of product, but note that the serving size of a given type of product may be more or less than 100 g. Having standardized serving sizes would allow for environmental impacts to be reported in quantities that reflect the amount of food that may be consumed in a single sitting. However, because serving sizes in the United Kingdom and Ireland are not yet standardized, and because manufacturers’ suggested serving sizes for similar products were often variable (e.g., ranging from <15 to >800 g for ready meals), we report impacts per 100 g of product. Correlations between the estimated impacts per reported serving size and per 100 g are shown in *SI Appendix*, Fig. S8.

The estimated environmental impact score and the estimated impact for each indicator are right skewed. The median average estimated environmental impact score is 1.6, the 75^th^ percentile score is 4.1, and the 95^th^ percentile score is 14.1, with a similar skew observed for each environmental indicator (*SI Appendix*, Figs. S1–S5). This indicates that the majority of food products available for purchase at United Kingdom and Ireland food retailers have relatively low environmental impacts compared to the products with the highest impact. This is perhaps unsurprising due to the multitude of processed food products composed of low-impact plant-based commodities (e.g., chips, crisps, biscuits). In this scoring system, many of the highest impact products (e.g., having a score close to 100) were dried beef products such as biltong and beef jerky, which contain more than 100 g of uncooked beef per 100 g of final product, while many of the lowest impact products were composed mainly of water, such as sugary drinks. The estimated environmental impacts account for the processing and transportation of commodities to retail stores, but do not incorporate postproduction processing, packaging, and transportation of, for example, converting sugar into a sugar-sweetened beverage or flour and butter into a croissant. This is unlikely to have a large influence on the estimated environmental impact scores as the large majority of food-related environmental impacts result from agricultural production (14), but it is important to note that this may affect the estimated scores for, for example, air-freighted produce or highly processed foods composed of agricultural commodities with low environmental impacts (19, 20).

### 2.0 Testing the Estimated Environmental Impacts.

We tested the accuracy of the algorithm on all of the products for which the composition of every ingredient was provided in the ingredient list (*n* = 1,547). These products were from seven food retailers and included a diverse array of products such as ready meals, breakfast cereals, fruit drinks, pastries, and sweet and savory snacks.

To test the algorithm, we compared the estimated environmental impact scores derived when using the ingredient composition provided in back-of-package ingredient lists against the scores estimated when composition for the same products was imperfectly known. To do this, we used the known composition of each ingredient as reported on the food product’s ingredient list to first calculate the environmental impact score of these products. We call this the “known environmental impact score” because it was calculated when the percent composition of each ingredient was fully known. We then recalculated the environmental impact using our algorithm for the same products, but when we randomly selected subsets of composition information to be unknown but while retaining the order of ingredients as listed on the product (see *SI Appendix*, Supplementary Information Text, for more information). We call this the “estimated environmental impact score” because it was calculated when the percent composition of some to all ingredients in the product was not known. To test the algorithm’s accuracy, we compared the log ratio of the estimated impact to the known environmental impact score for each product. A log ratio of 0 indicates that the estimated and known impacts were identical, while a ratio >0 indicates that the estimated impact was greater than the known impact.

**Fig. 2.**
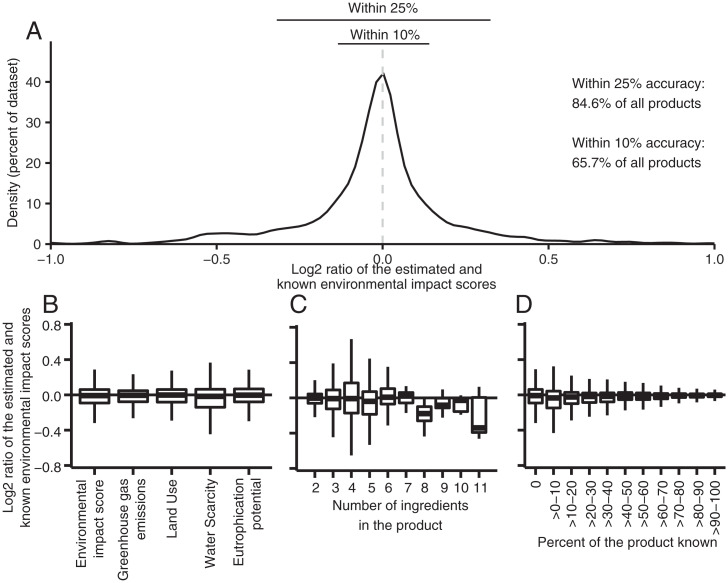
Accuracy of estimated environmental impact scores. Panels show (*A*) the distribution of the accuracy when the percent composition of no ingredients is known, how accuracy varies with (*B*) the number of similar products when the composition of no ingredients is known, (*C*) the number of ingredients in the product when the percent composition of no ingredients is known, and (*D*) the percentage of the product’s ingredient composition is known. In (*A*) the x-axis is cut off at −50% and 50% for visibility; an additional 31 data points outside these limits, or 2.0% of the data sample, are available in *SI Appendix*, Dataset S3. Accuracy is the difference between the known and estimated environmental impact score (in percent), and is calculated as estimated environmental impact score/(known environmental impact score – estimated environmental impact score). The known environmental impact score is calculated using information in the ingredient list, while the estimated environmental impact score is calculated using randomly selected subsets of composition information for each product. Boxplots in *B*–*D* indicate, from bottom to top, the 25th percentile – 1.5*IQR (interquartile range), 25th percentile, median, 75th percentile, and 75th percentile + 1.5*IQR.

The algorithm reasonably estimated a product’s environmental impact (Fig. 2). When we assumed that only the rank order of ingredients in a product was known (as is provided for all food products) and the percent composition of no ingredient was known, the estimated environmental impact score was on average, across all products tested, 1.6% lower (95% CI = 3.3% lower to 0.2% higher) than the known environmental impact. The estimated impacts were not significantly different from the known environmental impact scores (paired *t* test, *P* = 0.9603, degrees of freedom [df] = 3,310), nor were they different for any of the 4 environmental indicators (paired *t* tests, *P* = 0.941 for greenhouse gas emissions, df = 3,311; *P* = 0.872 for land, df = 3,310; *P* = 0.991 for water scarcity, df = 3,310; and *P* = 0.785 for eutrophication potential, df = 3,311). The estimated environmental impact score was within 10% of the known score for 65.7% of products and within 25% for 84.6% of products (Fig. 2*A*). Importantly, the algorithm infrequently mischaracterized the environmental impact of products: 5.3% of the products assessed had an estimated environmental impact less than two-thirds or more than three halves the known impact, while 1.8% had an estimated impact less than half or more than twice the known impact. Across all of the products we used to validate, the average difference between the known and estimated environmental impacts was 0.05 kg CO_2_e and 0.08 m^2^ of land, which increased to 0.17 kg CO_2_e and 0.26 m^2^ for the least accurate estimates (compared to beef, which typically had an impact of >15 kg CO_2_e and >50 m^2^ per 100 g of product). Results were similar when looking at individual environmental indicators instead of a product’s composite environmental impact score (Fig. 2*B*), while the accuracy of the algorithm did not vary with the number of ingredients in products (Fig. 2*C*; linear regression model, *P* value on slope = 0.899, df = 8). In addition, the algorithm’s average accuracy increased, while the variance in the algorithm’s accuracy decreased as more composition information about a product was known (Fig. 2*D*; linear regression model, *P* value on slope < 0.001, df = 1,404). Additional tests using other metrics of accuracy, as well as the effect that ingredient sourcing may have on a product’s environmental impact, further show that the algorithm robustly estimates the ingredient composition and the environmental impact of most food products (see *SI Appendix*, Supplementary Information Text, for a discussion of these tests; see also *SI Appendix*, Figs. S9–S11 and Tables S4 and S5).

The algorithm was reasonably accurate for products categorized into the different food classifications used by retailers (Departments, Aisles, and Shelves). When examining products classified to different Shelves, the algorithm’s average accuracy across Shelves was within 10% of the known score for 70.8% of Shelves (450 of 635) and within 25% of the known score for 87.9% of Shelves (558 of 635), while the 95% confidence intervals around the known and estimated environmental impact scores overlapped for 68.6% of the Shelves (*SI Appendix*, Fig. S12; 436 of 635).

In many instances, the accuracy estimated above is within a worst-case scenario, in which only the rank order of ingredients in the product was known. In contrast, UK labeling regulations require the composition of characterizing ingredients (e.g., beef in a beef lasagna) to be provided. This means that most products (76.2% of multi-ingredient products in our dataset) provided composition information for at least a single ingredient, while 37.8% and 23.8% of products had an ingredient list that identified >50% and 75% of ingredients by mass, respectively. The algorithm’s accuracy increased when we instead assumed that the composition of some ingredients in the product was known (see Fig. 2*D*), which is more indicative of the information currently available on food products in the United Kingdom and Ireland.

### 3.0 Environmental Impact of Food Categories.

We examined the estimated environmental impact of foods categorized into the different food classifications used by food retailers. These food classifications increase in specificity from Department (e.g., “Bakery”), to Aisle (e.g., “Croissants, Brioche, and Pastries”), to Shelf (e.g., “Croissants”). Here, in the main text, we focus on products available at Tesco because it is the largest food retailer in the United Kingdom (21) and because each retailer has its own classification system. To better enable comparison between similar and potentially substitutable foods, we additionally sorted Tesco products into one of eight broad food types based on the product’s Aisle and Shelf: Beverages; Fruits, Vegetables, and Nuts; Cereals and Bread; Snacks; Desserts; Kitchen Accessories; Prepared foods; and Dairy, eggs, meat, and plant-based alternatives. We did not use the classifications for Tesco’s Departments for this comparison because they contain a combination of foods that may not be substitutable. For instance, Tesco’s “Frozen Food” Department has Aisles that contain a combination of fruit, vegetables, pastry, ready meals, and meat.

**Fig. 3.**
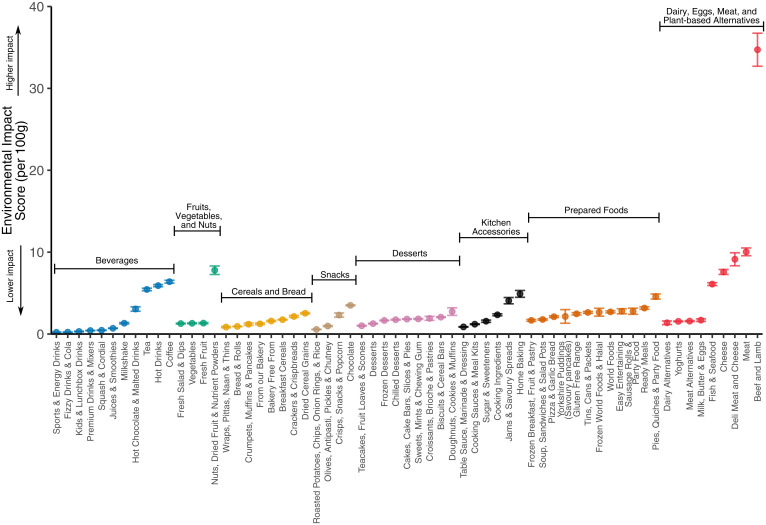
Environmental impact scores per 100 g of products in Tesco Aisles. Points indicates mean impact of all products categorized to the Aisle, and error bars indicate ±1 SEM. Aisles are colored by food type. Food types are shown from lowest median environmental impact on the left to highest median environmental impact at the right. Aisles within food types are ordered from lowest to highest mean environmental impact score. When plotting, Aisles containing similar products were condensed for visibility and clarity (see *SI Appendix*, Supplementary Information Text ). For instance, the Aisles “Fresh Vegetables” and “Frozen Vegetables” were condensed into “Vegetables.”

At Tesco, Aisles with the lowest estimated environmental impacts are often sugary drinks and other beverages composed primarily of water (Fig. 3). This is because these products contain small quantities of sugar and other ingredients (e.g., flavoring, syrups, fruit) per 100 g, with the majority of the product composed of water. Vegetables, snacks (e.g., chips, crisps, popcorn), dairy and meat alternatives, some cereal grains, and breads had an estimated environmental impact score below 2. Many desserts (e.g., cakes, biscuits, pies), other cereals and breads, and prepared foods (e.g., pizzas, ready meals) had an estimated environmental impact score that ranged from 2 to 5. Higher impact Aisles with an average estimated score from 5 to 10 included nuts, sweet and savory spreads, cheese, fish, and some meats (pork and poultry). The highest impact Aisles with estimated scores >10 primarily contained beef and lamb products. Trends at other retailers follow similar patterns (*SI Appendix*, Fig. S6).

Sensitivity analyses show that a lack of ingredient sourcing information is a potential limitation. The estimated median impact of a product (as derived from the Monte Carlo analysis) is on average 87% and 33% higher than the 5th and 25th observed percentile impacts, while the 75th and 95th observed percentile impacts are 72% and 300% higher than the median impact, respectively (*SI Appendix*, Fig. S13). While in most instances the difference between a most sustainably sourced product (5th percentile impact) and least sustainably sourced (95th percentile impact) product is not large, corresponding to a median difference of 0.06 CO_2_e and 0.10 m^2^ of land per 100 g of product, it can in certain cases equate to a difference in greenhouse gas emissions that exceeds 4 kg CO_2_e per 100 g. This implies that while uncertainty in sourcing may not have a large influence on the estimated environmental impact of most products, it does indicate that more transparent ingredient sourcing is needed to derive more accurate environmental impact estimates. See the *SI Appendix*, Supplementary Information Text for additional discussion.

### 4.0 Correlations between Environmental and Nutritional Impacts.

One potential use of the environmental information is to pair it with a measure of nutrition quality to illustrate potential tradeoffs between environment and nutrition. Previous analyses focusing on single-ingredient foods found a general trend for healthy foods to have low environmental impacts and for less healthy foods to have high environmental impacts (11, 22). However, while useful to investigate broad trends, these analyses are limited because most of the products available for purchase in UK food retail stores that provided an ingredient list contained more than one ingredient (96.4% of products in our data sample). It is therefore unclear whether this tendency is also observed across the array of products available in food retail stores.

We assessed the nutrition quality of products using NutriScore, which is a food health profiling method used in multiple countries that has improved population health outcomes (23, 24). NutriScore gives a numeric score to food products for each of seven food components: calories; salt; saturated fats; sugar; protein; fiber; and fruits, nuts, vegetables, and certain oils (olive oil, nut oils, and rapeseed oil). These numeric scores are then summed and converted into an A (most nutritious) to E (least nutritious) ranking, which we converted to a numeric score ranging from 1 (most nutritious) to 5 (least nutritious) to allow for averaging across products (see the *SI Appendix*, Supplementary Information Text for an example calculation). However, NutriScore is known to have limitations. For example, it does not account for how processing or home preparation (e.g., frying and sautéing) may affect the nutrition impact of a food. However, a product’s estimated NutriScore is highly correlated with a product’s estimated nutrition impact under alternative approaches that incorporate these aspects of food preparation (25). Due to this and other limitations, NutriScore is being revised to better reflect evidence from epidemiological and public health literature.

**Fig. 4.**
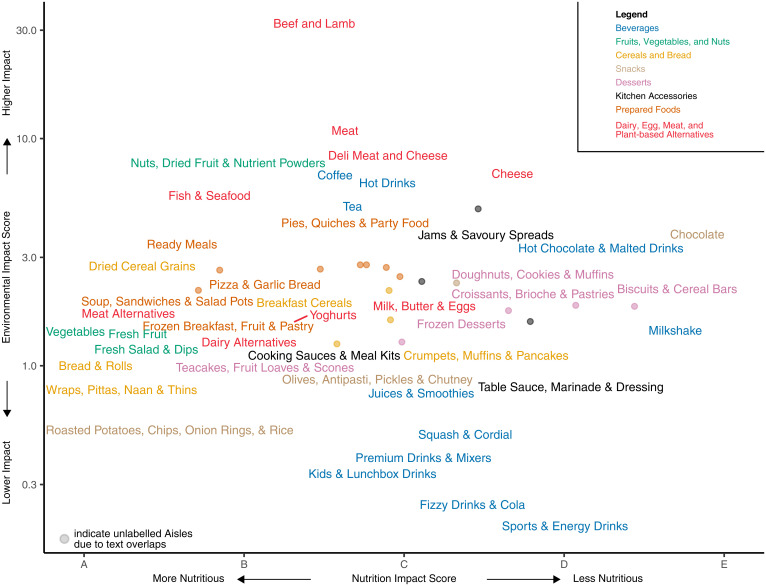
Environmental impact score and nutrition impact score per 100 g of multi-ingredient food products. Data were limited to products available for purchase from Tesco, and were categorized into Aisles using information from Tesco’s website. Colors indicate food types. Points indicate the environmental impact and nutrition impact scores of Aisles not denoted by a text label. When plotting, Aisles containing similar products were condensed for visibility and clarity (see *SI Appendix*, Supplementary Information Text ). For instance, the Aisles “Fresh Vegetables” and “Frozen Vegetables” were condensed into “Vegetables.” Labels were jittered to avoid overlap. See *SI Appendix*, Fig. S10 for the impacts of Aisles within each food type were separated into different panels, and *SI Appendix*, Fig. S11 for data from Tesco in which Aisles were not condensed for visibility and for data from the eight other food retailers in the analysis.

Across all retailers, comparing the mean estimated environmental and nutritional impact of retail Aisles containing only food products suggests a tendency for more environmentally sustainable Aisles to be more nutritious than less sustainable Aisles, but with large variation around this general trend (Spearman’s rho = 0.258, *P* ≤ 0.001). This correlation was also significant for 3 of the 8 retailers in this analysis (Fig. 4 and *SI Appendix*, Figs. S14 and S15; *P* < 0.05 for 3 of 8 retailers; see *SI Appendix*, Table S6 for regression results). Correlations including only drinks were significant across all of the retailers and for one individual retailer, while correlations including both foods and drinks were significant across all of the retailers but for none of the 8 individual retailers (*SI Appendix*, Table S6).

Many Aisles at Tesco were win-wins and were more nutritious and sustainable than most other Aisles (e.g., an estimated environmental and nutritional impact below the median of all of the Aisles examined). These Aisles included, for instance, fruits, vegetables, salad, breakfast cereals, some breads, and meat alternatives (e.g., tofu, vegan sausages). Conversely, there were numerous lose-lose Aisles at Tesco with nutrition and environmental impacts above the median. This includes Aisles such as cheese, chocolate, savory pies, and quiches. Win-lose Aisles (good nutrition composition but above median estimated environmental impact) included fish and seafood, nuts, and some ready meals. Nuts, however, suffer in this comparison from an environmental perspective because they are typically consumed in quantities smaller than 100 g, although nuts are calorie dense and could contribute to weight gain if consumed in excess. Beef and lamb is also a win-lose Aisle when using NutriScore, but evidence suggests that the health and nutrition impacts of beef can range from detrimental to beneficial, depending on the context in which it is consumed: studies in high-income and high-consuming contexts indicate that increasing consumption of red meat would negatively affect health outcomes (26), whereas red meat consumption (and, more broadly, animal-based foods) in food-insecure contexts can be integral to nutrition security (27). Lose-win Aisles (poor nutrition quality but below median environmental impacts) included sweet cakes and pies, sugary drinks (colas, squash, cordials, fruit juices), frozen desserts, and table sauces. Many of these lose-win categories included processed food products that contain ingredients with low environmental impacts but that are also known to contribute to poor health outcomes (e.g., sugar, salt, added fats, refined grain flours).

Sensitivity analyses indicate that the results discussed above are robust. Results from analyses on foods that contain a single ingredient also indicate that more nutritious foods are often more sustainable than less nutritious foods (*SI Appendix*, Fig. S16; Spearman’s rho = 0.381, *P* ≤ 0.001; see also *SI Appendix*, Fig. S17 for environmental impacts of the environmental database food categories). This supports results from recent analyses (11, 22). We also obtained similar results when repeating the analysis when the estimated environmental impact scores were randomly sampled from within the uncertainty identified during the Monte Carlo analysis described above (*SI Appendix*, Table S6 and *SI Appendix*, Supplementary Information Text), as well as when limiting the analysis to only products for which the percent composition of all ingredients was provided in the ingredients list (*SI Appendix*, Table S7).

**Fig. 5.**
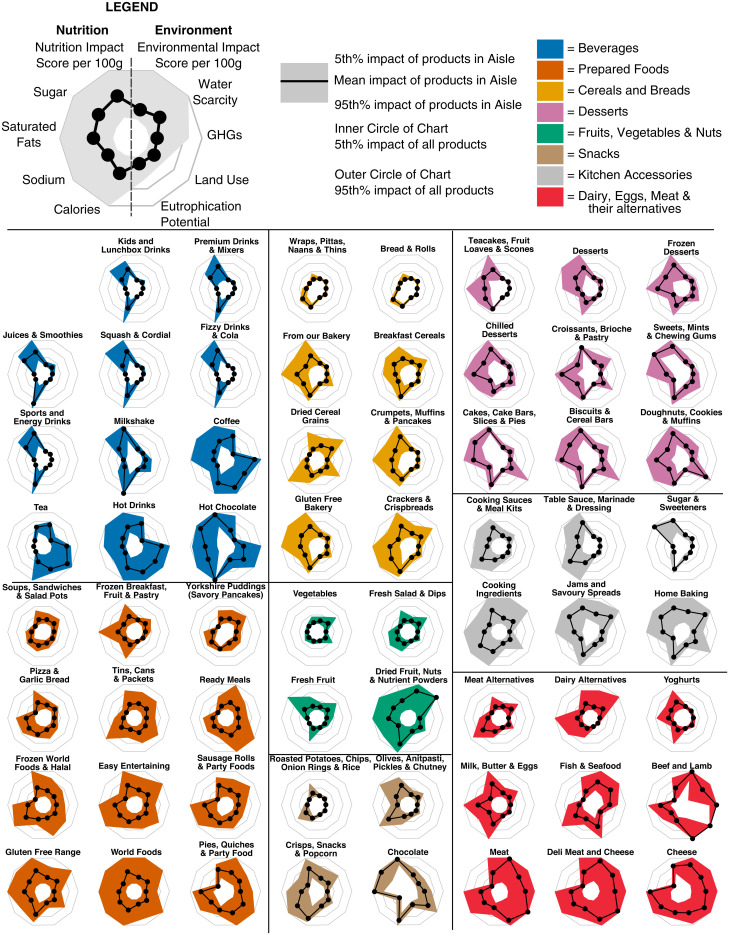
Radar plots indicating the variation in impacts per 100 g of product within each retail Aisle. Solid vertical and horizontal lines separate radar plots into one of eight food types, which are further differentiated by color. Within each food type, radar plots are ordered such that the Aisle with the lowest mean environmental impact score and nutrition impact score are in the upper left, and the Aisle with the highest environmental and nutrition impact are in the bottom right. In each radar plot, the black line indicates the mean impact of products in the Aisle while the shaded area indicates the 5th to 95th percentile impacts of the products within that Aisle. Inner and outer bounds of the radar plot correspond with 5th and 95th percentile impact of all food products available at Tesco, respectively. For the four individual nutrition indicators, the values used for plotting are the number of points assigned to each product for that food component using the NutriScore algorithm. For the four environmental indicators, the values used for plotting are the environmental impact per 100 g of product for the environmental indicator. When plotting, Aisles containing similar products were condensed for visibility and clarity (see *SI Appendix*, Supplementary Information Text). For instance, the Aisles “Fresh Vegetables” and “Frozen Vegetables” were condensed into “Vegetables.”

The estimated environmental and nutritional impacts across products within broad types of foods (e.g., beverages, snacks, prepared foods, plant-based alternatives) and within a given Tesco Aisle were variable (Fig. 5). For example, when looking at prepared foods, the Aisles “Soups, Sandwiches, and Salad Pots” and “Frozen Breakfast Foods, Fruit, and Pastries” both had low estimated environmental impacts and good nutrition qualities, which is in contrast to the Aisles “Pies, Quiches, & Party Foods” and “Sausage Rolls and Party Foods,” which had poor nutrition qualities and moderate to high estimated environmental impacts. Variations in estimated environmental impacts and nutrition qualities were also observed when looking at products within a given Aisle. For instance, the Aisle “Breakfast Cereals” contained products that had on average low environmental impacts, but 12% of the products in this Aisle (32 of 266) had an estimated environmental impact that was among the highest 33% of all of the products at Tesco. These higher-impact breakfast cereals were predominantly granola or breakfast cereals containing chocolate. Conversely, Aisles with high mean impacts often had some lower impact products. For instance, while the Aisle “Pies, Quiches, & Party Foods” had the 11th highest average estimated environmental impact of the 63 Tesco Aisles included in the analysis, 20% of products in the Aisle (29 of 140) had estimated environmental impacts in the bottom 33% of all products at Tesco. These lower impact pies were predominantly vegetarian or vegan, although one lower impact pie contained a small amount of chicken.

### 5.0 Impacts of Similar Foods Are Variable.

**Fig. 6.**
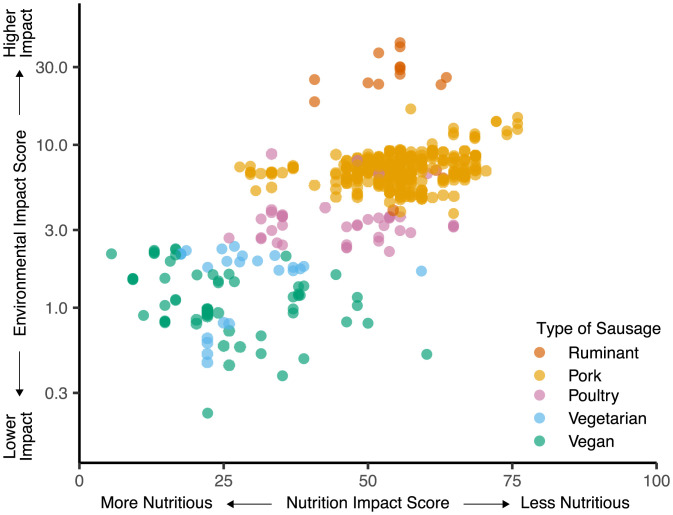
Variation in environmental and nutritional impact of sausages. Each point indicates a single food product, is colored to indicate different food types, and partially transparent to show overlaps of food products with similar environmental impact and nutrition impact scores. In (*A*), point shape indicates whether the pesto sauce contains (circles) or does not contain nuts (crosses). Products were identified based on the retail Aisle and Shelf they were categorized in and their product name. Data sample contains 503 sausages.

Replacing meat, dairy, and eggs with plant-based alternatives could have large environmental and health benefits in places where consumption of these foods is high (1). There are multiple ways to achieve this dietary change, including direct and large substitutions (e.g., beans instead of beef) (28), or smaller transitions between like-for-like products. In some cases, large substitutions may be difficult because of taste preferences, cultural norms, or access to appropriate alternatives. Instead, smaller transitions could be more palatable (29). We therefore examined specific types of food—sausages, pesto sauces, lasagna, and cookies—to investigate how the environmental and nutritional impacts of direct substitutes may vary and how sourcing may affect the environmental impacts of these products (Fig. 6 and *SI Appendix*, Figs. S18 and S19). To identify smaller differences in nutrition quality that may not be possible to identify in NutriScore’s A to E ranking system, we report nutrition quality by scaling the numeric algorithm underlying NutriScore so that it ranges from 0 (best nutrition quality) to 100 (worst nutrition quality).

There were large differences in the estimated mean environmental and nutritional impacts within the identified foods. In many cases, these differences were driven by the presence of one or two ingredients (Fig. 6 and *SI Appendix*, Fig. S18). For sausages, for example, there was a clear difference in the impacts based on the most prevalent meat in the product (Fig. 6). For environment, sausages primarily containing beef or lamb had on average a 240% higher impact (95% CI = 155 to 320%) than pork sausages, which had a 100% higher impact (95% CI = 88 to 116%) than chicken and turkey sausages, which in turn had a 170% higher impact (95% CI = 125 to 208%) than vegan and vegetarian sausages. Sausages composed primarily of beef, lamb, or pork had a 20% higher nutritional impact (95% CI = 12.0 to 28.3%) than chicken and turkey sausages, which in turn had a 75% higher impact (95% CI = 59 to 92%) than vegan and vegetarian sausages (all comparisons using Tukey honestly significant difference [HSD], *P* < 0.05, df = 4). There are also large differences in the environmental impacts of a given type of sausage. For example, within pork sausages, high-impact sausages have a 30% higher environmental impact and a 19% worse nutrition quality.

We found similar trends for pesto sauces, lasagna, and cookies (*SI Appendix*, Fig. S18). For pesto, nuts were determinants of high environmental impacts, while dairy was a driver of poor nutrition quality. In lasagna, the type of meat was a key determinant of its environmental impacts, with beef lasagna having the highest impact, pork and poultry lasagna having intermediate impacts, and vegetarian and vegan lasagna having the lowest impacts. There were no significant differences in the nutrition impact of different types of lasagna. Across the identified cookies, chocolate was a key determinant of both environmental and nutrition, with cookies containing chocolate having, on average, a 13% worse nutrition composition and a 46% higher environmental impact (all comparisons using Tukey HSD, *P* < 0.05). See *SI Appendix*, Supplementary Information Text, for more information.

Changing ingredient sourcing is unlikely to result in meat-based sausages and lasagna having lower environmental impacts than vegetarian and vegan products, but it could result in nut-based pesto and chocolate cookies having lower environmental impacts than their counterparts (*SI Appendix*, Fig. S19). For sausages and lasagna, only in extremes cases in which ingredients in meat-containing products were sourced from among the most efficient production systems and ingredients in nonmeat products were sourced from among the least efficient production systems did meat-containing products begin to have impacts similar to those of nonmeat products (all comparisons made using Tukey HSD test). In contrast, for pesto sauces and cookies, the difference in impacts between product types became nonsignificant even with small changes in sourcing, with larger shifts resulting in nut-containing pestos and chocolate cookies having significantly lower impacts than their counterparts. This indicates how access to transparent sourcing information is needed to fully understand the impact of a food product. See *SI Appendix*, Supplementary Information Text, for additional discussion.

## Discussion

Our algorithm uses publicly available information to derive a first estimate of the environmental impacts of a wide array of food products in a standardized way. Our analyses suggest that the algorithm reasonably estimated the environmental impacts of the diverse set of food products in our data sample even when limited information on the percent composition of their ingredients was known. Similarly, sensitivity analyses show that lack of sourcing information is unlikely to have a large influence on the estimated environmental impact for most products, but that it remains a limitation to fully understanding the impacts of different foods. This analysis expands on previous work that used standardized recipe information to calculate the estimated environmental impacts of 200 meals (30), or alternatively, used ingredient lists to assess the amount of added sugar in premade foods (31). It does so by developing a standardized approach to estimate the ingredient composition, and then the environmental impacts of >57,000 unique food products available in the United Kingdom and Irish retail markets.

Combining the algorithm with a metric of nutrition quality suggests that across retail categories, many of the most nutritious food (but not drink) categories are also among the most environmentally sustainable. This greatly expands on analyses that found similar correlations among single-ingredient food commodities (11, 22). It also indicates there does not need to be a tradeoff between nutrition and environment (although many foods can deliver a win for environment and a loss for nutrition), although whether there is a tradeoff between environment, nutrition, and the broader array of outcomes associated with food consumption (e.g., economic costs, acceptability, enjoyment) remains unclear. Our analysis on similar foods shows that reducing food-related environmental impacts may be possible by food swaps between similar products (e.g., within sausages, pesto, lasagna, and cookies) when larger and more rapid dietary transitions may not be palatable or possible.

The ability of the algorithm to derive estimates of the environmental impact of food products relies on publicly available information. Because the ingredient composition and sourcing of food products is not available and is likely to remain a trade secret, this algorithm or its successors is likely to retain importance. However, it will also remain limited and provide first approximations of each product’s impact until this information becomes more transparent and readily available. The algorithm uses prior information provided on back-of-pack ingredients lists to assess the composition ingredients where this information is unknown. In the United Kingdom and Ireland, listing the percent composition of some ingredients is legally required. The effectiveness of this algorithm in other countries like the United States, where only the rank order of ingredients is required, would be aided by quantitative packaging information on major ingredients. Additional information on ingredient sourcing, such as country of origin or agricultural production method, would help increase the accuracy of the environmental impact estimates (14, 32). In addition, while the environmental database we used contains information from >30,000 food production systems, it also has limitations and biases. This includes a bias toward the commodities grown and production systems found in high-income regions. However, the primary environmental database used in this analysis (13, 14) is constantly growing, which means updated versions of the environmental database can be integrated in the algorithm to reduce these biases. While we have applied the algorithm to products available at food retail stores, it could be modified to provide estimates of the environmental impacts of meals prepared at home or in restaurants. This information could then supplement existing tools such as the Cool Food initiative (33).

Our work focuses on the environmental, and to a lesser extent, the nutritional, impact of foods. However, food impacts human society in numerous ways, including on natural, social, human, and produced capitals. A fuller cost accounting of these capitals is needed, and would enable future work to assess potential tradeoffs between them, such as the interplay between environment, nutrition, accessibility, and affordability. This would be particularly important to understand how communicating product-level environmental impact information may affect the food choices and well-being of different demographic groups. It could also help inform discussions with retailers, processors, producers, consumers, and policy makers on how tradeoffs between the multiple types of capital may be mitigated.

Assessing and communicating the environmental impacts of food products will be integral to achieving the food system transformations that are urgently needed to prevent rapid environmental degradation (1). These impacts are currently unknown for most products because the composition of their ingredients and their sourcing is not fully known. The algorithm developed here could help enable this transformation by providing a framework that derives first estimates of the environmental impacts of food products in countries with ingredient list regulations that are similar to those in the United Kingdom. The environmental impact estimates, either directly derived from packaging information or by using additional information provided by food retailers and processors, could be communicated to promote more environmentally sustainable decision making by consumers, producers, processers, retailers, and policy makers (23, 34–38). The most effective way to communicate a food product’s environmental impacts needs to be determined, including what portion size to use for different products, which environmental indicators to use, how uncertainties in a product’s environmental impacts may be communicated, and whether multiple environmental indicators should be aggregated into a single composite environmental impact score.

## Materials and Methods

### Estimating Product-Specific Health and Environmental Impacts.

We integrated back-of-package information with publicly available environmental (14) and nutritional databases (39) to derive estimates of the environmental and nutritional impacts of food products (Fig. 1). In total, this allowed us to estimate the impacts for 57,185 unique food products available for purchase in a 2-week period in October 2019 from the online stores of at least one of eight UK- or Ireland-based food retailers (Tesco, Sainsbury’s, Asda, Ocado, Iceland, Morrisons, Waitrose, Cook, and Tesco Ireland). We did not include seasonal foods (e.g., Halloween confectionaries) to avoid skewing results from products not consistently available for purchase, and did not include alcoholic beverages because NutriScore does not account for the impact that alcohol consumption may have on nutrition and health.

To estimate each food product’s environmental and nutritional impact, we first estimated the percent composition of each ingredient in each product. We did this in five steps. These are described briefly below, but see also Fig. 1 and the *SI Appendix*, Supplementary Information Text:

(1) We collected back-of-package information from the website of food retailers using fooDB (17).
(2) We identified individual ingredients and the percent composition for ingredients when this information was provided in each product’s ingredient list.
(3) We sorted all of the ingredients into 1 of 110 food categories to integrate with environmental (13–15) and nutritional databases (39).
(4) We estimated the percent composition of the remaining ingredients in the product using prior known information for that product, from similar products, and a series of logic checks to ensure the estimated composition meets UK food labeling regulations. We presented estimates for products for which at least 75% of total composition was sorted into a food category to avoid skewing results.
(5) We paired the estimated ingredient composition with environmental and nutritional databases to estimate the environmental and nutritional impact per 100 g of product. In most cases, with the exception of British beef, sourcing information was not readily available. As such, for environment, we estimated each product’s environmental impact using a Monte Carlo simulation whereby farm-level environmental performance data were repeatedly and randomly selected based on that farm’s representation of global food production systems. This allowed us to derive estimated mean environmental impacts for each product, as well as the variance around the mean and at certain quantiles (5th, 10th, 25th, 50th, 75th, 90th, and 95th). See *SI Appendix*, Supplementary Information Text, for more information.

### Environmental Database Food Categories Used in the Analysis.

We used 111 environmental database food categories in this analysis (109 environmental categories, plus water and salt). These categories were drawn from information available in Poore and Nemecek, HESTIA, and the Blue Food Assessment (13–15). We made unique categories for agricultural commodities that had at least five environmental data observations. We aggregated the remaining commodities (e.g., those with fewer than five data observations) into broader categories (e.g., “other vegetables,” “other fruits”).

### Organic Ingredients and Organic Foods.

We identified organic ingredients and organic foods, pairing these with organic production systems when possible. As with the environmental database food categories, we made this pairing as long as there were at least five data observations of organic systems for the food category, drawing from these organic production systems during the Monte Carlo analysis. In cases in which there were fewer than five observations, we instead drew from all of the production systems for that commodity during the Monte Carlo analysis.

### Building a Composite Environmental Index.

We condensed the four environmental indicators into a single composite index, placing equal weight on each indicator. To derive this composite index, we first scaled each of the indicators so that they ranged from 0 to 100. For each indicator, a score of 100 indicates the product with the highest impact for that indicator, a score of 50 indicates a product with half the impact, and a score of 10 indicates a product with one-tenth the impact of the highest impact product. These scaled scores were then averaged and further rescaled so that they ranged from 0 to 100. This resulted in the composite environmental index, in which, on average across the environmental indicators, a product with a score of 20 had ⅕ the impact of the highest impact product and a product with a score of 2 had ^1^/_50_ the impact of the highest impact product.

There are alternative methods to aggregate indicators based on economic valuation, expert opinion, or proximity to environmental targets (40). As such, we also provide estimates for each environmental indicator. See the *SI Appendix*, Supplementary Information Text, for more information.

### Calculating the Nutrition Impact Score.

NutriScore ranks products from −15 (least harm and most nutritious) to 40 (most harm and least nutritious) based on the content of 7 components, penalizing products for 4 components (energy, saturated fat, sodium, and sugars) and rewarding products for 3 components (protein, fiber, and the percentage of the product that is fruits, vegetables, nuts, olive oil, walnut oil, and rapeseed oils). The penalized components are given a score ranging from 0 to 10 based on preset nutrient density thresholds, whereas the rewarded components are given a score ranging from 0 to 5. The positive score (the sum of the 0 to 5 scores) is then subtracted from the negative score (the sum of the 0 to 10 scores), resulting in a possible range of −15 to 40. This is then translated into the A to E NutriScore, with different thresholds for different types of food (e.g., cheese, beverages, fats). See *SI Appendix*, Supplementary Information Text, for more information.

### Testing the Method.

We focused on testing the environmental impact score of food products, rather than the NutriScore as it is an established scoring system and because most nutrition information required to calculate the NutriScore is legally required to be provided on back-of-package in the United Kingdom.

First, we identified all of the products for which the percent composition for all or all but one ingredient was provided, for which the product contains more than one ingredient, and for which the sum of the provided percent composition equaled 100 (*n* = 1,547 products). For these products, we calculated the mean environmental impact score and variance around the mean using provided back-of-package information and a Monte Carlo analysis as described above and further described in the *SI Appendix*. We call this the “known environmental impact score” because all of the information required to estimate a product’s environmental impact score was fully known.

Second, we estimated the mean environmental impact score and the variance around the mean estimated impact for the same 1,547 products using the algorithm developed here when assuming that randomly selected subsets of composition information for *n* ingredients was not known, where n ranges from 2 ingredients to all ingredients in the product. We call these estimates the “estimated environmental score” because they were estimated based on imperfect ingredient composition information for the products. We then (1) estimated the percent composition of the n ingredients in the product (e.g., those ingredients for which the composition was assumed to not be known); (2) estimated the environmental impact score based on the estimated composition from (1) using the Monte Carlo analysis described above; and (3), repeated the process above for up to 100 different randomly selected unique combinations of n ingredients.

Finally, we compared the difference between the known and estimated environmental impact scores. We reported accuracy as the log ratio between the estimated and known environmental impact scores (calculated as log2(estimated environmental impact/known environmental impact)). We further assessed the difference between the known and estimated environmental impact scores by examining how often the uncertainty around the environmental impact scores (e.g., as may occur from sourcing or food production methodology and identified in the Monte Carlo analysis) overlap for the known and estimated scores.

### Categorizing Food Products.

We categorized products into Departments, Aisles, and Shelves using the classification systems available on the website of each food retailer and that are unique to each food retailer. This system increases in specificity from Departments (e.g., “Bakery”) to Aisles (e.g., “Bread”) to Shelves (e.g., “Whole Wheat Bread”). In this classification system, some products are categorized into multiple Departments, Aisles, or Shelves. For instance, a loaf of whole meal bread could be categorized into the Department “Bakery,” the Aisle “Bread,” and the Shelf “Whole Meal Bread.” Similarly, the same product could also be classified into the Aisle “Our Bakery” (e.g., the retailer’s in-store bakery) and the Shelf “Fresh Loaves.” When estimating the environmental and nutritional impact of Aisles (as in Figs. 4–6), we included these products in the mean estimate of each Aisle into which the food has been categorized by the food retailer.

### Environmental Impact Score Per Serving.

We estimated the environmental score per serving, as in *SI Appendix*, Fig. S8. To do this, we extracted serving information for each product when provided. For products for which serving size information was not available, we estimated their serving size using data from similar products by using the average serving size of products in the same “Shelf” if this information was available, and if not available, by using the average serving size of products in the same “Aisle.” For products that were classified to multiple Shelves or Aisles, we took the average estimated serving size for that product across all Shelves and Aisles to which that product was categorized. We then recalculated the environmental impact score per serving using the process in the main text and the *SI Appendix*, Supplementary Information Text.

## Supplementary Material

Supplementary File

Supplementary File

## Data Availability

The algorithm and associated data inputs are available at Oxford Research Archives (https://ora.ox.ac.uk/objects/uuid:4ad0b594-3e81-4e61-aefc-5d869c799a87) (41). Due to legal constraints, the product-level data available at the above link is anonymized. A nonanonymized version of the product level data are available under license upon request from R.H. and P.S. To request a nonanonymized version of the product-level data used in the analyses for the purpose of replicating findings, please email foodDBaccess@ndph.ox.ac.uk. Anonymized (algorithm and associated data inputs, anonymized version of the product level data used in the analysis) data have been deposited in the Oxford University Research Archive. Some study data available (a nonanonymized version of the product-level data are available under license upon request from R.H. and P.S.; to request a nonanonymized version of the product-level data used in the analyses for the purpose of replicating findings, please email foodDBaccess@ndph.ox.ac.uk.).

## Code Availability.

All of the code used in the analysis will be made freely available before publication.
