# Supplementary material for: Estimating the environmental impacts of 57,000 food products
Source: Proc Natl Acad Sci U S A. 2022 Aug 8;119(33):e2120584119. doi: 10.1073/pnas.2120584119 (PMC9388151; doi:10.1073/pnas.2120584119)
Supplement: Supplementary File [file pnas.2120584119.sapp.pdf]

PNAS
www.pnas.org

Estimating the environmental impacts of 57,000 food products

Michael Clark[1,2,3,4]*, Marco Springmann[1,2], Mike Rayner[1], Peter Scarborough[1,5], Jason Hill[6], David Tilman[7,8], Jennie I. Macdiarmid[9], Jessica Fanzo[10,11], L Bandy[1,12], Richard A Harrington[1,5]

Michael Clark
Email: michael.clark@smithschool.ox.ac.uk

This PDF file includes:

Supplementary text
Figures S1 to S19
Tables S1 to S7
Legends for Datasets S1-3
SI References

Other supplementary materials for this manuscript include the following:

Datasets S1-3

Supplementary Information Text

Methods:

Obtaining Back of Package Information:

The raw data used in this analysis was obtained from food retailers as described in Harrington et al. (2019)(1). Harrington et al. created a web tool (foodDB) to collect data for food products available for purchase from food retailer's websites. FoodDB has been collecting data from food retailers on a weekly basis since November 2017. The data extract used in this analysis is a 2-week extract from October 2019 that covered eight U.K.-based or Ireland-based food retailers: Cook, Iceland, Morrisons, Ocado, Sainsbury, Tesco, Tesco Ireland, and Waitrose. In total, this extract included 225,872 unique entries from 88,008 unique products.

For each data entry, the information used in this analysis includes: the product id (an internal tracking mechanism, this does not match the product's stock keeping unit); product name; product list name (same as the product name); url (the url from which information was obtained, used to identify the food retailer); product categorization as used by the food retailer, including Department (e.g. "Bakery", Aisle (e.g. "Bread"), and Shelf (e.g. "Whole wheat bread"); ingredients text (list of ingredients); fat per 100g; saturated fat per 100g; salt per 100g; sugar per 100g; carbohydrates per 100g; protein per 100g; and fiber per 100g. Because products can be categorized into multiple Departments, Aisles, and Shelves, there are more entries than there are unique products.

We focused our analysis on food and non-alcoholic beverages (or milk alternative) products, and did not include seasonal products (i.e. Christmas or Halloween confectionaries) to avoid skewing our the analyses.

Identifying Ingredients and Known Percent Composition:

Back-of-package ingredient lists were provided as a single string of text for each product. We separated these lists to identify individual ingredients and their known percent composition (when listed) in four steps.

First, we removed text that indicated allergen information (for example "Allergens: For allergens see ingredients in bold") and ingredient sourcing (for example "* indicates organically produced ingredients" or "* indicates fair trade certified").

Second, we identified and extracted embedded ingredient lists. We define an embedded ingredient list as a list of ingredients that compose a larger ingredient in a food product. For example, if the ingredients text for fortified wheat flour was "Fortified wheat flour (wheat flour, calcium carbonate, iron, thiamine)", then the embedded ingredient list is "(wheat flour, calcium carbonate, iron, thiamine)". We extracted all embedded ingredients lists for each food product, saving them for later use (see "Estimating Percent Composition of Other Ingredients").

Third, we separated ingredients lists into individual ingredients. Individual ingredients were separated by commas or semi-colons, which were placed after the percent composition for that ingredient if it was provided (for instance, "Fortified wheat flour (39%), Milk, Eggs,…"). To identify individual ingredients, we separated the ingredients text into individual ingredients by separating the ingredients text based on the location of commas and semi-colons. When separating ingredients into individual ingredients, we created a placeholder variable to indicate the location of that ingredient in the ingredient text. For instance, "V1" indicated the first ingredient in the list, "V2" the second ingredient, "V3" the third ingredient, etc. We also separated ingredients in the embedded ingredient lists, labeling these as for example "V2.1" (for

the second identified ingredient in the product, the first ingredient in the embedded ingredients list) “V2.2” (for the second identified ingredient in the product, the second ingredient in the embedded ingredients list), “V3.1” (for the third identified ingredient in the product, the first ingredient in the embedded ingredients list), etc.

Fourth, we identified the percent composition for ingredients when this information was provided. We did this by searching and then extracting this information using the R function “str_extract_all” and the search term "[0-9]{1,3}(\\s)?%|[0-9]{1,3}(\\.)[0-9]{1,2}(\\s)?%". The function “str_extract_all” extracts all instances of text that meet the search term. In instances where two or more percent values were extracted by the search term for a given ingredient in a food product, we performed a series of logic checks to decide which extracted value to use. These logic checks were: (1) the percent composition of an ingredient cannot be more than 1/n * 100, where n is the location of the ingredient in the ingredient text; (2) the percent composition of the ingredient needs to be equal to or greater than the percent composition of all ingredients that appear later in the ingredient text; (3) the percent composition of the ingredient needs to be equal to or less than the percent composition of all ingredients that appear earlier in the ingredient text; and (4) the percent composition of the ingredient needs to be less than or equal to (100 – sum(known percent composition other ingredients)). If multiple extracted values for an ingredient met all four listed criteria, we then took the average of these values. We also extracted the percent composition for ingredients in the embedded ingredient lists.

Estimating Percent Composition of Other Ingredients:
We used a combination of prior known information from each product, prior known information from similar products, and a series of logic checks to derive estimates of the percent composition of ingredients where this information was not provided. The prior known information we used is the 10.4% of ingredients that had a percent composition listed in the ingredients list, the nutrition information of each product, and how these products were sorted into Departments, Aisles, and Shelves by food retailers.

First, after estimating the composition of ingredients in a product by using information from similar products, we estimated the composition of salt in a product. We did this by first identifying ingredients in a product that are salt (e.g. salt, sea salt, etc). Then, if the amount of salt in the food was provided in the nutrition information and the percent of salt was listed, we set the percent composition of salt in that product to be equivalent to the total salt content in the product. We did not update the estimated percent composition if instead the estimated composition of salt was provided. For products where the percent of salt was estimated using back of package information, we allowed for this estimated composition to be updated in the series of logic checks described below as other ingredients may also contain salt (e.g. cheese).

Second, we used nine approaches to derive a first estimate of the composition of each ingredient in each product. These are: (1-3), the average composition of that ingredient when it is in the same location in the ingredients list in other products categorized into the same Shelf (1), Aisle (2), or Department (3) by a Retailer; (4-6), the average composition of the nth ingredient of other products categorized into the same Shelf (4), Aisle (5), and Department (6); and (7-9) a series of linear (y = intercept + n) and power law regression equations (y = intercept + 1/n or y = intercept + $1/n^2$) that estimated the composition of the nth ingredient for each Shelf (7), Aisle (8), and Department (9). For approaches (1-6), we only applied use this approach when at least ten known percent compositions were identified for the nth ingredient within each food category within that Shelf, Aisle, or Department to avoid introducing potential bias from small sample sizes. For (7-9), each of the three regression forms were fit, and then the functional form with the highest adjusted r^2 of the three functional forms was used, but only if (a) the adjusted r2 was greater than or equal

to 0.75, (b) the relationship between the estimated composition and the order of ingredient was significant at $P < 0.05$, and (c) if there was known composition information for the nth ingredient was known for at least 50% of the maximum observed number of ingredients in a product in that retail category. In other words, if a product in that retail category contained 15 different ingredients, then this approach was only used if composition information was provided for at least 8 ingredient positions (e.g. the first ingredient, second ingredient, etc…)

Third, the accuracy of each of these approaches was then tested against provided composition information for that retail category. For approaches (1-3), the accuracy was based on the ingredient category (e.g. tomatoes, wheat, apples, brassicas, etc; see below in "Linking to Databases") and the ingredient position, whereas for approaches (4-9) the accuracy was only based on the order of the ingredient in the ingredient list. For each approach, the accuracy was calculated as the percent difference between the estimated percent composition and the provided percent composition for that ingredient.

Fourth, the most accurate of the above approaches was then used to derive a first estimate of the composition of an ingredient. When doing this, preference was first given to approaches using information from products in the most similar retail categories (e.g. Shelves and Aisles) and approaches that used ingredient category specific information, such that the preference of the approaches described above was as follows: 1, 2, 4, 5, 3, 6, 7, 8, 9. However, these approaches were only used to derive a first estimate if the average accuracy of that approach was within a given accuracy threshold, such that the first approach within the lowest accuracy threshold was used to derive the first estimate of an ingredient's composition (thresholds were set at 10%, 20%, 25%, 30%, 40%, and 50%). As such, if approaches 1-6 had an average accuracy >10%, whereas approach 7 had an accuracy <10%, then approach 7 was used to derive the first estimate. Alternatively, if approach 1 had an accuracy of 7% whereas approach 2 had an accuracy of 5%, approach 1 was used because it was built on composition information from the most similar products possible. If none of the approaches had an accuracy of less than 50%, then the percent composition of that ingredient was estimated using steps five and six described below. Because the approach used to derive the first estimate of an ingredient's composition was based on that approach's average accuracy, which often varied by ingredient order and ingredient category, it is possible multiple different approaches were used to derive a first of different ingredients in a given product. Importantly, this framework ensures that the algorithm will be more scalable to other data sets, as the most accurate approach will be automatically selected, and that the most accurate approach(es) for the data used in this analysis may not be the most accurate approach(es) for other data sets.

Fifth, for ingredients where the percent composition could not be estimated using the above steps, we estimated the percent composition in a series of four steps. (1) If the composition of the first ingredient is not known and could not be estimated as described above, and if the composition of the last ingredient could not be estimated using the process above, we assumed the last ingredient in the food product accounted for 0.1% of the total composition to avoid overestimating its composition of the entire product. (2) We linearly interpolated between composition values, both known and estimated as described in the previous paragraph and in step (1). For instance, if the third ingredient was estimated to account for 10% and the fifth ingredient was estimated to account for 5%, then we estimated the composition of the fourth ingredient as 7.5% of the product. (3) If the composition of the first ingredient is known or could be estimated as described in the previous paragraphs, and the composition of the last ingredient was not known, we assumed that the last n ingredients (where n indicates the number of ingredients where the percent composition was neither listed nor could be estimated as described in the previous paragraph) accounted for the remaining composition of the product. (4) If the composition of the first n

ingredients was not known or not possible to estimate as described in the above paragraphs, we assumed that these ingredients accounted for the remaining composition of the product. Note that steps (2), (3), and (4) are mutually exclusive, and cannot be used for the same ingredient.

Sixth, we used a series of three iterative logic checks. These logic checks are as follows: (1) The composition of the first ingredient must be equal to or greater than the composition of the second ingredient, the composition of the second ingredient must be equal to or greater than the composition of the third ingredient, etc; (2) the composition of the nth ingredient cannot be greater than 1/n * 100 (if this were the case and the composition of each ingredient counted for at least as much as the next ingredient, then the total composition of the product would exceed 100%); and (3) the composition of all ingredients must sum to 100. We then repeated these logic checks until all conditions were met, only adjusting the percent composition of ingredients for which the percent composition was not provided.

If in the process of these iterative logic checks the percent composition of an ingredient where the percent composition was not provided was ever equivalent to either of (a) the composition of the next product in the ingredient list where the composition was provided, or (b) equivalent to the composition of the preceding ingredient where this information was provided, we assume that the estimated percent composition of the ingredient could not be further adjusted. In addition, we also assumed the percent composition of the last ingredient (when not already provided) could not be further adjusted. We necessarily had to do this to anchor the algorithm, as not doing this occasionally resulted in estimated percent composition values that could not be true (for instance, some ingredients had a negative percent composition, or the last ingredient accounted for a larger amount of the product than the second to last ingredient).

We repeated this same process for the composition of ingredients in embedded ingredient lists. To calculate the final percent composition of embedded ingredients, we multiplied the estimated percent composition of the non-embedded ingredient with the estimated composition of each embedded ingredient within that ingredient. For instance, if the second ingredient is pasta sauce, is estimated to have a percent composition of 30%, and has embedded ingredients of "tomatoes, onion, garlic, olive oil, salt" with estimated percent compositions of 70%, 15%, 10%, 4%, and 1%, respectively, then the estimated percent composition of the embedded ingredients was estimated to be 21% (30% * .7), 4.5% (30% * .15), 3% (30% * .1), 1.2% (30% * .04), and .3% (30% * .01), respectively.

For some food products, the back-of-package ingredient list indicated the total composition of certain ingredients (this was particularly common for sauces, jams, and jellies). For example, the ingredient list for ketchup might have stated "This product contains 300g tomatoes per 100g product", or the ingredient list for a jam or jelly might have stated "This product contains 150g fruit per 100g product". We identified these ingredients and their total composition by searching the ingredient list using the search terms "[0-9]{1,3}(\\s)?g(\\s)per(\\s)?[0-9]{1,3}" and "[0-9]{1,3}(\\s)?g(\\s)per(\\s)?[0-9]{1,3}", discounting any results that returned "100g", and then identifying the food category of this (or these) ingredients by using the process described above.

For products with one listed ingredient, the percent composition of this ingredient was assumed to be 100% of the product.

To avoid skewing results with products that contained a large portion of unsorted ingredients (for instance, due to misspellings in the ingredient list), we presented estimates only for those products where at least 75% of their total composition by mass were recognized and sorted into one of the food categories used to link to the environmental and nutrition databases (see "**Linking**

Ingredients to Databases". As such, the sample size in this analysis was 57,185 products included in the analysis is less than the 88,008 unique products identified in our data extract. Many of the products excluded from the analysis were non-food items (e.g. wine and beer glasses, soaps, shampoos, and alcohols). The most common ingredients sorted into each of the environmental database food categories are available in **Dataset S1.**

Linking Ingredients to Databases:
Products with ingredients:
We linked ingredients to published environmental (2–4) and nutrition databases (5). A description of these databases can be found in the sections "Description of the Environmental Databases" and "Description of the Nutrition Database".

To do this, we created food categories based on data available in environmental databases (hereafter called environmental database food categories). We identified these categories based on agricultural commodities that had 5 or more observations in the environmental databases. This resulted in a total of 110 environmental database food categories (109 environmental categories, plus water and salt to result in 111 total categories). For each of these food categories, we derived a list of search terms to sort ingredients into one of the 110 environmental database food categories sorted each ingredient into one of the 110 food categories listed in the environmental database. We did so using a three-step process based on regular expressions and search terms for each food category. The list of search terms used is available in the **Dataset S2.**

The first step was to use the search terms to count the number of times an ingredient was sorted into each environmental database food category. During this step, a given ingredient could count towards the total for multiple food categories. Note that ingredients were not sorted into categories during this step.

The second step was to repeat this process, but categorizing ingredients into one of the environmental database food categories, starting with the category that had the fewest identified ingredients and cycling through categories based on increasing number of ingredients that were identified in the first step. As such, the second food category that was cycled through was the one with the second fewest ingredients identified in the first step, etc. Ingredients were sorted into the first food category possible. This sorted ingredients into one of the 52 primary environmental food categories (e.g. 'Wheat & Rye').

The third step was to take the results from the second step, and then further classify ingredients into sub food categories based on an additional set of search terms. For instance, this step could reclassify 'Wheat & Rye' to 'Bread', 'Rye', or 'Wheat'.

For example, imagine an ingredient in a product was matched by search terms for the environmental database food categories "fish" and "crustaceans". In the first step described above, this match would count towards the number of ingredients that qualify for each food category, but would not be sorted into either the "fish" or "crustaceans" category. After the first step, the food category "fish" was matched by 1,234 ingredients, while the food category "crustaceans" was matched by 321 ingredients. During the second step, ingredients are sorted into food categories based on the increasing amount of ingredients that qualify for each category in the first step (in this example it would be sorted into the category of 'Crustaceans' because there were fewer observed ingredients that were identified as a potential crustacean). In the third step, the ingredient would be further classified into a sub category of crustaceans (e.g. 'Prawn', 'Shrimp', etc).

After completing the above, we identified ingredients that were listed as water and salt. We then assigned these ingredients to one of these categories, but only if that ingredient had not already been sorted into one of the food categories used in this analysis.

Because the environmental database contains categories for soymilk, oat milk, rice milk, and almond milk, we instead estimated the environmental impact of these products based on their product name.

This process was completed individually for each Department within each Retailer.

Checking non-matched ingredients:
After sorting ingredients into one of the environmental database food categories, we examined the 1,000 most common ingredients that remained unsorted. We did this to ensure that the search criteria and search terms we developed adequately identified food ingredients and left non-food ingredients (e.g. vitamins, minerals, preservatives, leavening, etc.) unmatched.

In total, there were 629,007 observations of the 1,000 most common unsorted ingredients, compared to a total of 774,236 unsorted ingredients (or 81.2% of unsorted ingredients) (**Dataset S1**). The majority of these 1,000 most common unsorted ingredients were food additives (e.g. "Calcium carbonate", "Dextrose", "Citric acid"; 265,828 observations, or 42.3%), flavouring (e.g. "Flavouring", "Natural Flavouring" "Smoke flavouring", etc); 79,204 observations, 12.6%) vitamins or minerals (75,443 observations, 12.0%), spices (73,078 observations; 11.6%); leaveners (e.g. yeast, baking soda; 44,665 observations, 7.1%), potential foods, but without adequate information to sort into one of the environmental database food categories (e.g. "Sweeteners", "Palm", "Whole powder"; 30,918 observations, 4.9%), and other ingredients (e.g. bacteria cultures such as "Lactobacillus Acidophilus", or alternatively ingredients without adequate information to identify such as "()", "in varying proportions", or "total"; 22,322, 3.5%).

There were some potential food ingredients that were matched with the environmental databases. These included vinegar (10,878 obervations; 1.7%), hard alcohols and spirits for which environmental information was not available (604 observations, 0.1%), Other information, such as messaging (e.g. "Certified Organic" and "Rainforest Alliance"; 986 observations, 0.2%) was also identified in this search.

Products without ingredients:
We used a different process for products that did not have an ingredient list. We also used search terms for these products, but used a different list of search terms because of different naming conventions between product names and ingredients, but also increased specificity in product names (see **Supplemental Data** for a list of these search terms). For example, white bread may appear in an ingredient list as its constituent ingredients (wheat flour, salt, yeast, etc), but may be named as "baguette", "baton", "bap", "tiger loaf", etc.

We also used a two-step process for products without listed ingredients. As described above, the first step sorted products into food categories that were used to pair products with the environmental and nutrition databases, and then counted the number of products that were sorted into each food category. This step was only used to count products that met the criteria for each food category, and was not used to identify the food category for a given product.

During the second step, we sorted food products into food categories, starting with the food category with the fewest number of products and finishing with the food category with the largest number of products. In contrast to when sorting ingredients into food categories, we also used

search terms that disqualified products from certain food categories. For example, a product named "Gluten free white bread" would meet the search criteria for the food category "Wheat", but would then be disqualified from the food category "Wheat" because gluten free bread does not contain any wheat.

This process was repeated simultaneously across all products from all Retailers.

Description of the Environmental Databases:
The environmental data used in this analysis are derived from a meta-analysis of life cycle assessments (LCAs)(2). Life cycle assessments estimate the environmental impact of food production by tracking the inputs (e.g. fertilizer, pesticide, energy use) used during food production (6). Meta-analyses of life cycle assessments aggregate and synthesize data from individual LCAs to provide estimates of a food's environmental impact. For this analysis, we used data available from Poore and Nemecek (2018) (2), which is being converted into a constantly growing online environmental database named HESTIA (3). The Poore and Nemecek (2018) database contains data from over 40,000 food production systems. Because Poore and Nemecek (2018) contains limited information on capture fish, we supplemented it with information from the Blue Foods Assessment. The food commodities from Poore and Nemecek (2018) and the Blue Foods Assessment were then condensed into 110 food categories. We condensed the production systems into food categories, such that every food category had at least 5 unique observations. Commodities with fewer than 5 unique observations were grouped together to create a larger category, such as 'Other Fruits'.

We further identified organic systems, as recorded Poore and Nemecek (2018) (2). When possible, we paired organic ingredients and organic products with organic life cycle estimates, but only when at least 5 observations of production systems for that ingredient were available. If there were fewer than 5 organic observations for that ingredient, we instead randomly sampled across all production systems during the Monte Carlo analysis (described below).

Data from LCA meta-analyses are biased by geographic coverage and by representation across food commodities(2), although the environmental databases used here used weights and reconciliations to correct for this bias when possible. Most LCAs are conducted in middle- or higher-income countries, with comparatively sparse coverage in lower-income regions and particularly in Sub-Saharan Africa and Central Asia. In addition, LCAs primarily examine the environmental impacts of higher-value and/or widely produced food products, with comparatively little coverage on lower-value and/or less widely produced foods (e.g. quinoa).

Due to this inherent bias in LCA meta-analyses, and because sourcing information is not available for most food products, we estimated the mean environmental impact for each product and each indicator using a Monte Carlo analysis as described below. This further stresses the need for better environmental data on food production systems.

Estimating the environmental impact of fish:
Because Poore and Nemecek (2018) (2) does not contain information on capture fish, we supplemented it with data from the Blue Foods Assessment (4). However, results from the Blue Foods Assessment are provided as mean estimates and 95% confidence intervals. As such, we randomly sampled 100 data points (e.g. production systems) from within the 95% confidence intervals to supplement the data from Poore and Nemecek (2018) (2). We further weighted the likelihood for capture fisheries to be randomly sampled during the Monte Carlo analysis (as is already done in HESTIA and Poore and Nemecek (2018)) using fishery capture information available in the most recent FAO State of the World Fisheries and Aquaculture (7).

To estimate the impact of seafood products, we assumed a 50%:50% split between capture and farmed fish, which is in line with recent FAO estimates (7). As such, during the Monte Carlo analysis described below, we ensured that one half of the production systems for seafood products were from capture fisheries, with the remaining half from aquaculture systems.

Description of the Nutrition Database:
Most nutrition data used in this analysis was derived from back-of-package information. However, when necessary, we supplemented the provided data with data from the European nutrient composition tables available from GENuS (5).

We derived average nutrient composition values for each of the food categories in two steps. First, we sorted entries from GENuS into food categories using the search process described above. Second, we took the mean nutritional value of foods that were sorted into each of the 52 food categories.

Estimating Environmental Impacts of Food Products:
We next derived first estimates of the environmental impact score of each food product. We did this by using the estimated percent composition of each ingredient in each food product and a Monte Carlo analysis that randomly selected producer-level environmental performance data per 100g of food produced from the life cycle database. In this Monte Carlo analysis, environmental performance data from a randomly selected food production system for each commodity was used to estimate the impact of that commodity in a food product, where selection of production systems was weighted based on their share of global production. This process was repeated 1,000 times to derive mean environmental impact estimate for each product and the variance around it for each indicator, as well as the minimum and maximum potential impact for a product. This Monte Carlo analysis provides a sense of how uncertainty in ingredient sourcing might influence a product's estimated environmental impact.

For seafood, we assumed that 50% of the randomly sampled points were from capture fisheries, with the remaining 50% from farmed systems, which is in line with recent FAO statistics (7).

For organic ingredients and products, we paired organic ingredients with organic production as long as there were more than 5 organic production systems available in the life cycle databases. We set a lower limit of 5 organic observations to ensure that there was variability in the randomly sampled environmental impact estimates during the Monte Carlo analysis. For food commodities with fewer than 5 organic observations, we instead randomly sampled across all production systems for that commodity.

We used the Monte Carlo analysis to derive a first estimate of the environmental impact of each food product for four environmental indicators: greenhouse gas emissions; land use; scarcity weighted water use; and eutrophication potential. Greenhouse gas emissions provide an estimate of that food product's impact on climate change, and is measured as grams of CO_2e. Land use is an estimate of how much arable land and pastureland is occupied to produce a unit of food per year. Scarcity weighted water use weights water use by regional water availability, such that using large amounts of water in a relatively wet location might have a low scarcity weighted water use, whereas using small amounts of water in an arid location could result in a high scarcity weighted water use (8). Eutrophication potential measures the runoff of nutrients from land into water, and the resultant potential eutrophication (or excess nutrient richness) in aquatic environments (9).

The method used to estimate the percent composition of food products iterates through each food retailer, and then through departments within that food retailer. As such, there are multiple entries for food products that are available from multiple Retailers, or for products that have been categorized into multiple Departments, Aisles, or Shelves at a given Retailer. For these products, we took the mean value of their estimated environmental impact.

We then scaled the estimated impact for each of these four environmental indicators such that they ranged from 0 (no impact) to 100 (highest impact). For each food product, we did so by dividing the estimated environmental impact for that environmental indicator by the highest estimated environmental impact for that environmental indicator across all food products in the database. We call these the "scaled environmental impacts.

We then developed a single aggregate estimate of a food product's environmental impact, which we call the "environment impact score". We did so in two steps. First, we averaged the scaled environmental impact score for each food product. Second, we then divided the resultant environmental impact score by the highest observed environmental impact score, and then multiplied this value by 100. As such, the environmental impact score ranges from 0 (no environmental impact) to 100 (highest environmental impact). We note that our approach of aggregating multiple environmental indicators into one combined index places equal weight on each environmental indicator, and that there are alternative methods to aggregate indicators based on economic valuation, expert opinion, or proximity to environmental targets, as well as methods to condense environment and nutrition into one combined indicator (10).

Example calculations of a product's environmental impact score:
The first step to calculating a product's environmental impact score is to identify the highest estimated impact for each of the four environmental indicators. The highest estimated impacts are: 20.2kg CO2e for GHGs; 62.2 m^2 of land; 174g PO_4e of eutrophication potential; and 43,600L of scarcity weighted water use.

The second step is to calculate the scaled impact for each environmental indicator. This is done by taking the impact of a product, dividing it by the highest observed impact for an indicator, and then multiplying by 100. If a product has impacts of 3kg CO2, 3.5 m2 of land, 10g PO4e of eutrophication, and 5,600L of scarcity weighted water use, then this would result in a scaled score of 14.9 for GHGs (3/20.2 * 100); 5.6 for land (3.5/62.2 * 100); 5.7 for eutrophication potential (10/174 * 100); and 12.8 for scarcity weighted water use (5,600/43,600 * 100).

The third step is to average the scaled scores. This results in a scaled score of 9.75 ((14.9 + 5.6 + 5.7 + 12.8) / 4).

The fourth and final step is to derive the composite environmental impact score by dividing the scaled score by the highest observed scaled score and then multiplying by 100. The highest observed scaled score was 75.1. As such, for the example product, the composite environmental impact score is thus 13.0 (9.75/75.1 * 100).

Estimating Nutrition Quality of Food Products:
We next estimated the nutrition quality score of each food product. We did so by using the provided nutrition information for each product (in the U.K., most products are mandated to provide information on energy, fat, saturated fat, sugars, salt/sodium, carbohydrates, and protein).

When necessary, we supplemented the provided nutrition information. We did so in three instances. (1) When no nutrition information was provided for that product. (2) When the

nutrition information for one nutrient was not provided for that product. (3) When the nutrition information provided is not possible, for example if (a) the provided caloric content was greater than 900 calories per 100g of product or where there was estimated to be more than e.g. 100g of fat per 100g of product. For these products and nutrients, we instead estimated the nutrition composition by combining the estimated percent composition of ingredients within a product and the estimated nutrient composition of each of the 52 food categories.

We also estimated the percent of the product that is fruit, vegetables, nuts, olive oil, walnut oil, or rapeseed oil. We did so by, for each product, first averaging the estimated percent composition for that product across all Retailers, Departments, Aisles, and Shelves, and then summing the composition of ingredients that qualify as fruits, vegetables, nuts, olive oil, walnut oil, or rapeseed oil.

The nutrient profiling index used in this analysis is NutriScore (11). We used NutriScore in this analysis because of use in France and general support in Europe (12–18), and because dietary adherence to NutriScore is associated with improved health outcomes (19). NutriScore ranks products based on seven aspects: content of energy; saturated fat; sugar; sodium; protein; fibre; and fruits, vegetables, nuts, and some oils(11). NutriScore penalizes products based on their composition of four nutrients commonly associated with poor health: energy, saturated fat, sugars, and sodium content. These nutrients were scored per 100g of product, and were awarded a value of 0 (low composition, "good") to 10 (high composition, "bad") based on predetermined thresholds. Likewise, NutriScore rewards products based on their composition of three nutrients associated with good health: protein, fiber, and percent of the product that is composed of fruits, vegetables, nuts, olive oil, walnut oil, or rapeseed oil. These nutrients are scored on a scale of 0 (none) to 5 based on predetermined thresholds(11).

The NutriScore value of the product can range from -15 to 40, where a score of -15 indicates the best possible nutrition composition, and 40 indicates the worst possible nutrition composition (11). This numeric value is then converted to an A (best nutrition composition) to E (worst nutrition composition) using predefined cutoffs that differ across food types.

When comparing the nutrition impact of a diverse array of food products, we converted the A to E scale such that it ranges from 1 (best nutrition composition) to 5 (worst nutrition composition). This allowed for the nutrition impact of products to be averaged by retail Aisle or Shelf, and then the average nutrition impact of retail categories (e.g. Aisles) to be compared despite the different cutoffs used to convert the numeric scale to an A-E scoring system.

When comparing the nutrition impact of similar food products (e.g. products that have the same cutoffs used to convert from the numeric scale to an A-E scoring system), we instead converted the underlying numeric scale so that it ranges from 0 to 100. This allowed for more finite and granular difference in the nutrition impact of similar products to be identified and then compared. We did so by first adding 15 to the score of each product (so that it now ranges from 0 to 55), then dividing the resultant score by the highest resultant score in our database, and then by multiplying by 100. As such, products with a nutrition quality score of 0 are the most nutritious products while products with an estimated nutrition quality score of 100 have are the least nutritious products in the analysis.

Example calculations of a product's nutrition impact score:
The first step to calculating a product's nutrition impact score (NutriScore) is to collate information on that product's nutrition information for the seven aspects of a food contained in NutriScore: energy; sugar; saturated fat; sodium; fiber; protein; and the percent of the product that

is fruit, vegetables, nuts, or healthy oils (FVNO). Let's assume that 100g of a product contains 400kJ of energy; 3g sugar; .5g saturated fats; 700mg sodium; 5g fiber; 10g protein; and that 30% of the product's composition is FVNO.

The second step is to convert these nutrient values into a numeric score based on preset thresholds. The potential range in the numeric score for energy, sugar, saturated fat, and sodium is 0-10, while the potential range for fiber, protein, and FVNO is 0-5. For each food component, higher scores are given to foods that contain larger amounts of that food component (e.g. high saturated fat foods will receive a higher saturated fat score). For the sample product, this translates into scores of: 1 for energy; 0 for sugar; 0 for saturated fats; 7 for sodium; 5 for fiber; 5 for protein; and 0 for FVNO.

The third step is to sum the negative components (where excess consumption is associated with poor health; these are energy, sugar, saturated fat, and sodium) and to sum the positive components (those associated with health benefits; these are fiber, protein, and FVNO). For the example product, these are a score of 8 for the negative components (1 + 0 + 0 + 7), and a score of 10 for the positive components (5 + 5 + 0).

The fourth step is to subtract the score of the positive components from the score of the negative components. For the example product, this results in a value of -2 (8 – 10).

The final step is to convert the numeric value into the A-E score used in NutriScore. This is based on preset thresholds, such that solid foods are given an 'A' if they have a value between -15 and -1; a 'B' if they have a value from 0 to 2; a C if they have a value of 3 to 10; a D if they have a value of 11 to 18; and a D if they have a value of 19 to 40. There are different thresholds for beverages, such that only water is given a value of 'A'; other beverages with a score of 1 or less are given a score of 'B'; beverages are given a 'C' if they have a value of 2 to 5; a 'D' if they have a value of 6 to 9; and an 'E' if they have a value of 10 to 40.

Note that there are some exceptions to how NutriScore is calculated based on the type of food product. The major exceptions are for solid foods and beverages as already noted above, but there are also exceptions for fats (the score for saturated fats is instead based on the ratio of saturated fats to total fat) and in certain situations for cheese.

A full description of how NutriScore is calculated is available through various resources online through the web search 'nutriscore calculation', as well on France's public health website (11).

When comparing the nutrition impacts of a diverse set of foods, we converted NutriScores A-E scale into a 1-5 scale to allow for averaging across products. When comparing the nutrition impacts of similar products (e.g. as with sausages, pesto, lasagna, and cookies), we instead converted the numeric value underlying NutriScore's A-E ranking into a score that ranges from 0 to 100. We did this by adding 15 to the score of each product (so that the score now ranges from 0 to 55), then dividing by 55, and then multiplying by 100.

Aggregate Categories Used in Figure 3:
In **Figures 4 and 5**, Aisles that contained similar products were aggregated for visibility and clarity when plotting. These aggregations are: "Frozen Meat Alternatives" and "Fresh Meat Alternatives" were condensed to "Meat Alternatives"; "Fresh Meat and Poultry", "Frozen Meat and Poultry", and "Cooked Meats, Sandwich Fillers & Deli" were to "Meats"; "Fresh Vegetables" and "Frozen Vegetables" were condensed to "Vegetables"; and "Frozen Pizza & Garlic Bread", "Fresh Pizza", and "Pasta & Garlic Bread" were condensed to "Pizza and Garlic

Bread”. Note that these aggregations were not conducted for the regression analyses examining the correlations between the environmental impact and nutrition impact of retail Aisles.

In addition, the name of several Aisles was shortened for clarity for plotting in Figures **4 and 5**. These include: “Dried Pasta, Rice, Noodles & Cous Cous” was renamed to “Dried Cereal Grains”; ‘Fresh Soup, Sandwiches & Salad Pots” was renamed to “Soup, Sandwiches & Salad Pots”; “Frozen Party Food & Sausage Rolls” was renamed to “Sausage Rolls & Party Food”; “Crisps, Snacks & Popcorn” was renamed to “Popcorn, Crisps & Snacks”; “Frozen Desserts, Ice Cream & Ice Lollies” was renamed to “Frozen Desserts, Ice Cream, and Lollies”; “Dried Fruit, Nuts, Nutrient Powders & Seeds” was renamed to “Nuts, Dried Fruit & Nutrient Powders”; “Frozen Yorkshire Puddings and Stuffing” was renamed to “Yorkshire Puddings”; “Jams, Sweet & Savoury Spreads” was renamed to “Sweet & Savoury Spreads”; “Cakes, Cake Bars, Slices & Pies” was renamed to “Cakes and Pies”; “Frozen Chips, Onion Rings, Potatoes & Rice” was renamed to “Roasted Potatoes, Chips, Onion Rings, & Rice”; “Fresh Fruit” was renamed to “Nuts and Fresh Fruit” (28% of products in the Aisle were nut products); “Frozen World Foods & Halal” was renamed to “World Foods & Halal”; “Counters” was renamed to “Deli Meat & Cheese”. In addition, to show differentiation between different types of meat, Shelves containing only ruminant meats (beef, sheep, and goat), were categorized into their own Aisle named “Beef and Lamb”.

Identifying Pesto Sauces, Lasagnas, and Sausages:
These products were identified using search terms for the Shelf they are categorized in, and for the names of the product. All searches were performed using the R function grepl(), and ignored capitalization. These searches in total identified 503 sausages, 161 pesto sauces, 413 cookies, and 107 lasagne.

Pesto Sauces:
The search term “pesto” was on product names to identify products for potential inclusion in the analysis.

The search term "dough|base|sauce|yeast|allinson's|mix|baguette|flour|bread sticks|hot & spicy chicken|fried chicken g|arancini bites|mozzarella sticks|potato wedges|john crabbie's|drink|lemonade|appletiser|coleslaw|dip|elderflower|\\bcoke\\b|coca-cola|zero sugar|\\bcola\\b|steak pie|mushroom pie|cheese burger|garlic slices|pizza bread|chicken bites|cooked chips|orangeade|chicken goujons|white rolls|garlic puree|tortelloni|lasagne|cappelletti|penne 300g|gnocchi|pennetwinpack|fusilli|tagliatelle|ravioli|ciabatta|linguine|spaghetti|linguine|breadsticks|seeded garlic flatbread|garlic tear & share|mezzelune|garlic bread|hot dog|garlic flatbread|garlic rustic wheel|flatbread|soup|vegetables & grains|bolognese|beef lasagne|macaroni cheese|fettucini|moussaka|risotta|pasta bake|tagliatelle|canneloni|penne|sicilian veg one pot|mushroom carbonara|tart frozen|tart flambee|baking tray|daal|Broccoletti Mezzelune|Mezzelune|piri piri|grated hard cheese|canelloni|canneloni|cannelloni|Finest Lamb, Rosemary & Garlic|whirls|lattice|grains|panini|fresh ideas|chicken|escalopes|pizza|bruschetta|bites|houmous|antipasti|chicken tray bake|risotto|salad|pesto butter|quiche|swirls|parmesan pasta|chicken pesto pasta|pesto escalope|tortelloni|quiche|tray bake|salad|sandwich|melts|roasting tray|chicken fillet|pasta with spinach|dressing|british lamb|vegetarian mozzarella|chicken pesto breast|semi dried tomato|salmon fillet|whirl|palmier|white wine mustard|focaccia|pasta with|chicken with|Tortelloni|breasts with|quinoa|Focaccia|spinach pasta|grissini" was then used on product names to disqualify products from inclusion in the analysis.

Lasagnas:
The search term “lasagne|lasagna” was used on product names to identify products for potential inclusion in the analysis.

The search term "barilla|noodle|sauce|sheet|mix for lasagne|recipe mix|lasagne mix|lasagna mix|meal kit" was then used on product names to disqualify products from inclusion in the analysis.

Sausages:
The search term “sausage” was used on product names to identify products for potential inclusion in the analysis.

The search term “baked Bean|spaghetti|pasta|roll|mash|triple|casserole|turkey breast stuffed with|muffin|egg|pizza|kettle & apple|tortelloni|tortellini|heinz|soup|stew” was then used on product names to disqualify products from inclusion in the analysis.

Cookies:
Cookies were predominantly identified based on the Aisle and Shelf into which they were classified and their product names. We limited this search to sweet cookies.

We identified cookies as products in the Aisles listed below that also contained either ‘cookie’ or ‘biscuit’ in their name but that were not identified as seasonal products (a name containing e.g. Halloween, Christmas, etc), or that contained cheese in the product.

The Aisles searched to identify cookies are as follows:

'Bakery Counter'; 'Biscuits & Cereal Bars'; 'Bakery'; 'Bakery Free From'; 'Baking, Desserts & Spreads'; 'Biscuits'; 'Biscuits & Chocolate'; 'Cookies & Biscuits'; 'Doughnuts, Muffins & Cookies'; 'Desserts & pastry'; 'Free From'; 'Free From Range'; 'Free From Bakery'; 'Freefrom'; and 'From our Bakery'

Additional analyses on validating the algorithm’s accuracy:
We additionally investigated the accuracy of the algorithm at estimating the composition of different ingredients in food products (**Figures S9-11, Tables S4-5**). In doing so, we compared the absolute difference between the estimated and known percent composition (calculated as estimated composition – known composition).

These analyses show the algorithm is able to consistently estimate the composition of different ingredients. Comparing the estimated and known composition across all ingredients reveals no significant difference (paired t-test; P-value = 0.949; df = 1,842,369). Controlling for ingredient order shows the algorithm slightly overestimates the composition of the first and second ingredient (by an absolute difference of 1.29% and 0.45%, of the product’s total composition, respectively; paired t-tests, P-value < 0.001 for both comparisons), and slightly underestimates the composition of most remaining ingredients (**Table S4**).

Looking across environmental database food categories likewise shows the algorithm is able to consistently estimate the composition of different types of ingredients. Across the food categories, the algorithm estimated the composition of ingredients within 1% of the known composition for 45 of the 48 food categories (91.7% of categories), within 5% for 45 of the categories (93.8%), and within 10% for all 48 of the categories. It was least accurate for pigmeat (mean difference of 8.0% of the product’s total composition), palm oil (mean difference of 7.2%),

olives (mean difference of 6.9%), bovine meat from beef herds (mean difference of 6.4%), and rapeseed oil (mean difference of 4.3%). See **Table S5** for accuracy by environmental food category.

The results are similar when assuming a worst case scenario in which the composition of no ingredient in a product is known (**Figures S9-11**). In this situation, there is no significant difference between the estimated and known percent composition across all ingredients (paired t-test; P-value = 0.952; df = 44,354). Looking across ingredient order, the estimated composition is within 1% of the known composition for every ingredient location, with the exception of the 2nd ingredient in a product (where the difference is 1.71%) (**Tables S4-5**). In this worst case scenario, the algorithm estimated the composition of ingredients within 1% of the known composition for 35 of 48 food categories (72.9% of categories), within 5% for 43 of 448 categories (89.6%), and within 10% for 45 of the 48 categories (93.8%). The least accurate categories in this worst case scenario were olives (17.1% difference), pig meat (16.0% difference), tea (12.6% difference), palm oil (12.2% difference), and rapeseed oil (10.2% difference).

Sensitivity on how sourcing can effect the environmental impacts of a product:
We examined how uncertainty in ingredient sourcing can impact a product's total environmental impact. To do this, during the Monte Carlo analysis, we sampled the Monte Carlo iterations that equated with the 5th, 10th, 25th, 50th, 75th, 90th, and 95th percentile impacts for each given product.

We then compared the differences in impacts for these percentiles. Specifically, we examined the relative differences (calculated as the nth percentile impact divided by the 50th percentile impact) as well as absolute differences (calculated as the nth percentile impact minus the 50th percentile impact).

The results of these analyses are shown in **Figures S13**. On average across all products at Tesco, the 50th percentile impact was: 87% higher than the 5th percentile impact (an average absolute difference of 0.13kg CO2e and 0.14m^2 of land; median differences of 0.03kg CO_2e and 0.04m^2 of land); 65% higher than the 10th percentile impact (an average absolute difference of 0.11kg CO2e and 0.13m^2 of land; median differences of 0.02kg CO_2e and 0.04m^2 of land); 33% higher than the 25th percentile impact (an average absolute difference of 0.06kg CO2e and 0.09m^2 of land; median differences of 0.01kg CO_2e and 0.01m^2 of land); was 58% the impact of the 75th percentile impact (an average absolute difference of 0.09kg CO2e and 0.11m^2 of land; median differences of <0.01kg CO_2e and <0.01m^2 of land); 33% the impact of the 90th percentile impact (an average absolute difference of 0.40kg CO2e and 0.36m^2 of land; median differences of <0.01kg CO_2e and <0.01m^2 of land); and 25% of the 95th percentile impact (an average absolute difference of 0.69kg CO2e and 0.69m^2 of land; median differences of 0.02kg CO_2e and 0.06m^2 of land).

When comparing across extremes, we find that the 95th percentile impact is, on average, 826% higher than the 5th percentile impact for the same product (an absolute difference of 0.82kg CO2e and 0.83m^2 of land). However, these results are highly right-skewed. For 50% of products, the 95th percentile impact is less than 510% greater than the 5th percentile impact. When looking at absolute differences, the median difference between the 95th and 5th percentile impact was 0.06kg CO_2e and 0.10m^2 or less per 100g. However, in extreme cases (at the 95th quantile or above), the relative difference increases to a 2,450% difference while the absolute difference increases to 4.80kg CO_2e and 2.53m^2 land.

The products with the largest relative differences between the 5th and 95th percentile impacts are a combination of fish (due to sourcing between fisheries and aquaculture), tree nuts (which can

have highly variable water use), and products that have an estimated environmental impact score that is 2 or below. The largest absolute differences are dominated by chocolate, coffee, and hard cheese (e.g. parmesan, etc) for greenhouse gas emissions, and by beef, hard cheese, and coffee for land.

In total, this provides further evidence that, for most products, lack of sourcing information may not have a large effect on the overall estimated environmental impact score for most products or on the absolute environmental impacts for individual environmental indicators. However, because in certain situations uncertainty in sourcing can result in a large uncertainty in the product's total environmental impact score, as well as in the absolute impact for individual environmental indicators, more transparency in ingredient sourcing for different products is needed to derive more accurate estimates of the environmental impacts of different food products.

Sensitivity analyses on sourcing for sausages, lasagna, pesto sauces, and cookies:
We further conducted sensitivity analyses on the four specific food product types examined in this analysis (**Figure S19**).

For sausages, the differences between meat-based and non-meat based sausages remain significant until extreme assumptions in sourcing. More specifically, the difference in environmental impacts between ruminant-based and pork-based sausages and non-meat sausages remain significant even if ingredients in the meat-based sausages are sourced from the combination of production systems that equates to the product having the 10th% observed impact (as derived from the 1,000 iterations of the Monte Carlo analysis), while non-meat based sausages are sourced from the combination of production systems that equates to the product having the 90th% observed impact (all comparisons using Tukey's HSD test; P-value < 0.05). For ruminant-based sausages, this difference remains even under the extreme circumstance where ruminant sausages have impacts equivalent to the 5th% observed impact and non-meat based sausages have impacts equivalent to the 95th% observed impact (Tukey's HSD test; P-value < 0.001). For pork-based sausages, however, in this extreme situation vegan sausages have a significantly higher impact than pork-based sausages (Tukey's HSD test; P-value = 0.01), while vegetarian sausages have a similar impact to pork-based sausages (Tukey's HSD test; P-value = 0.86).

For lasagne, only when we assumed meat-based products were sourced from the combination of production systems that equates to the product having the 10th% impact (as derived from the Monte Carlo analysis) and the non-meat products having impacts equivalent to the 90th% impact did the difference in environmental impacts between meat-based and non-meat based products become non-significant (all comparisons using Tukey's HSD test; P-value > 0.05). The difference in impacts remained significant when we instead assumed meat-based products and non-meat based products were sourced from production systems that equated to the 25th% and 75th% impacts, respectively (Tukey's HSD test; P-value < 0.05 for all comparisons). Meat-based lasagne also had significantly higher environmental impacts when meat-based products were sourced from highly efficient production systems (equating to the 5th% impact) and non-meat lasagne were sourced from average production systems (Tukey's HSD; P-value < 0.05 for all comparisons). When instead non-meat lasagna was sourced from highly inefficient production systems (equating to the 95th% impact) and meat-lasagne were sourced from average production systems, non-meat lasagne had significantly lower environmental impacts than lasagna containing ruminant meat, but similar impacts to lasagna containing pork (Tukey's HSD; P-value < 0.05 for all comparisons). Note that all above comparisons with lasagna containing poultry meat were insignificant due to the small number of products containing poultry meat (n = 2).

For pesto sauces, the difference in environmental impacts between nut-containing and non-nut pestos flipped under mild assumptions in ingredient sourcing. When we assumed that nut-containing pestos were sourced from relatively efficient production systems (those that had impacts equivalent to the 25th% observed impact) whereas non-nut pestos were sourced from relatively inefficient production systems (those that had impacts equivalent to the 75th% impact), nut-containing pestos instead had significantly lower environmental impacts than did non-nut pestos independent of whether the pesto also contained dairy (Tukey's HSD; P-value < 0.05 for all comparisons).

For cookies, the difference in environmental impacts between chocolate and non-choclate cookies also flipped under mild assumptions in ingredient sourcing. When we assumed chocolate cookies were sourced from relatively efficient production systems (equivalent to the 25th% impact from the Monte Carlo analysis) and non-chocolate cookies were sourced from relative inefficient production systems (equivalent to the 75th% impact from the Monte Carlo analysis), chocolate cookies instead had lower environmental impacts than did non-chocolate cookies (paired t-test, P-value = 0.049). This difference was also observed when non-chocolate cookies were sourced from some of the least sustainable producers (equivalent to the 95th% impacts) and chocolate cookies were sourced from average production systems (paired t-test, P-value < 0.001), or alternatively when chocolate cookies were sourced from more sustainable producers (equivalent ot the 95th% impact) and non-chocolate cookies were sourced from average production systems (paired t-test, P-value < 0.001).

Supplemental Figures

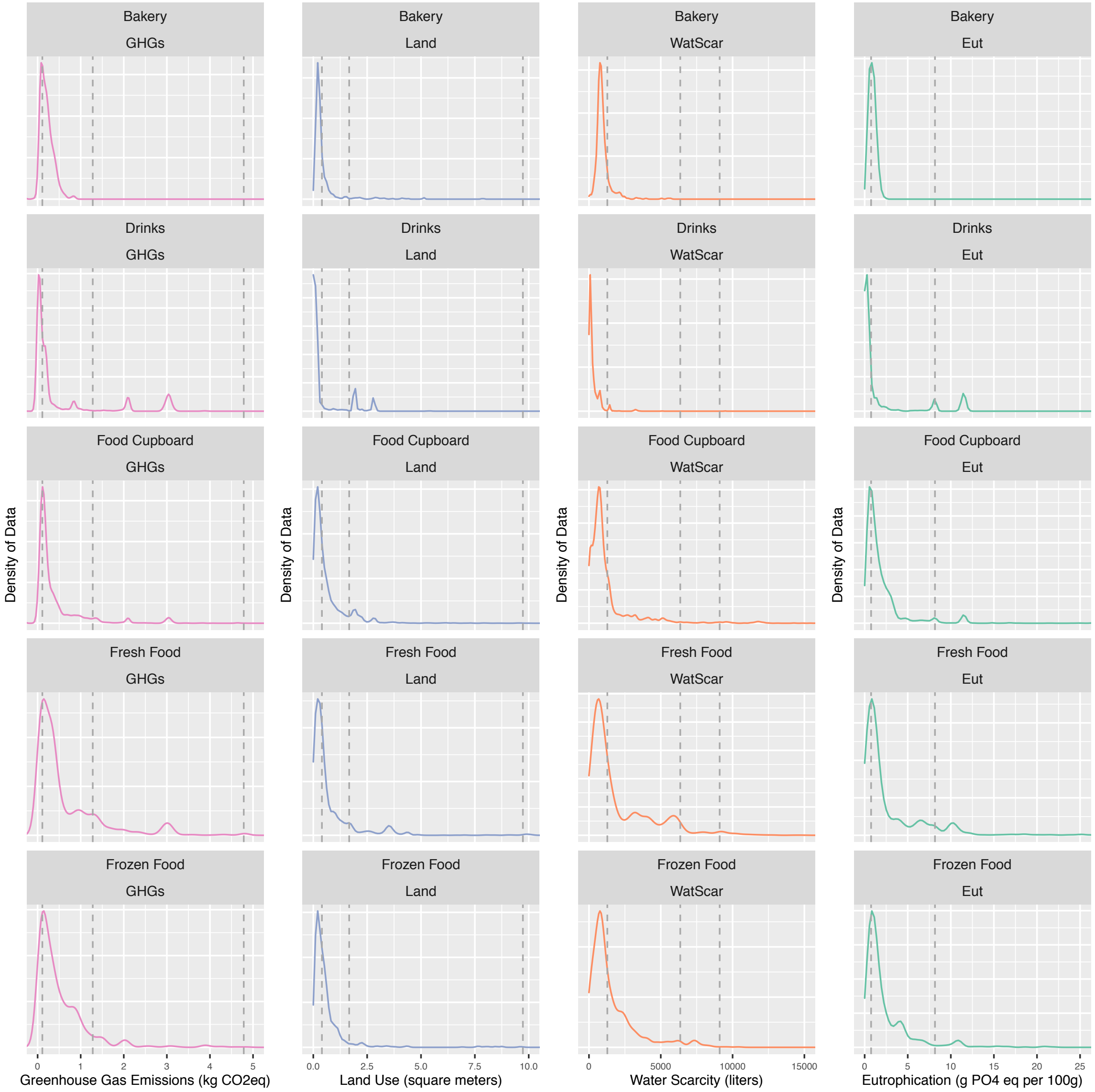

Fig. S1. **Distribution of environmental impacts per 100g for each environmental indicator of products in different Tesco Departments.** Vertical dashed lines indicate, from left to right, the mean impact of wheat, pig meat, and beef (from a dairy herd).

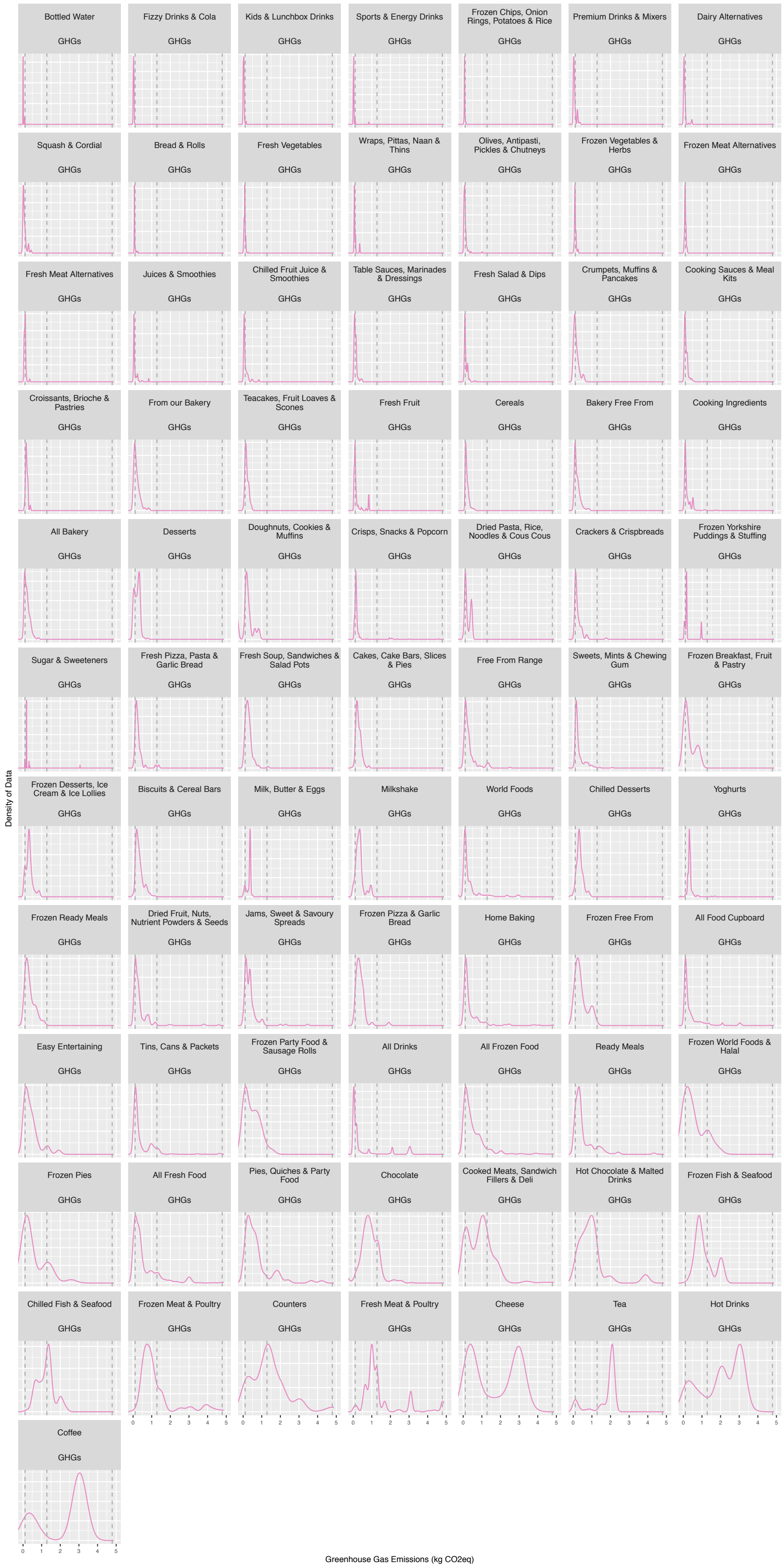
Bottled Water
Fizzy Drinks & Cola
Kids & Lunchbox Drinks
Sports & Energy Drinks
Frozen Chips, Onion Rings, Potatoes & Rice
Premium Drinks & Mixers
Dairy Alternatives
Squash & Cordial
Bread & Rolls
Fresh Vegetables
Wraps, Pittas, Naan & Thins
Olives, Antipasti, Pickles & Chutneys
Frozen Vegetables & Herbs
Frozen Meat Alternatives
Fresh Meat Alternatives
Juices & Smoothies
Chilled Fruit Juice & Smoothies
Table Sauces, Marinades & Dressings
Fresh Salad & Dips
Crumpets, Muffins & Pancakes
Cooking Sauces & Meal Kits
Croissants, Brioche & Pastries
From our Bakery
Teacakes, Fruit Loaves & Scones
Fresh Fruit
Cereals
Bakery Free From
Cooking Ingredients
All Bakery
Desserts
Doughnuts, Cookies & Muffins
Crisps, Snacks & Popcorn
Dried Pasta, Rice, Noodles & Cous Cous
Crackers & Crispbreads
Frozen Yorkshire Puddings & Stuffing
Sugar & Sweeteners
Fresh Pizza, Pasta & Garlic Bread
Fresh Soup, Sandwiches & Salad Pots
Cakes, Cake Bars, Slices & Pies
Free From Range
Sweets, Mints & Chewing Gum
Frozen Breakfast, Fruit & Pastry
Frozen Desserts, Ice Cream & Ice Lollies
Biscuits & Cereal Bars
Milk, Butter & Eggs
Milkshake
World Foods
Chilled Desserts
Yoghurts
Frozen Ready Meals
Dried Fruit, Nuts, Nutrient Powders & Seeds
Jams, Sweet & Savoury Spreads
Frozen Pizza & Garlic Bread
Home Baking
Frozen Free From
All Food Cupboard
Easy Entertaining
Tins, Cans & Packets
Frozen Party Food & Sausage Rolls
All Drinks
All Frozen Food
Ready Meals
Frozen World Foods & Halal
Frozen Pies
All Fresh Food
Pies, Quiches & Party Food
Chocolate
Cooked Meats, Sandwich Fillers & Deli
Hot Chocolate & Malted Drinks
Frozen Fish & Seafood
Chilled Fish & Seafood
Frozen Meat & Poultry
Counters
Fresh Meat & Poultry
Cheese
Tea
Hot Drinks
Coffee
GHGs
0 1 2 3 4 5
Density of Data
Greenhouse Gas Emissions (kg CO2eq)

Fig. S2. Distribution of greenhouse gas emissions per 100g of products in different Tesco Aisles. Vertical dashed lines indicate, from left to right, the mean impact of wheat, pig meat, and beef (from a dairy herd). Aisles are sorted from lowest to highest average greenhouse gas emissions.

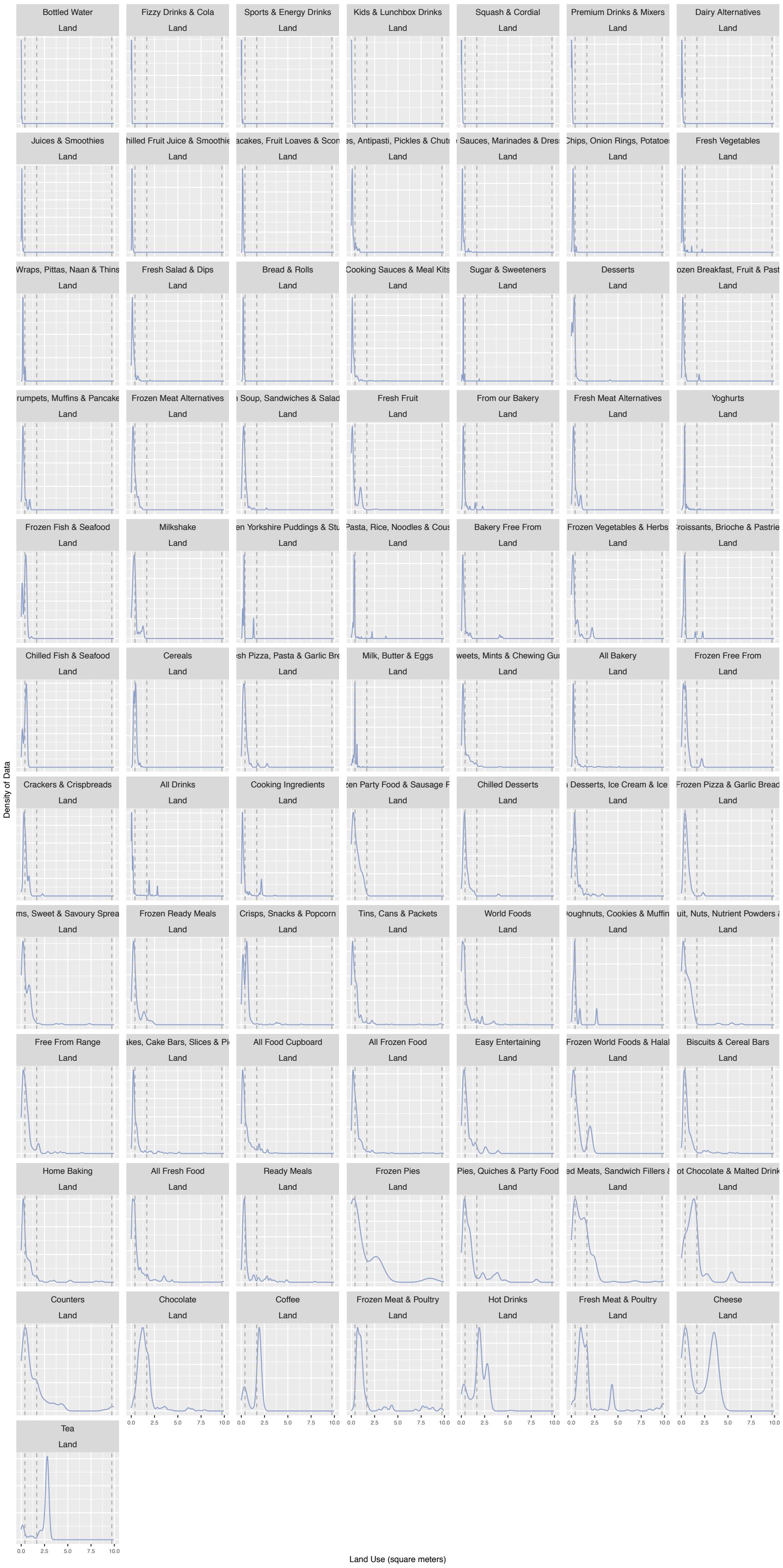
Bottled Water
Fizzy Drinks & Cola
Sports & Energy Drinks
Kids & Lunchbox Drinks
Squash & Cordial
Premium Drinks & Mixers
Dairy Alternatives
Juices & Smoothies
hilled Fruit Juice & Smoothie
acakes, Fruit Loaves & Scon
es, Antipasti, Pickles & Chutn
Sauces, Marinades & Dress
Chips, Onion Rings, Potatoes
Fresh Vegetables
Wraps, Pittas, Naan & Thins
Fresh Salad & Dips
Bread & Rolls
Cooking Sauces & Meal Kits
Sugar & Sweeteners
Desserts
ozen Breakfast, Fruit & Past
rumpets, Muffins & Pancake
Frozen Meat Alternatives
Soup, Sandwiches & Salad
Fresh Fruit
From our Bakery
Fresh Meat Alternatives
Yoghurts
Frozen Fish & Seafood
Milkshake
en Yorkshire Puddings & Stu
Pasta, Rice, Noodles & Cous
Bakery Free From
Frozen Vegetables & Herbs
roissants, Brioche & Pastrie
Chilled Fish & Seafood
Cereals
sh Pizza, Pasta & Garlic Bre
Milk, Butter & Eggs
weets, Mints & Chewing Gu
All Bakery
Frozen Free From
Crackers & Crispbreads
All Drinks
Cooking Ingredients
zen Party Food & Sausage R
Chilled Desserts
Desserts, Ice Cream & Ice
Frozen Pizza & Garlic Bread
ms, Sweet & Savoury Sprea
Frozen Ready Meals
Crisps, Snacks & Popcorn
Tins, Cans & Packets
World Foods
oughnuts, Cookies & Muffin
uit, Nuts, Nutrient Powders &
Free From Range
akes, Cake Bars, Slices & Pi
All Food Cupboard
All Frozen Food
Easy Entertaining
Frozen World Foods & Halal
Biscuits & Cereal Bars
Home Baking
All Fresh Food
Ready Meals
Frozen Pies
Pies, Quiches & Party Food
ed Meats, Sandwich Fillers &
ot Chocolate & Malted Drink
Counters
Chocolate
Coffee
Frozen Meat & Poultry
Hot Drinks
Fresh Meat & Poultry
Cheese
Tea
Land
0.0
2.5
5.0
7.5
10.0
Density of Data
Land Use (square meters)

Fig. S3. Distribution of land per 100g of products in different Tesco Aisles. Vertical dashed lines indicate, from left to right, the mean impact of wheat, pig meat, and beef (from a dairy herd). Aisles are sorted from lowest to highest average land use.

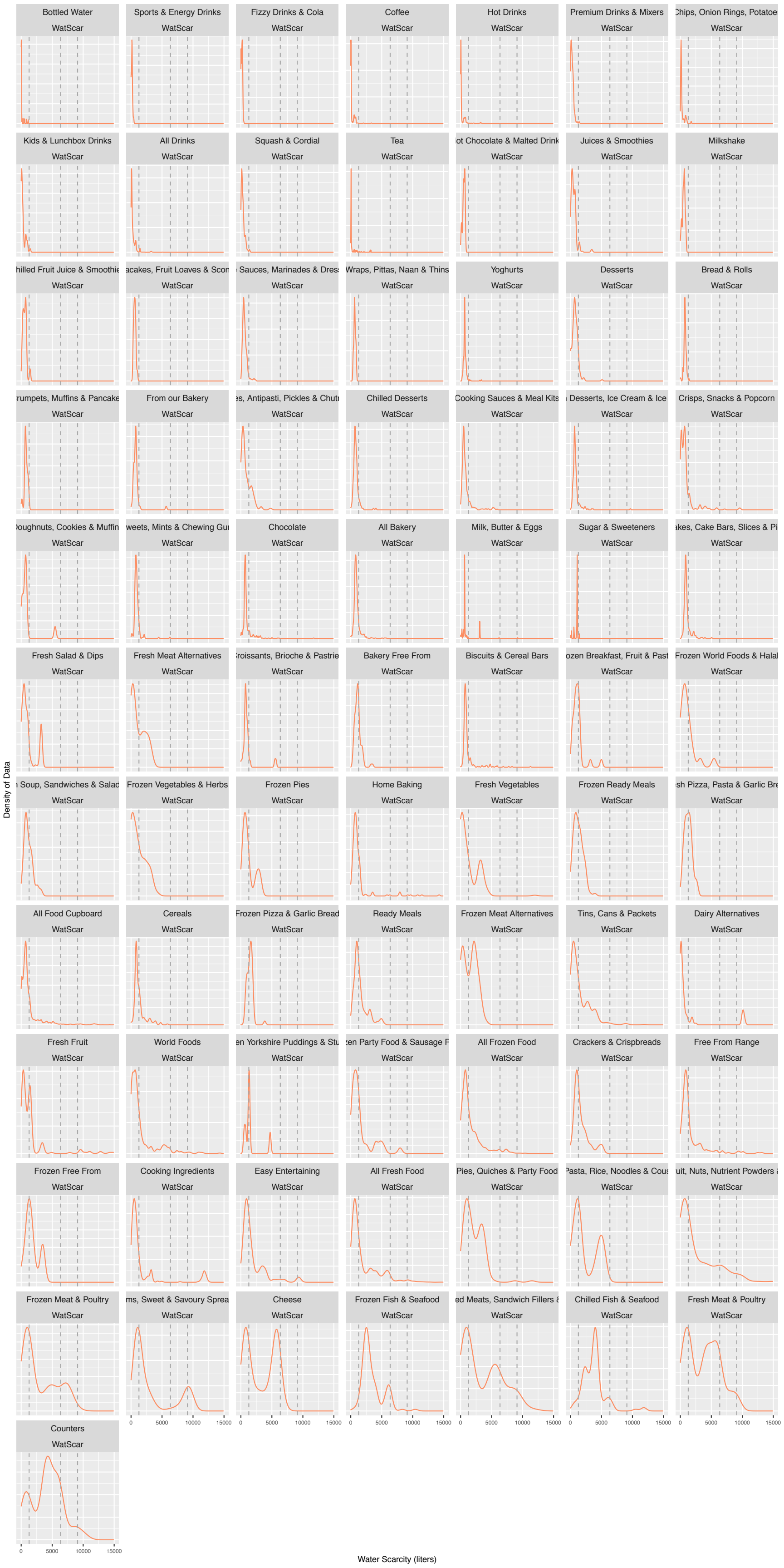
Bottled Water
Sports & Energy Drinks
Fizzy Drinks & Cola
Coffee
Hot Drinks
Premium Drinks & Mixers
Kids & Lunchbox Drinks
All Drinks
Squash & Cordial
Tea
Juices & Smoothies
Milkshake
Yoghurts
Desserts
Bread & Rolls
From our Bakery
Chilled Desserts
Cooking Sauces & Meal Kits
Crisps, Snacks & Popcorn
Chocolate
All Bakery
Milk, Butter & Eggs
Sugar & Sweeteners
Fresh Salad & Dips
Fresh Meat Alternatives
Bakery Free From
Biscuits & Cereal Bars
Frozen World Foods & Halal
Frozen Vegetables & Herbs
Frozen Pies
Home Baking
Fresh Vegetables
Frozen Ready Meals
All Food Cupboard
Cereals
Frozen Pizza & Garlic Bread
Ready Meals
Frozen Meat Alternatives
Tins, Cans & Packets
Dairy Alternatives
Fresh Fruit
World Foods
All Frozen Food
Crackers & Crispbreads
Free From Range
Frozen Free From
Cooking Ingredients
Easy Entertaining
All Fresh Food
Pies, Quiches & Party Food
Frozen Meat & Poultry
Cheese
Frozen Fish & Seafood
Chilled Fish & Seafood
Fresh Meat & Poultry
Counters
WatScar
Density of Data
Water Scarcity (liters)
0
5000
10000
15000

Fig. S4. Distribution of scarcity weighted water use per 100g of products in different Tesco Aisles. Vertical dashed lines indicate, from left to right, the mean impact of wheat, pig meat, and beef (from a dairy herd). Aisles are sorted from lowest to highest average water use.

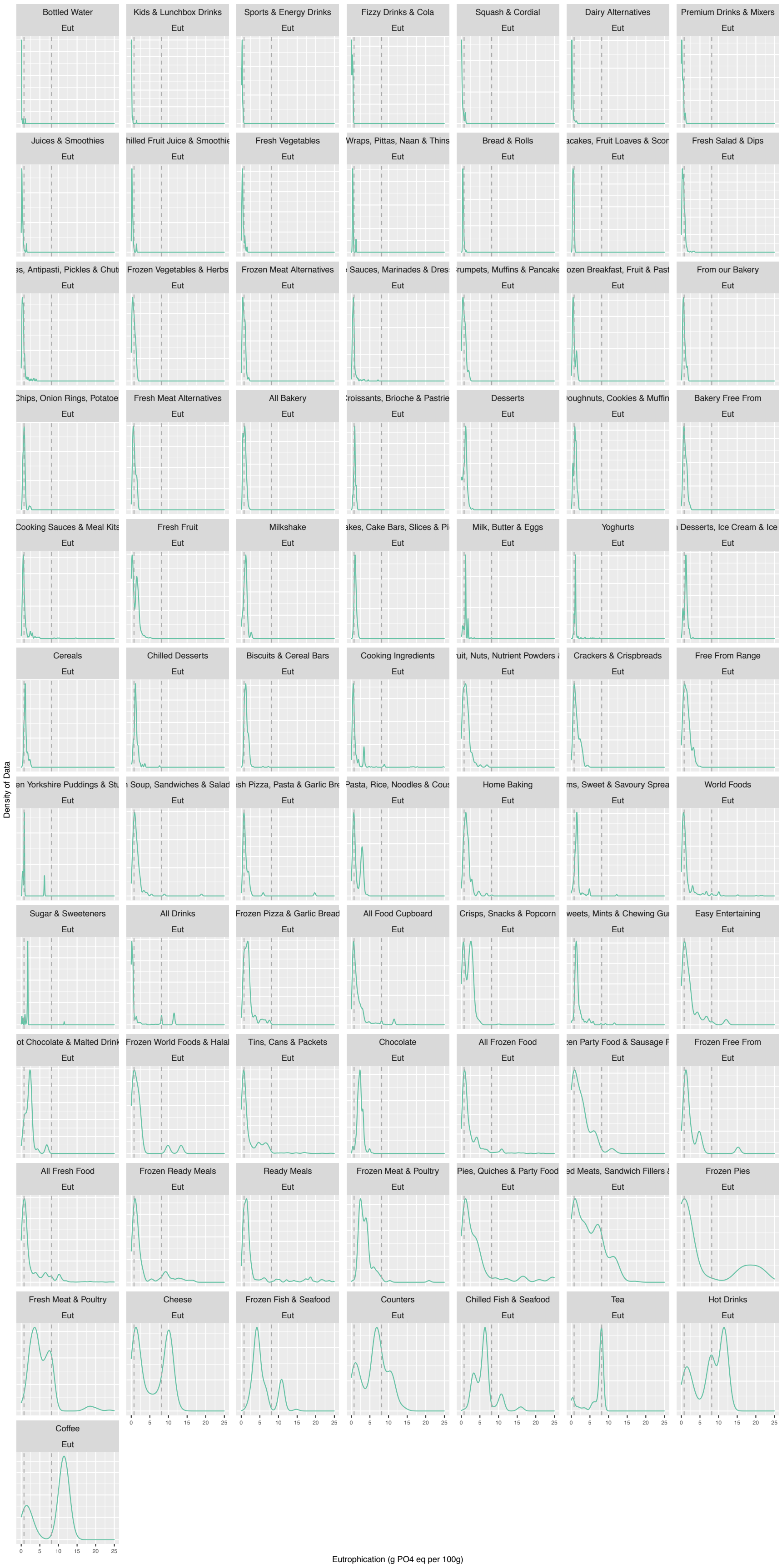
Bottled Water
Kids & Lunchbox Drinks
Sports & Energy Drinks
Fizzy Drinks & Cola
Squash & Cordial
Dairy Alternatives
Premium Drinks & Mixers
Juices & Smoothies
Fresh Vegetables
Bread & Rolls
Fresh Salad & Dips
Frozen Vegetables & Herbs
Frozen Meat Alternatives
From our Bakery
Fresh Meat Alternatives
All Bakery
Desserts
Bakery Free From
Cooking Sauces & Meal Kits
Fresh Fruit
Milkshake
Milk, Butter & Eggs
Yoghurts
Cereals
Chilled Desserts
Biscuits & Cereal Bars
Cooking Ingredients
Crackers & Crispbreads
Free From Range
Home Baking
World Foods
Sugar & Sweeteners
All Drinks
Frozen Pizza & Garlic Bread
All Food Cupboard
Crisps, Snacks & Popcorn
Easy Entertaining
Frozen World Foods & Halal
Tins, Cans & Packets
Chocolate
All Frozen Food
Frozen Free From
All Fresh Food
Frozen Ready Meals
Ready Meals
Frozen Meat & Poultry
Pies, Quiches & Party Food
Frozen Pies
Fresh Meat & Poultry
Cheese
Frozen Fish & Seafood
Counters
Chilled Fish & Seafood
Tea
Hot Drinks
Coffee
Eut
Density of Data
0 5 10 15 20 25
Eutrophication (g PO4 eq per 100g)

Fig. S5. Distribution of eutrophication potential per 100g of products in different Tesco Aisles. Vertical dashed lines indicate, from left to right, the mean impact of wheat, pig meat, and beef (from a dairy herd). Aisles are sorted from lowest to highest average eutrophication potential.

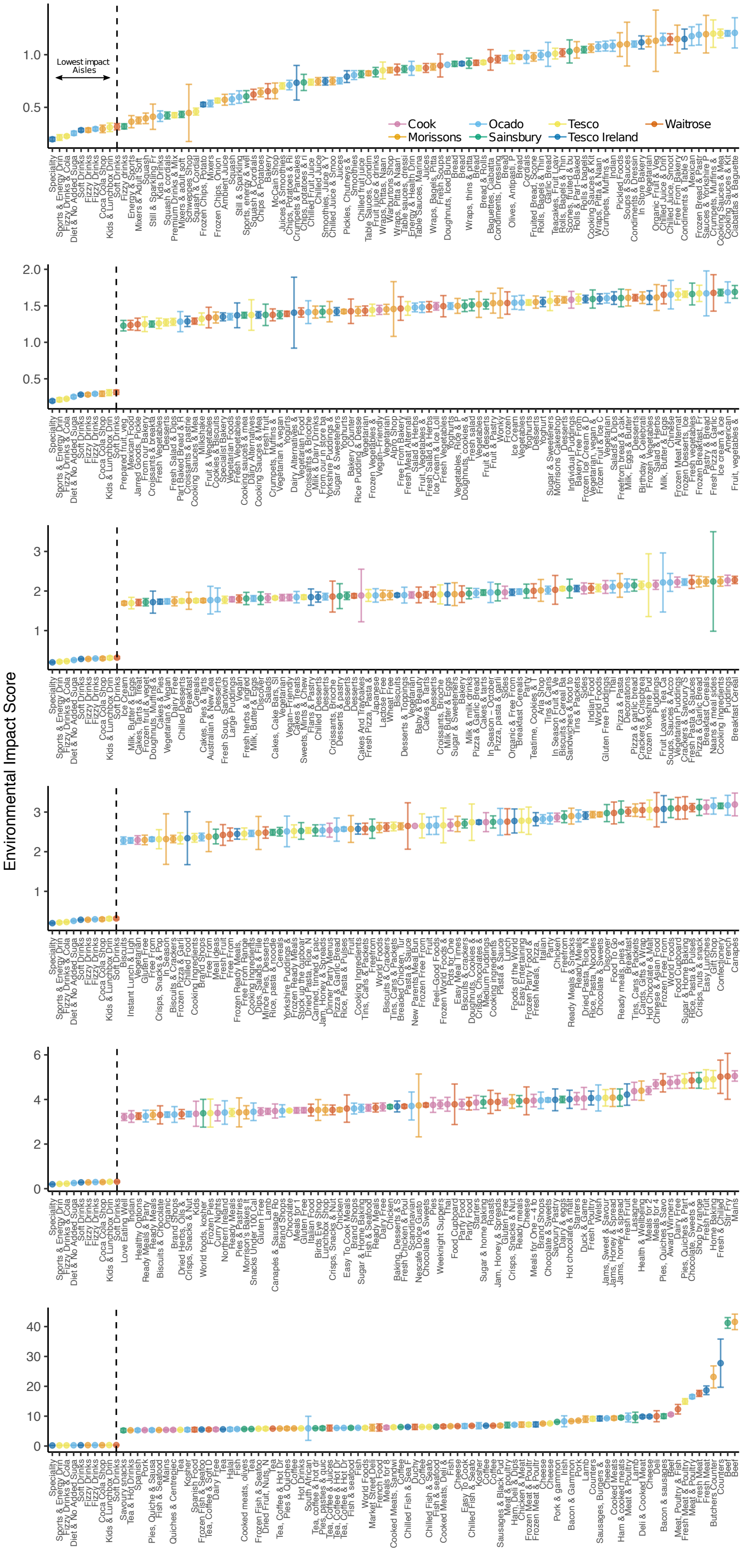

Environmental Impact Score
Lowest impact Aisles
Cook
Morissons
Ocado
Sainsbury
Tesco
Tesco Ireland
Waitrose

Figure S6. Environmental impact score of products in different retail Aisles. Points are coloured by retailer, show mean estimates of all products in the retailer Aisle. Error bars show mean +- 1 s.e.m. The Aisles left of the dotted vertical line are the 10 lowest impact Aisles.

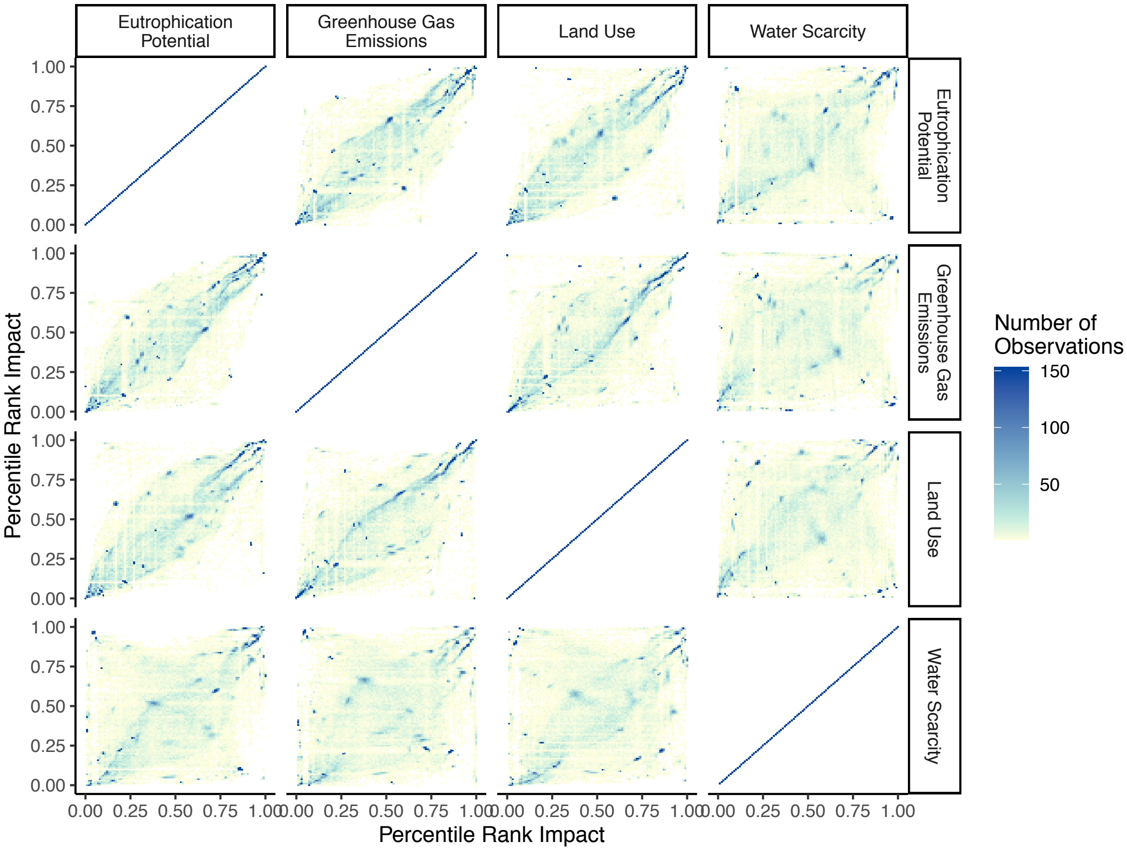

Fig. S7. Heat map showing pairwise comparisons between the percentile ranking of each product across indicators. Coloring indicates the number of products at a given coordinate, such that dark blues indicate higher representation and pale yellows indicate lower representation. Products with similar percentile impacts between indicators are near the diagonal line where y = x, whereas products with a high impact for one indicator and low impact for another are above or below the line where y = x. Spearman correlations between indicators are reported in **Table S1**.

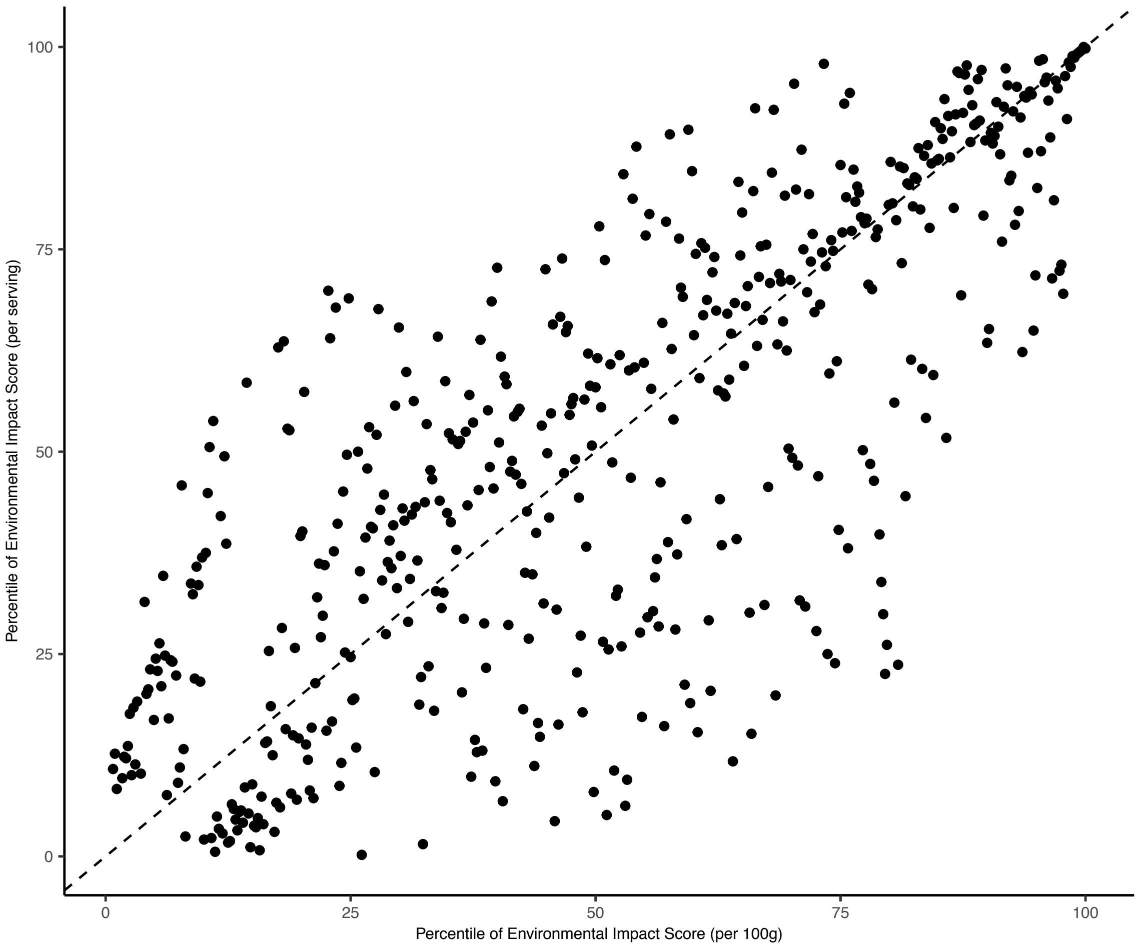

Fig. S8. **Comparison between environmental impact per 100g and per serving of product.** Each point indicates the average impacts of products in an Aisle, and the contains all Aisles across all food retailers examined. X-axis indicates the percentile of that Aisle's environmental impact when measured per 100g, whereas the y-axis indicates the percentile of that Aisle's environmental impact when measured per serving. Dashed line indicates the fit x = y.

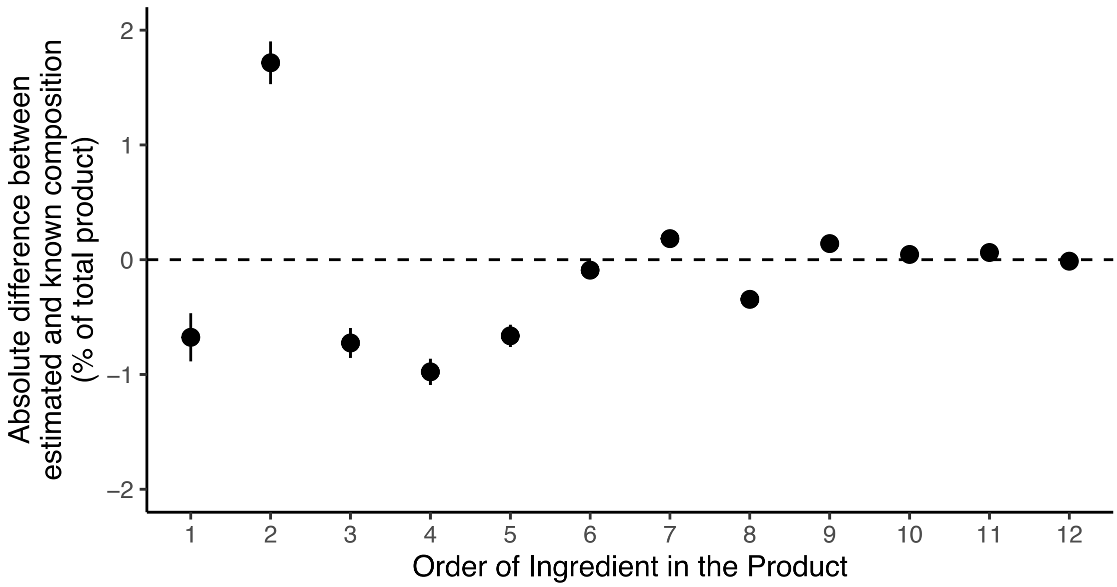

Figure S9. Accuracy of the algorithm in estimating the composition of ingredients in a product, based on the position of that ingredient in the product's ingredient list. Points indicate the mean difference, and error bars indicate +- 1.s.e.m. Positive values indicate the algorithm overestimates the abundance of the ingredient, whereas negative values indicate the algorithm underestimates the abundance of the ingredient. Plot shows data when assuming that the composition of no ingredients in the product was known.

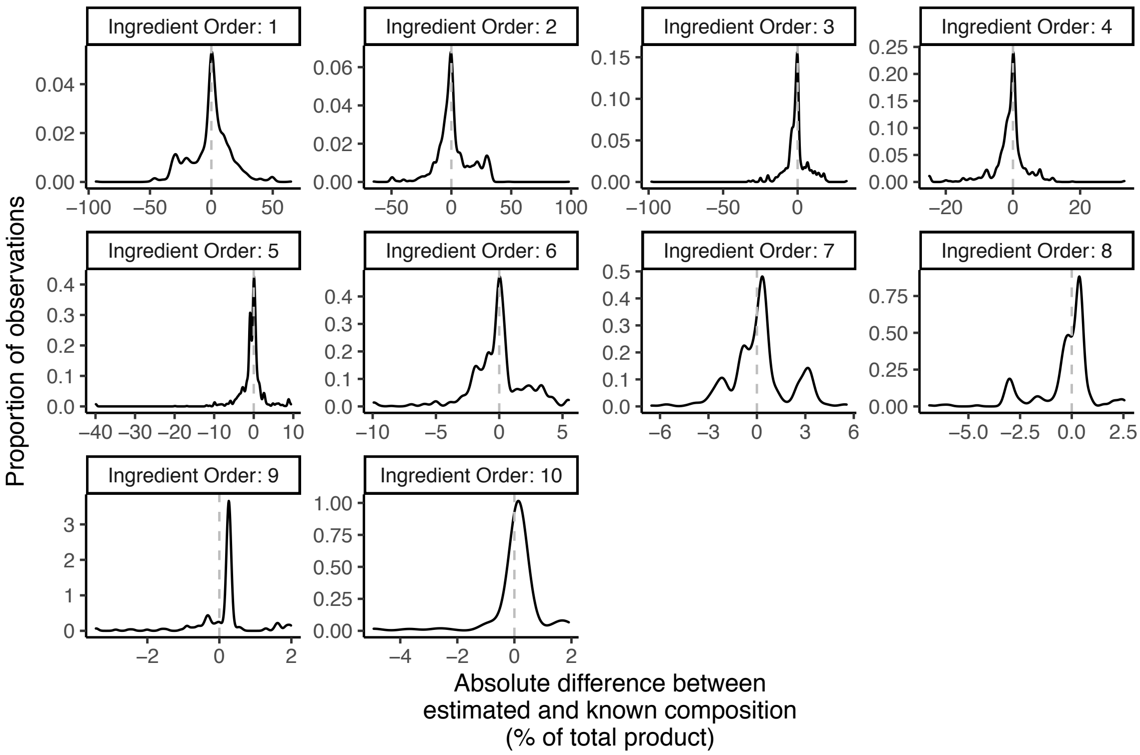

Figure S10. Density plots showing the accuracy of the algorithm in estimating the composition of ingredients in a product, based on the position of that ingredient in the product's ingredient list. Positive values on the x-axis indicate the algorithm overestimates the abundance of the ingredient, whereas negative values on the x-axis indicate the algorithm underestimates the abundance of the ingredient. Plot shows data when assuming that the composition of no ingredients in the product was known.

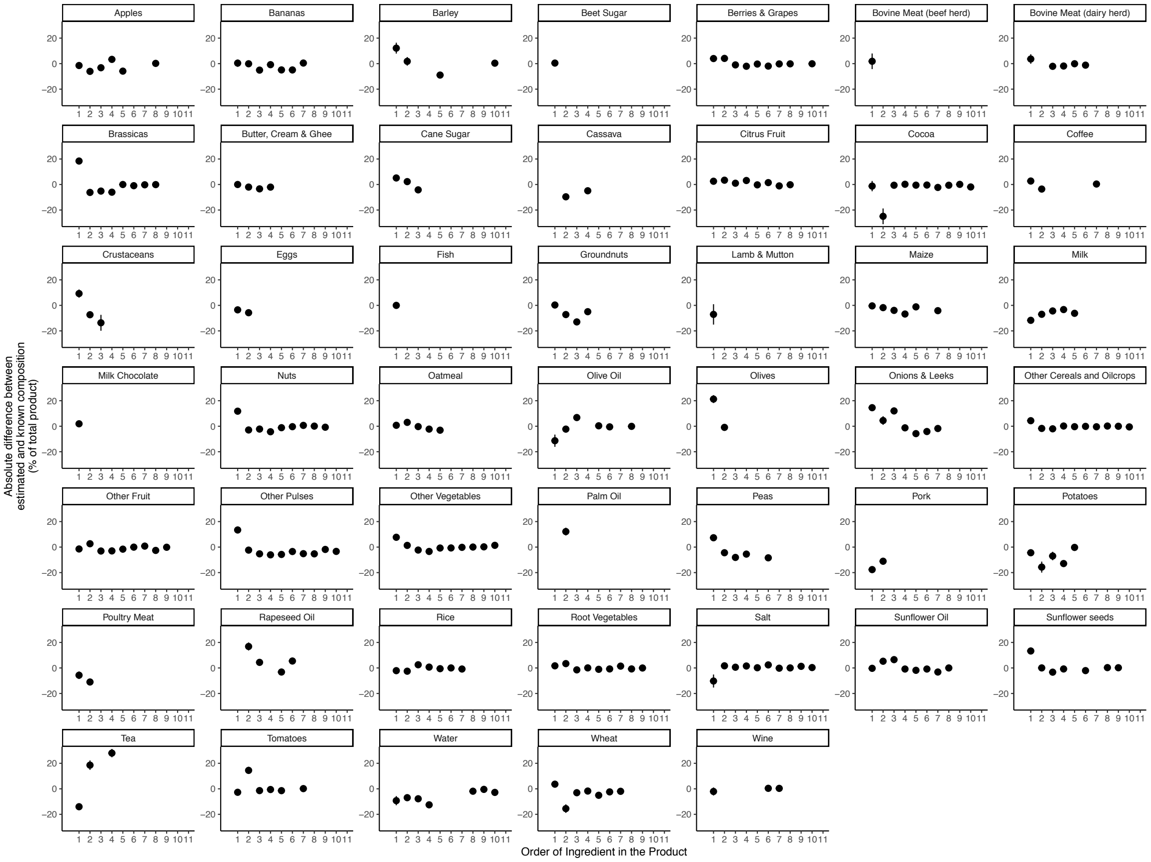

Figure S11. Accuracy of the algorithm in estimating the composition of ingredients by environmental database food category and order of ingredient in the product. Positive values on the y-axis indicate the algorithm overestimates the abundance of the ingredient, whereas negative values on the y-axis indicate the algorithm underestimates the abundance of the ingredient. Plot shows data when assuming that the composition of no ingredients in the product was known. Missing points indicate no observations of that combination of environmental database food category and order of ingredient.

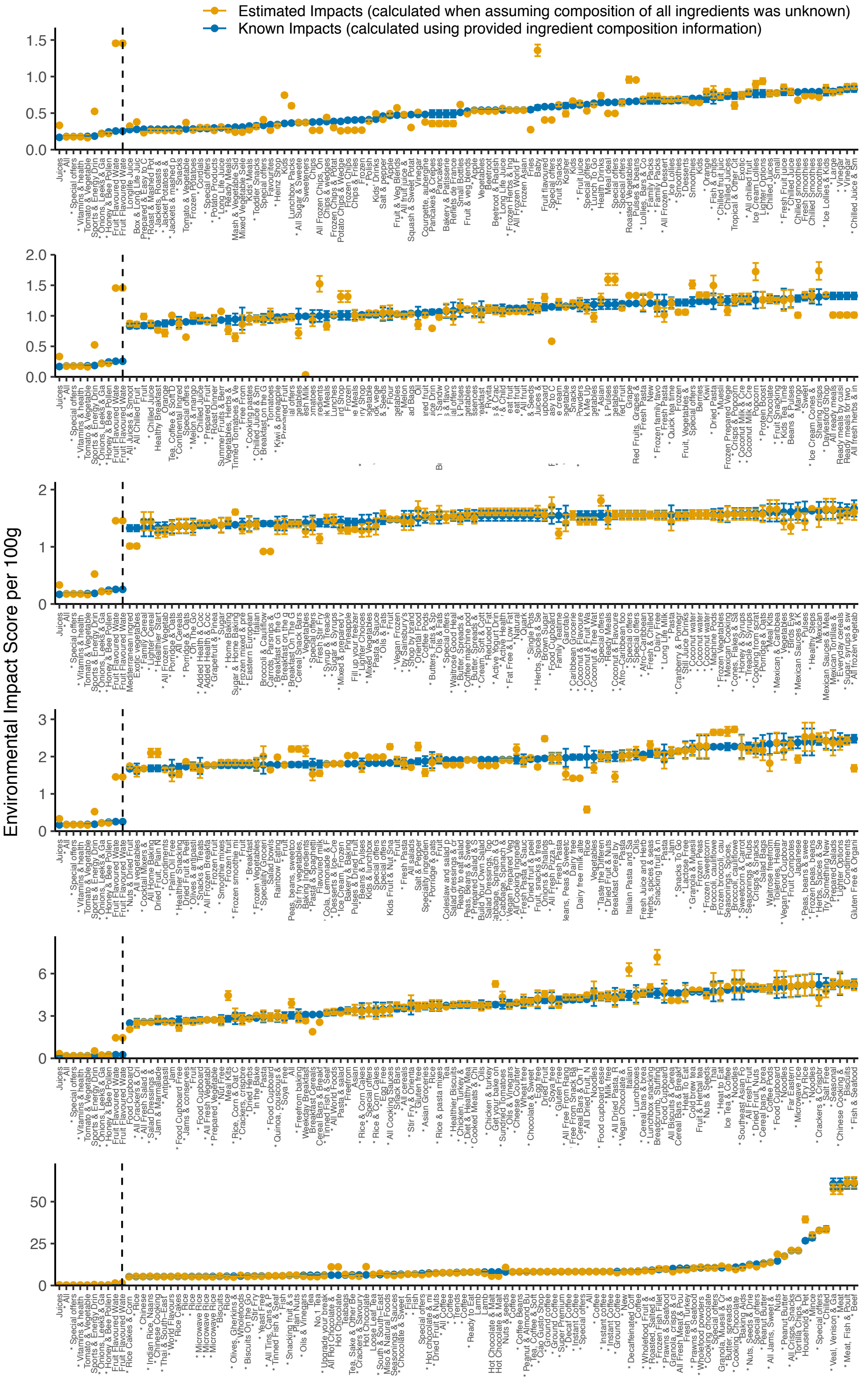
Estimated Impacts (calculated when assuming composition of all ingredients was unknown)
Known Impacts (calculated using provided ingredient composition information)
Environmental Impact Score per 100g

Fig. S12. Comparison between the known (blue) and estimated (golden) environmental impact scores of retail 'Shelves'. Data includes all products where the composition information of all ingredients in the product was available. Known environmental impacts were calculated using ingredient composition provided in the ingredients list, whereas the estimated environmental impacts were calculated when assuming the percent composition of all ingredients in the product were unknown. Products were sorted into Shelves using the categorization systems employed by each food retailer on their website. Shelves left of the vertical dashed line are the ten Shelves with the lowest average known environmental impact score, and are shown to allow comparison across panels. Error bars indicate mean +- one standard error. Shelf labels are limited to the first 20 characters of the Shelf name due to size limitations. * indicate whether the uncertainty around the known and estimated environmental impact scores overlap.

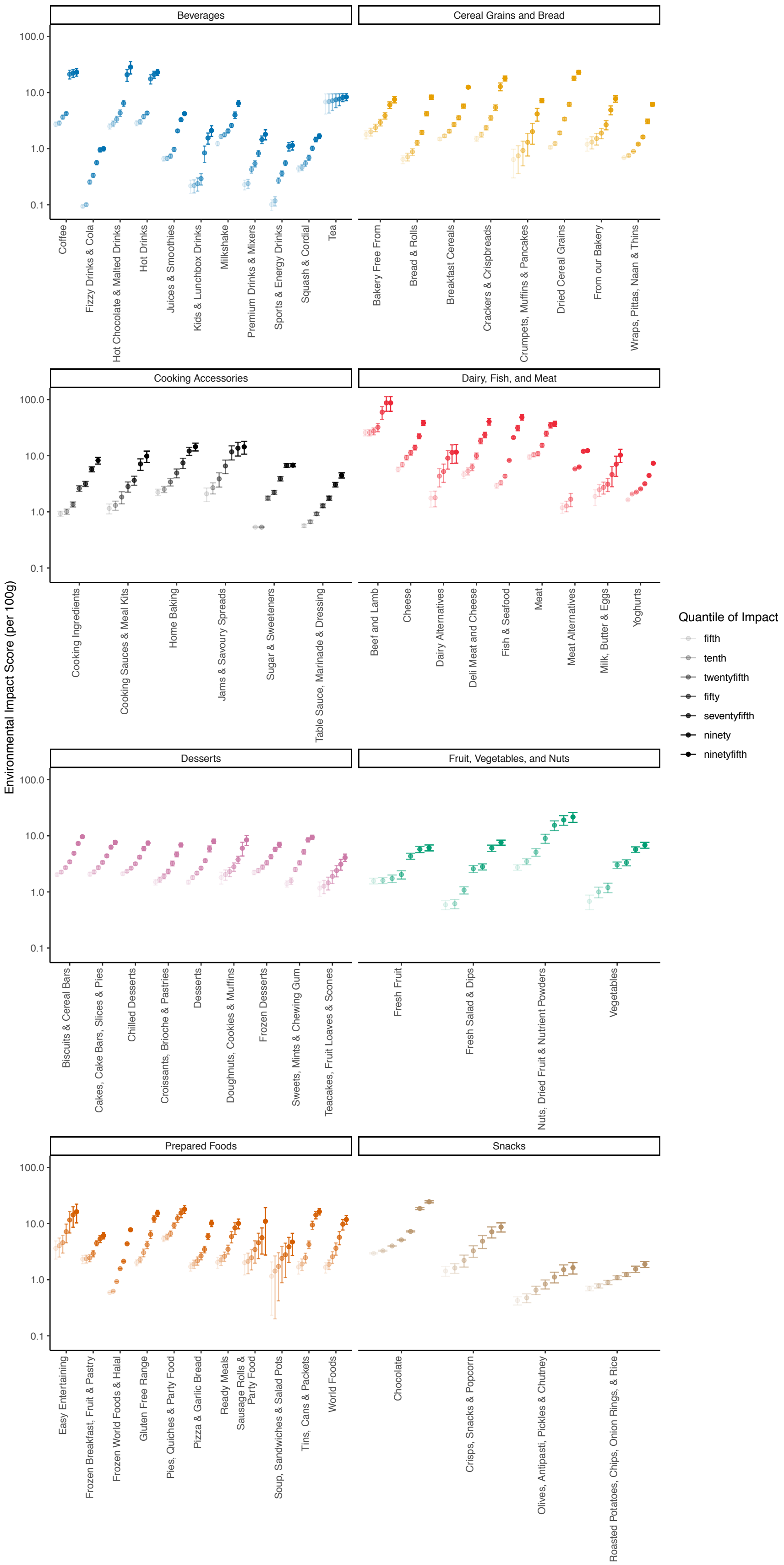

Beverages
Cereal Grains and Bread
Cooking Accessories
Dairy, Fish, and Meat
Desserts
Fruit, Vegetables, and Nuts
Prepared Foods
Snacks
Environmental Impact Score (per 100g)
100.0
10.0
1.0
0.1
Coffee
Fizzy Drinks & Cola
Hot Chocolate & Malted Drinks
Hot Drinks
Juices & Smoothies
Kids & Lunchbox Drinks
Milkshake
Premium Drinks & Mixers
Sports & Energy Drinks
Squash & Cordial
Tea
Bakery Free From
Bread & Rolls
Breakfast Cereals
Crackers & Crispbreads
Crumpets, Muffins & Pancakes
Dried Cereal Grains
From our Bakery
Wraps, Pittas, Naan & Thins
Cooking Ingredients
Cooking Sauces & Meal Kits
Home Baking
Jams & Savoury Spreads
Sugar & Sweeteners
Table Sauce, Marinade & Dressing
Beef and Lamb
Cheese
Dairy Alternatives
Deli Meat and Cheese
Fish & Seafood
Meat
Meat Alternatives
Milk, Butter & Eggs
Yoghurts
Biscuits & Cereal Bars
Cakes, Cake Bars, Slices & Pies
Chilled Desserts
Croissants, Brioche & Pastries
Desserts
Doughnuts, Cookies & Muffins
Frozen Desserts
Sweets, Mints & Chewing Gum
Teacakes, Fruit Loaves & Scones
Fresh Fruit
Fresh Salad & Dips
Nuts, Dried Fruit & Nutrient Powders
Vegetables
Easy Entertaining
Frozen Breakfast, Fruit & Pastry
Frozen World Foods & Halal
Gluten Free Range
Pies, Quiches & Party Food
Pizza & Garlic Bread
Ready Meals
Sausage Rolls & Party Food
Soup, Sandwiches & Salad Pots
Tins, Cans & Packets
World Foods
Chocolate
Crisps, Snacks & Popcorn
Olives, Antipasti, Pickles & Chutney
Roasted Potatoes, Chips, Onion Rings, & Rice
Quantile of Impact
fifth
tenth
twentyfifth
fifty
seventyfifth
ninety
ninetyfifth

Figure S13. Sensitivity of the environmental impacts of Tesco products to uncertainty in ingredient sourcing. Points show mean and error bars show 95% confidence intervals around the mean. Shading of points and bars indicates the quantile of the impact, where the quantile is derived from the Monte Carlo analysis. As such, the e.g. fifth quantile corresponds with the 5th % of 1,000 estimated impacts for the product as calculated during the Monte Carlo analysis.

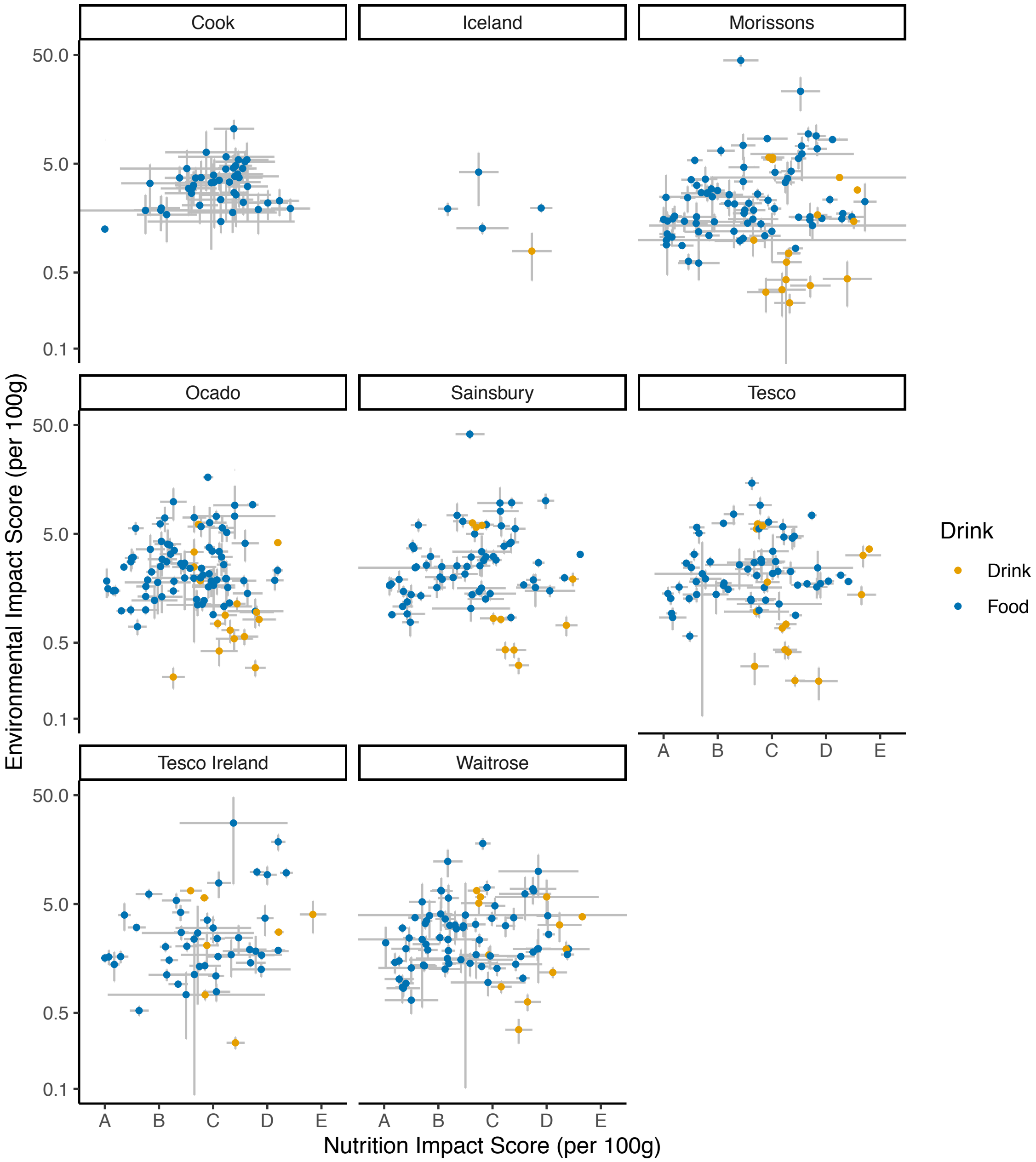

Fig. S14. Environmental and nutrition impact score for each Aisle across the eight retailers in the analysis. Each point indicates the average environmental and nutrition impact score for all products in an Aisle while error bars indicate the 95% confidence interval in the environmental and nutrition impacts of products in that Aisle. Points are colored by whether the Aisle contains food products (blue) or drink products (red). We did not include seasonal foods (e.g. Halloween confectionaries) to avoid skewing results from products that are not consistently available for purchase, and did not include alcoholic beverages because NutriScore does not score these.

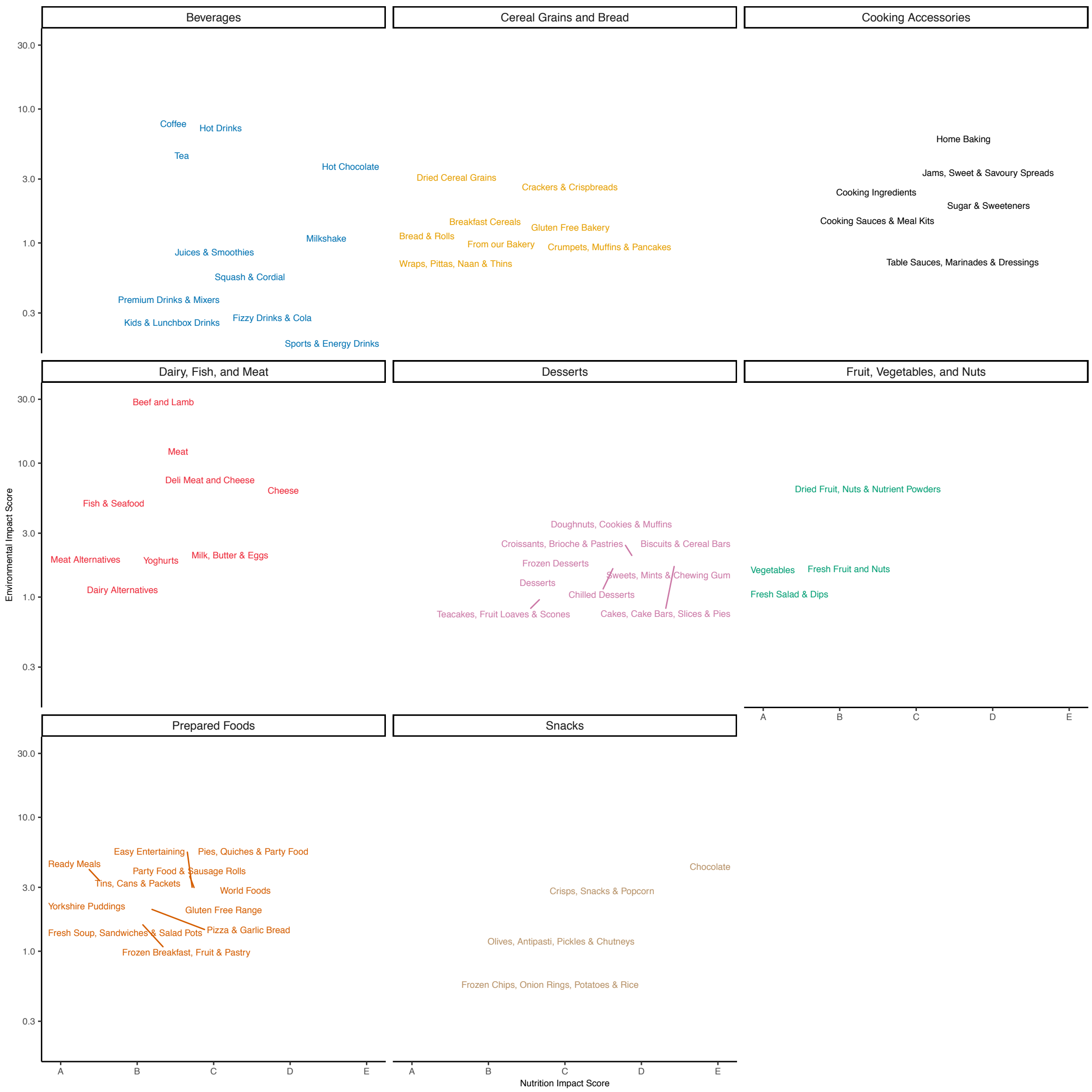

Fig. S15. Environmental and nutrition impact score of Tesco retail 'Aisles'. Aisles are separated into Food Types, with one panel for each Food Type. When plotting, Aisles containing similar products were condensed for visibility and clarity (see Supplemental Methods). For instance, the Aisles "Fresh Vegetables" and "Frozen Vegetables" were condensed into "Vegetables". Labels were jittered to avoid overlap.

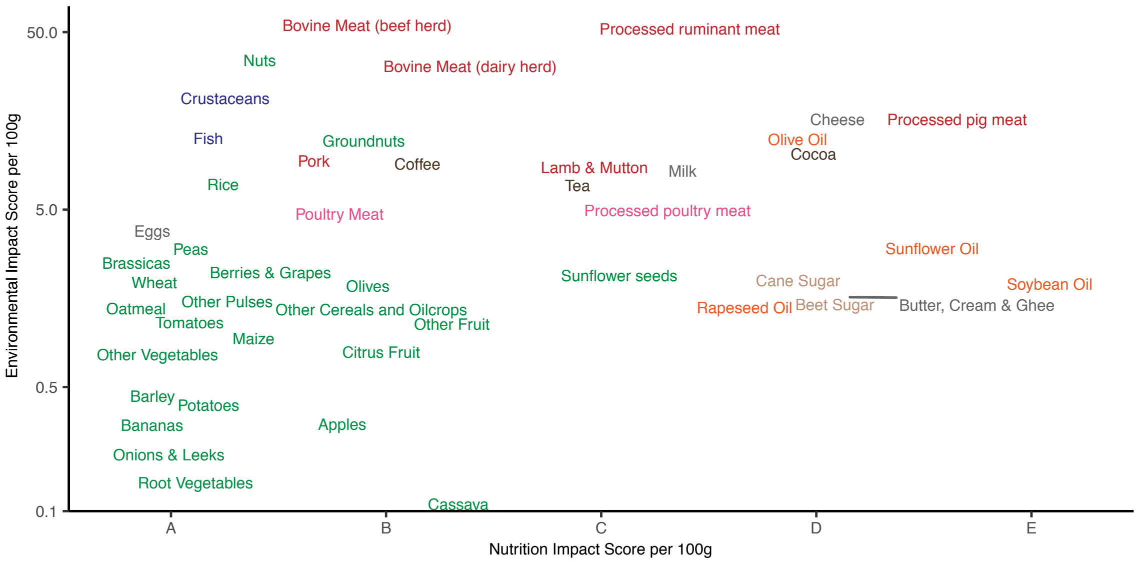

Fig. S16. Environmental and nutrition impact of single-ingredient foods. Single-ingredient foods were those where >99% of the product was composed of one ingredient. Points and labels are jittered to avoid overlap, and are colored by food type where: green = plant-based foods; light brown = sugar; dark brown = chocolate, coffee, and tea; orange = oils; grey = dairy and eggs; pink = poultry; blue = seafood; and red = red meat. Comparison is limited to the primary environmental database food categories. Environmental impact information for the environmental database food categories is available in **Figure S17**.

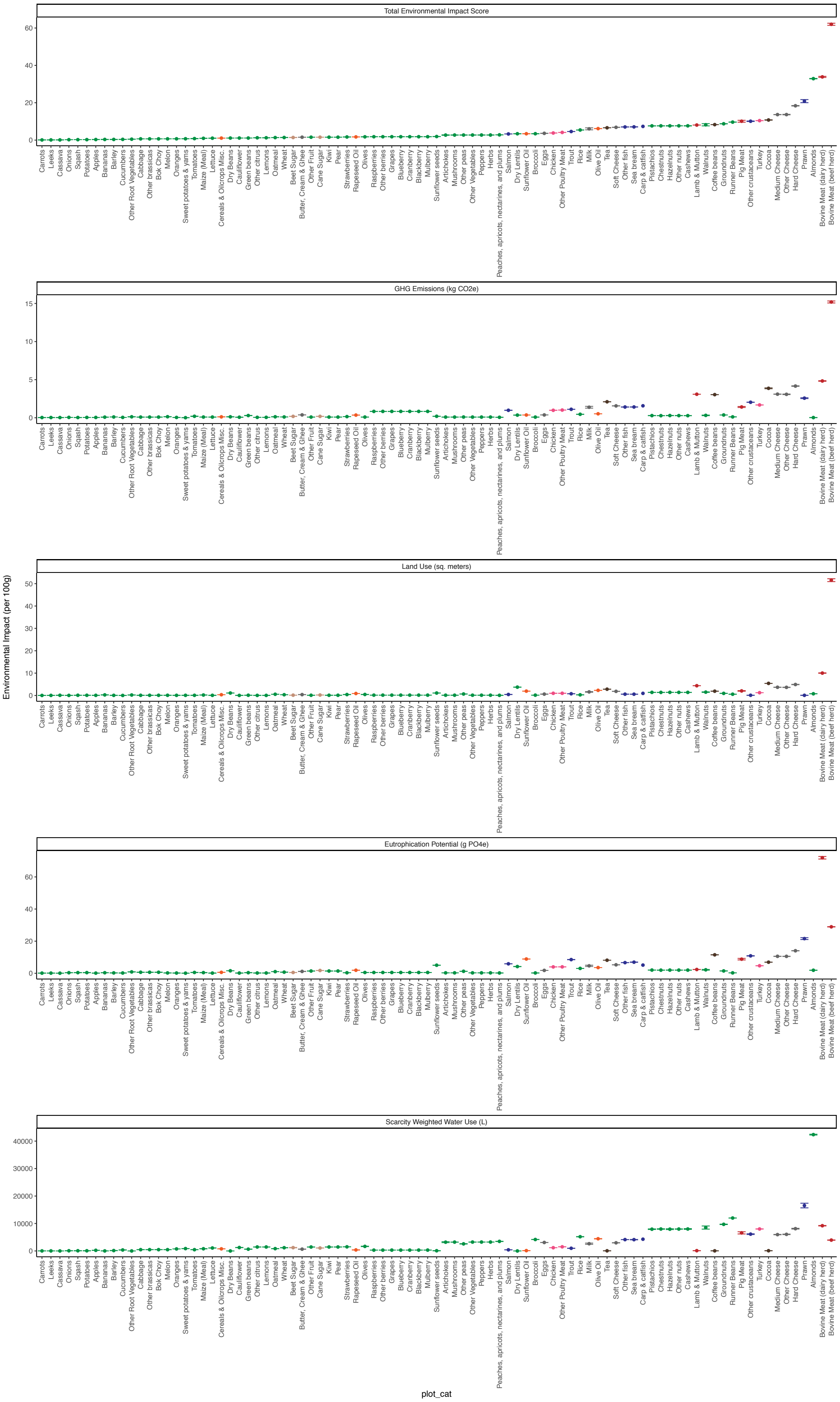

Total Environmental Impact Score
GHG Emissions (kg CO2e)
Land Use (sq. meters)
Eutrophication Potential (g PO4e)
Scarcity Weighted Water Use (L)
Environmental Impact (per 100g)
plot_cat
Carrots
Leeks
Cassava
Onions
Squash
Potatoes
Apples
Bananas
Barley
Cucumbers
Other Root Vegetables
Cabbage
Other brassicas
Bok Choy
Melon
Oranges
Sweet potatoes & yams
Tomatoes
Maize (Meal)
Lettuce
Cereals & Oilcrops Misc.
Dry Beans
Cauliflower
Green beans
Other citrus
Lemons
Oatmeal
Wheat
Beet Sugar
Butter, Cream & Ghee
Other Fruit
Cane Sugar
Kiwi
Pear
Strawberries
Rapeseed Oil
Olives
Raspberries
Other berries
Grapes
Blueberry
Cranberry
Blackberry
Mulberry
Sunflower seeds
Artichokes
Mushrooms
Other peas
Other Vegetables
Peppers
Herbs
Peaches, apricots, nectarines, and plums
Salmon
Dry Lentils
Sunflower Oil
Broccoli
Eggs
Chicken
Other Poultry Meat
Trout
Rice
Milk
Olive Oil
Tea
Soft Cheese
Other fish
Sea bream
Carp & catfish
Pistachios
Chestnuts
Hazelnuts
Other nuts
Cashews
Lamb & Mutton
Walnuts
Coffee beans
Groundnuts
Runner Beans
Pig Meat
Other crustaceans
Turkey
Cocoa
Medium Cheese
Other Cheese
Hard Cheese
Prawn
Almonds
Bovine Meat (dairy herd)
Bovine Meat (beef herd)

Figure S17. Environmental impacts of the environmental database food categories used in this analysis. Categories are sorted from lowest to highest overall environmental impact score, with panels showing the impact across the different environmental indicators used in the analysis. Points are colored by food type where: green = plant-based foods; light brown = sugar; dark brown = chocolate, coffee, and tea; orange = oils; grey = dairy and eggs; pink = poultry; blue = seafood; and red = red meat. Note that the plot shows all of the food categories used to classify ingredients. In some cases, multiple food categories shown in the plot have identical environmental impact estimates (as is the case with e.g. pistachios, cashews, and several other types of tree nuts). This is because there were fewer than 5 environmental data observations for these commodities in the environmental databases used in this analysis.

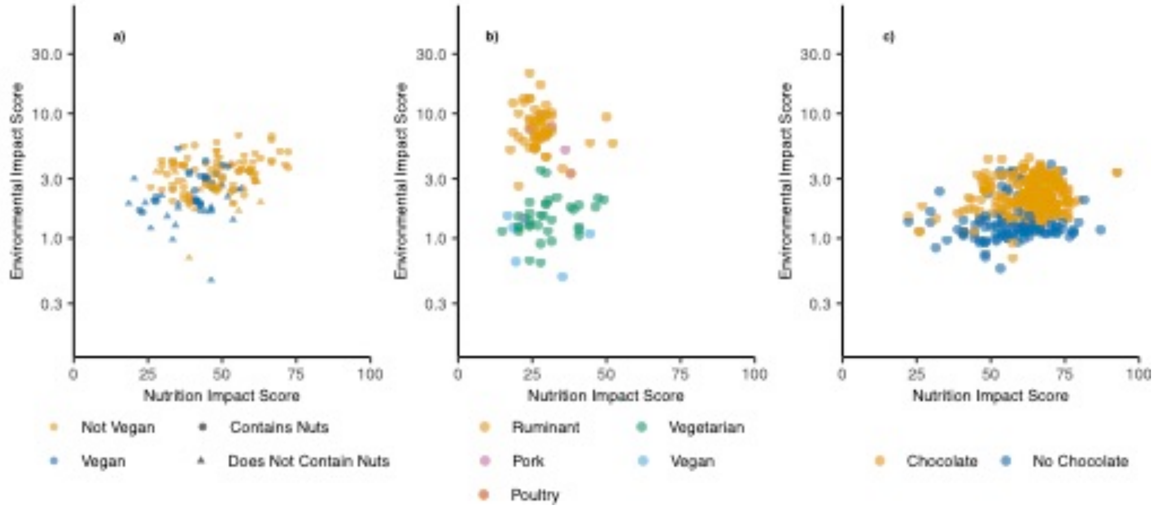

Figure S18. Variation in environmental impact and nutrition impact scores per 100g of (a) pestos, (b) lasagna, and (c) cookies. Each point indicates a single food product. Points are colored to indicate different food types and are partially transparent. Products were identified based on the retail Aisle and Shelf they were categorized in and their product name. Data includes 161 pesto sauces, 413 cookies, and 107 lasagne.

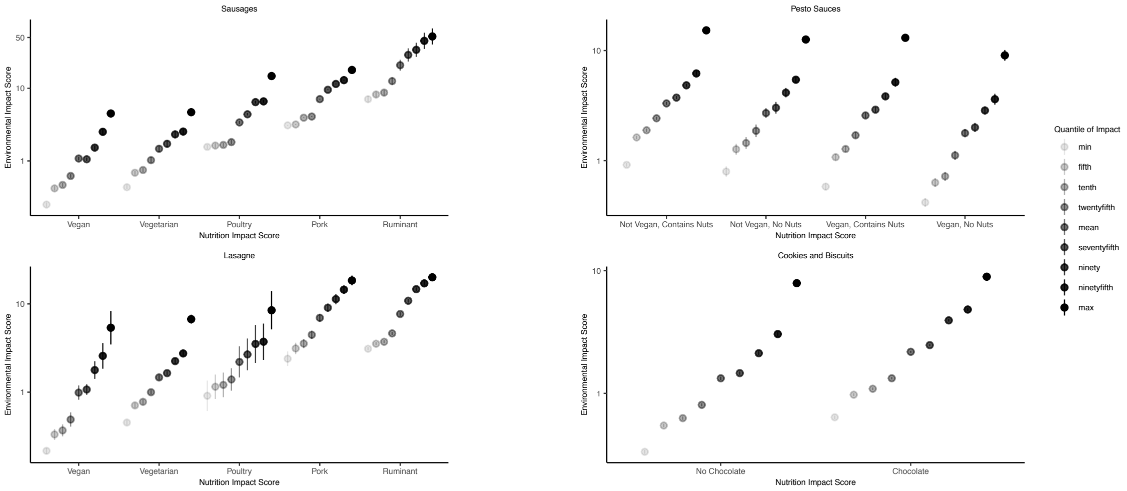

Fig. S19. The effect of ingredient sourcing on the environmental impacts of sausages, pesto sauces, lasagna, and cookies. Points show mean and error bars show mean +- 1.s.e.m. Shading of points and bars indicates the quantile of the impact, where the quantile is derived from the Monte Carlo analysis. As such, the e.g. fifth quantile corresponds with the 5th % of 1,000 estimated impacts for the product as calculated during the Monte Carlo analysis. Data includes 503 sausages, 161 pesto sauces, 413 cookies, and 107 lasagne.

Supplementary Tables

Table S1. Number of ingredients that had a listed percent composition in the back-of-package ingredient lists. Separated by Retailer, and by primary ingredients (e.g. a tomato sauce in a lasagna) and secondary (e.g. the tomatoes in a tomato sauce in a lasagna).

Retailer	Is it a primary ingredient?	Number of ingredients with a listed percent composition	Total number of ingredients	Percent of ingredients with a listed percent composition
Cook	Yes	6440	25923	24.84
Cook	No	319	29030	1.10
Iceland	Yes	784	8803	8.91
Iceland	No	112	5798	1.93
Morissons	Yes	19585	142996	13.70
Morissons	No	1785	86496	2.06
Ocado	Yes	55128	312551	17.64
Ocado	No	5275	139729	3.78
Sainsbury	Yes	45379	294321	15.42
Sainsbury	No	6836	179716	3.80
Tesco	Yes	45748	404365	11.31
Tesco	No	3683	205155	1.80
Tesco Ireland	Yes	8229	63913	12.88
Tesco Ireland	No	702	30322	2.32
Waitrose	Yes	24826	184254	13.47
Waitrose	No	2029	87823	2.31
All Retailers	Yes	206119	1437126	14.34
All Retailers	No	20741	764069	2.71
All Retailers	All Ingredients	226860	2201195	10.31

Table S2. Number of ingredients sorted into each of the 110 environmental database food categories used in the analysis. NA values in the secondary and tertiary environmental database food categories indicate that there are not adequate observations in the environmental databases to further divide the primary category into more specific secondary and tertiary categories.

Primary Environmental Database Food Category	Secondary Environmental Database Food Category	Tertiary Environmental Database Food Category	Number of ingredients identified	Percent of total ingredients
Animal Fats	NA	NA	341	0.01%
Apples	NA	NA	15737	0.65%
Bananas	NA	NA	2445	0.10%
Barley (Beer)	Barley	NA	32632	1.35%
Barley (Beer)	Beer	NA	116	0.00%
Beet Sugar	NA	NA	1904	0.08%
Berries & Grapes	Grapes	NA	9472	0.39%
Berries & Grapes	Other berries	Blackberry	1284	0.05%
Berries & Grapes	Other berries	Blueberry	1921	0.08%
Berries & Grapes	Other berries	Cranberry	2470	0.10%
Berries & Grapes	Other berries	Currants	4195	0.17%
Berries & Grapes	Other berries	Mulberry	42	0.00%
Berries & Grapes	Other berries	Other berries	5245	0.22%
Berries & Grapes	Raspberries	NA	5965	0.25%
Berries & Grapes	Strawberries	NA	5931	0.25%
Bovine Meat (beef herd)	NA	NA	1188	0.05%
Bovine Meat (dairy herd)	NA	NA	7527	0.31%
Brassicas	Broccoli and cauliflower	Broccoli	1167	0.05%
Brassicas	Broccoli and cauliflower	Cauliflower	955	0.04%
Brassicas	Cabbage	NA	1581	0.07%
Brassicas	Other brassicas	Bok Choy	100	0.00%
Brassicas	Other brassicas	Other brassicas	816	0.03%
Butter, Cream & Ghee	NA	NA	24971	1.03%
Cane Sugar	NA	NA	176746	7.32%
Cassava	NA	NA	8279	0.34%
Cereals & Oilcrops Misc.	NA	NA	74099	3.07%
Cheese	Hard Cheese	NA	1254	0.05%
Cheese	Medium Cheese	NA	2394	0.10%
Cheese	Other Cheese	NA	2152	0.09%
Cheese	Soft Cheese	NA	2068	0.09%
Citrus Fruit	Oranges	NA	12162	0.50%
Citrus Fruit	Other citrus	Lemons	26827	1.11%
Citrus Fruit	Other citrus	Other citrus	5603	0.23%
Coffee	Brewed coffee	NA	41	0.00%
Coffee	Coffee beans	NA	4750	0.20%
Crustaceans (farmed)	Other Crustaceans (farmed)	NA	1424	0.06%
Crustaceans (farmed)	Prawn	NA	781	0.03%
Crustaceans (farmed)	Shrimp	NA	191	0.01%
Dark Chocolate	Chocolate	NA	2334	0.10%
Dark Chocolate	Cocoa	NA	50447	2.09%
Eggs	NA	NA	18149	0.75%
Fish (farmed)	Carp & catfish	NA	18	0.00%
Fish (farmed)	Other farmed fish	NA	5950	0.25%
Fish (farmed)	Sea bream	NA	61	0.00%
Fish (farmed)	Trout & salmon	Salmon	1051	0.04%
Fish (farmed)	Trout & salmon	Trout	42	0.00%
Groundnuts	NA	NA	2315	0.10%
Lamb & Mutton	NA	NA	1130	0.05%
Maize (Meal)	NA	NA	55417	2.29%
Milk	NA	NA	102472	4.24%
Milk Chocolate	NA	NA	6575	0.27%
Nuts	Almonds	NA	4306	0.18%
Nuts	Other nuts	Cashews	1544	0.06%
Nuts	Other nuts	Chestnuts	268	0.01%
Nuts	Other nuts	Hazelnuts	2521	0.10%
Nuts	Other nuts	Other nuts	1926	0.08%
Nuts	Other nuts	Pistachios	413	0.02%
Nuts	Other nuts	Walnuts	527	0.02%
Oatmeal	Oatmeal	NA	7302	0.30%
Oatmeal	Oatmilk	NA	1	0.00%
Olive Oil	Olive Oil	NA	6741	0.28%
Olives	NA	NA	2205	0.09%
Onions & Leeks	Leeks	NA	2816	0.12%
Onions & Leeks	Onions	NA	47274	1.96%

Table S2, continued

Primary Environmental Database Food Category	Secondary Environmental Database Food Category	Tertiary Environmental Database Food Category	Number of ingredients identified	Percent of total ingredients
Other Fruit	Kiwi	NA	567	0.02%
Other Fruit	Melon	NA	704	0.03%
Other Fruit	Other Fruit	NA	37608	1.56%
Other Fruit	Peaches, apricots, nectarines, and	NA	8436	0.35%
Other Fruit	Pear	NA	1889	0.08%
Other Pulses	Other Dry Pulses	Dry Beans	26818	1.11%
Other Pulses	Other Dry Pulses	Dry Lentils	2033	0.08%
Other Pulses	Runner Beans	NA	40	0.00%
Other Vegetables	Cucumbers and squash	Cucumbers	1265	0.05%
Other Vegetables	Cucumbers and squash	Sqash	3177	0.13%
Other Vegetables	Green beans	NA	76	0.00%
Other Vegetables	Herbs	NA	62051	2.57%
Other Vegetables	Lettuce	NA	10957	0.45%
Other Vegetables	Mushrooms	NA	6024	0.25%
Other Vegetables	Other Vegetables	Artichokes	165	0.01%
Other Vegetables	Other Vegetables	Other Vegetables	14635	0.61%
Other Vegetables	Peppers	NA	71491	2.96%
Palm Oil	NA	NA	26466	1.10%
Peas	Other peas	NA	7643	0.32%
Pig Meat	NA	NA	13829	0.57%
Potatoes	Potatoes	NA	25701	1.06%
Potatoes	Sweet potatoes & yams	NA	1669	0.07%
Poultry Meat	Chicken	NA	10722	0.44%
Poultry Meat	Other Poultry Meat	NA	517	0.02%
Poultry Meat	Turkey	NA	772	0.03%
Rapeseed Oil	NA	NA	11559	0.48%
Rice	NA	NA	33307	1.38%
Root Vegetables	Carrots	NA	15320	0.63%
Root Vegetables	Other Root Vegetables	NA	67354	2.79%
Salt	NA	NA	133811	5.54%
Soybean Oil	NA	NA	634	0.03%
Soymilk	NA	NA	14	0.00%
Sunflower Oil	NA	NA	27910	1.16%
Sunflower seeds	NA	NA	3210	0.13%
Tea	NA	NA	3973	0.16%
Tofu	NA	NA	144	0.01%
Tomatoes	NA	NA	33344	1.38%
Water	NA	NA	101540	4.20%
Wheat & Rye (Bread)	Bread	NA	705	0.03%
Wheat & Rye (Bread)	Rye	NA	431	0.02%
Wheat & Rye (Bread)	Wheat	NA	92158	3.82%
Wine	Grapes	NA	49	0.00%
Wine	Other Wine	NA	1566	0.06%
Wine	Red wine	NA	3043	0.13%
Wine	White wine	NA	5119	0.21%
Unsorted ingredients	Unsorted ingredients	Unsorted ingredients	774236	32.06%
Grand Total	Grand Total	Grand Total	2415263	100.00%

Table S3. Results from pairwise Spearman's correlations between the estimated impacts of each product across environmental indicators.

Indicator 1	Indicator 2	Spearman's Rho	P-value
Eutrophication Potential	Eutrophication Potential	1.000	<0.0001
Eutrophication Potential	Greenhouse Gas Emissions	0.858	<0.0001
Eutrophication Potential	Land Use	0.825	<0.0001
Eutrophication Potential	Water Scarcity	0.430	<0.0001
Greenhouse Gas Emissions	Eutrophication Potential	0.858	<0.0001
Greenhouse Gas Emissions	Greenhouse Gas Emissions	1.000	<0.0001
Greenhouse Gas Emissions	Land Use	0.770	<0.0001
Greenhouse Gas Emissions	Water Scarcity	0.336	<0.0001
Land Use	Eutrophication Potential	0.825	<0.0001
Land Use	Greenhouse Gas Emissions	0.770	<0.0001
Land Use	Land Use	1.000	<0.0001
Land Use	Water Scarcity	0.301	<0.0001
Water Scarcity	Eutrophication Potential	0.430	<0.0001
Water Scarcity	Greenhouse Gas Emissions	0.336	<0.0001
Water Scarcity	Land Use	0.301	<0.0001
Water Scarcity	Water Scarcity	1.000	<0.0001

Table S4. Difference in the estimated and known percent composition of validated products by order of ingredient in the food product. P-values in the table are from paired t-tests. Top half of the table is across all iterations of the validation approach, whereas bottom half is across only the iterations where no information about the percent composition of ingredients in the product was known.

	Order of Ingredient	P-Value	Estimated Composition (from algorithm; % of total product)	Known Composition (from packaging information; % of total product)	Difference in Composition (estimated - known; % of total product)	Lower CI of difference in composition (% of product)	Upper CI of difference in composition (% of product)	Degrees of Freedom
Tests Across All Validated Products, Independent of the Product's Known Composition	1	< 0.001	48.11	46.82	1.29	1.16	1.43	242576.87
	2	< 0.001	20.23	19.78	0.45	0.39	0.52	242305.02
	3	< 0.001	12.75	13.32	-0.57	-0.62	-0.53	232127.66
	4	< 0.001	7.77	8.25	-0.48	-0.51	-0.45	222036.64
	5	< 0.001	5.58	6.01	-0.43	-0.46	-0.41	206471.41
	6	< 0.001	4.04	4.19	-0.15	-0.17	-0.13	188387.78
	7	< 0.001	3.06	3.11	-0.05	-0.07	-0.03	166911.18
	8	< 0.001	2.39	2.62	-0.24	-0.26	-0.22	139891.50
	9	< 0.001	1.87	1.89	-0.01	-0.03	0.01	84002.63
	10	< 0.001	1.25	1.43	-0.18	-0.20	-0.16	62212.29
	11	< 0.001	0.73	0.99	-0.26	-0.28	-0.23	30153.11
	12	< 0.001	0.08	0.10	-0.02	-0.02	-0.02	844.00
Tests When Known Composition of the Product Was 0%	1	0.07285627	72.16	72.84	-0.68	-1.41	0.06	12670.65
	2	< 0.001	19.47	17.75	1.72	1.27	2.17	12292.96
	3	< 0.001	9.91	10.63	-0.73	-1.09	-0.36	6805.41
	4	< 0.001	5.95	6.93	-0.98	-1.26	-0.69	3820.25
	5	< 0.001	3.47	4.13	-0.66	-0.90	-0.43	2436.95
	6	0.326	2.31	2.40	-0.09	-0.27	0.09	1819.83
	7	0.055	2.29	2.11	0.18	0.00	0.37	838.53
	8	0.001	1.82	2.17	-0.35	-0.55	-0.14	542.19
	9	0.098	1.59	1.45	0.14	-0.03	0.31	287.99
	10	0.61	0.93	0.88	0.05	-0.13	0.22	242.92
	11	0.381	0.30	0.24	0.06	-0.08	0.20	253.51
	12				Not enough information			

Table S5. Difference in the estimated and known percent composition of validated products across environmental database food categories. P-values in the table are from paired t-tests. Left half of the table is across all iterations of the validation approach, whereas right half is across only the iterations where no information about the percent composition of ingredients in the product was known.

	Analyses across all validated products					Analyses when assuming the composition of no ingredient is known				
Environmental Database Food Category	Percent estimated (from algorithm; % of total product)	Percent known (from packaging; % of total product)	Difference between percent estimated and known (% of total product)	Standard error of the difference between estimated and known composition (% of total product)	Number of obesrvations	Percent estimated (from algorithm; % of total product)	Percent known (from packaging; % of total product)	Difference between percent estimated and known (% of total product)	Standard error of the difference between estimated and known composition (% of total product)	Number of obesrvations
Apples	42.18	41.84	0.34	0.03	40688	49.15	51.83	-2.68	0.41	995
Bananas	8.22	8.52	-0.30	0.01	16190	21.11	22.24	-1.13	0.51	171
Barley	16.20	16.48	-0.29	0.07	3907	41.96	36.34	5.61	2.91	21
Beet Sugar	95.32	95.10	0.22	0.52	10	95.54	95.10	0.44	1.09	5
Berries & Grapes	11.06	11.15	-0.09	0.01	120536	19.33	18.60	0.73	0.22	2239
Bovine Meat (beef herd)	44.87	38.42	6.45	1.06	66	87.44	85.60	1.84	6.11	5
Bovine Meat (dairy herd)	9.40	9.88	-0.48	0.03	1026	42.23	41.43	0.80	1.74	7
Brassicas	4.81	4.85	-0.04	0.01	38581	17.30	18.31	-1.02	0.51	388
Butter, Cream & Ghee	13.80	13.80	0.00	0.04	2110	28.69	30.46	-1.77	0.64	61
Cane Sugar	24.04	23.23	0.81	0.40	840	32.13	30.36	1.77	0.99	230
Cassava	2.65	6.14	-3.49	0.28	158	0.10	8.10	-8.01	0.84	29
Citrus Fruit	8.60	8.29	0.31	0.01	54154	19.41	17.56	1.85	0.30	1256
Cocoa	4.86	5.28	-0.41	0.01	30751	13.94	15.35	-1.41	0.57	287
Coffee	51.00	50.96	0.04	0.13	2030	57.31	57.26	0.04	0.30	888
Crustaceans	40.74	41.66	-0.92	1.39	58	46.08	46.90	-0.82	2.86	20
Eggs	23.76	26.48	-2.72	0.78	125	20.92	26.54	-5.62	1.49	62
Fish	83.32	83.53	-0.21	0.25	81	90.27	90.29	-0.02	0.46	28
Groundnuts	44.99	44.77	0.21	0.16	855	63.65	64.25	-0.60	0.96	85
Lamb & Mutton	95.46	99.00	-3.54	3.84	4	91.92	99.00	-7.08	7.98	2
Maize	22.05	22.88	-0.83	0.07	5361	39.67	42.08	-2.41	0.46	422
Milk	32.98	35.01	-2.03	0.24	1339	36.86	45.32	-8.46	1.18	174
Milk Chocolate	58.65	59.00	-0.35	0.58	12	60.92	59.00	1.92	0.00	2
Nuts	13.27	13.55	-0.28	0.02	33638	29.35	29.39	-0.05	0.51	641
Oatmeal	25.49	25.80	-0.31	0.04	11512	59.09	58.39	0.70	0.60	365
Olive Oil	4.98	5.15	-0.17	0.03	3431	11.84	12.42	-0.59	0.43	175
Olives	55.74	48.83	6.91	1.28	75	63.39	46.33	17.06	3.16	21
Onions & Leeks	17.76	17.15	0.61	0.04	7505	31.19	23.46	7.73	1.28	74
Other Cereals and Oilcrops	12.90	13.22	-0.32	0.01	70286	16.22	16.55	-0.33	0.24	1741
Other Fruit	17.21	17.00	0.21	0.01	136769	34.27	34.90	-0.63	0.28	2482
Other Pulses	11.83	11.69	0.13	0.02	26987	28.50	27.87	0.63	0.80	324
Other Vegetables	9.71	9.81	-0.10	0.01	77407	16.42	15.76	0.66	0.34	976
Palm Oil	14.93	7.69	7.24	1.79	36	18.98	6.81	12.16	3.05	17
Peas	21.43	22.02	-0.59	0.09	3444	34.75	36.48	-1.73	0.65	213
Pork	77.00	85.00	-8.00	3.12	8	69.00	85.00	-16.00	1.66	4
Potatoes	27.59	28.01	-0.42	0.10	2396	81.67	86.95	-5.28	0.81	232
Poultry Meat	40.34	43.39	-3.05	0.59	72	83.89	90.25	-6.36	2.65	8
Rapeseed Oil	6.33	2.03	4.30	0.08	2011	14.97	4.94	10.03	1.65	66
Rice	24.96	25.37	-0.41	0.05	11522	44.81	46.49	-1.68	0.37	1004
Root Vegetables	10.21	9.89	0.32	0.02	37281	29.33	28.23	1.10	0.45	889
Salt	1.92	1.64	0.28	0.02	12924	4.08	3.26	0.82	0.32	558
Sunflower Oil	7.11	6.88	0.24	0.06	4599	14.01	9.26	4.75	0.42	575
Sunflower seeds	11.61	12.36	-0.75	0.01	32373	19.22	17.78	1.44	0.73	130
Tea	68.69	73.12	-4.43	0.20	4626	69.67	82.22	-12.55	0.75	701
Tomatoes	26.84	27.51	-0.67	0.05	6416	56.11	55.70	0.40	0.78	273
Water	16.80	17.90	-1.09	0.10	9027	27.53	36.56	-9.03	1.45	231
Wheat	16.43	16.55	-0.11	0.06	8533	55.93	56.85	-0.92	1.12	336
Wine	3.22	3.07	0.16	0.03	1654	12.62	12.52	0.10	0.35	123
Uncategorised Ingredients	5.51	4.93	0.59	0.01	97840	10.26	5.64	4.62	0.20	2661

Table S6. Spearman's Rho regression between the environmental impact score and nutrition impact score across Aisles in each Retailer (as shown in **Figure S4**), further separated by correlations on only food products (top), only drinks (middle), or food and drinks (bottom). We did not include seasonal foods (e.g. Halloween confectionaries) to avoid skewing results from products that are not consistently available for purchase, and did not include alcoholic beverages because NutriScore does not score these. Correlations left of the vertical line were conducted on the estimated mean environmental and nutrition impact for each Aisle. Correlations right of the vertical line were conducted by randomly sampling environmental and nutrition impact data from within the observed impacts of each Aisle, and repeating this 1,000 times.

		Correlations on mean impacts		Corrleations on randomly sampled impacts for each Aisle								
	Retailer	Spearman's Rho	P-Value	Proportion of correlations that were significant	Mean Spearman's Rho	se Spearman's Rho	Minimum Observed Spearman's Rho	Maximum Observed Spearman's rho	Mean P-value	se P-Value	Minimum Observed P-value	Maximum Observed P-value
	Across All Retailers	0.258	<0.001	1.000	0.251	0.000	0.215	0.284	<0.001	<0.001	<0.001	<0.001
	Cook	0.230	0.128	0.036	0.185	0.002	0.004	0.349	0.257	0.005	0.019	0.978
	Iceland	0.000	1.000	0.000	-0.231	0.009	-0.800	0.400	0.772	0.009	0.333	1.000
	Morissons	0.319	0.005	1.000	0.314	0.001	0.234	0.400	0.007	<0.001	<0.001	0.042
Foods	Ocado	0.140	0.217	0.000	0.122	0.001	0.031	0.198	0.298	0.004	0.080	0.789
	Sainsbury	0.364	0.004	1.000	0.360	0.001	0.293	0.438	0.005	<0.001	<0.001	0.021
	Tesco	0.205	0.122	0.009	0.205	0.001	0.124	0.287	0.129	0.002	0.029	0.353
	Tesco Ireland	0.297	0.054	0.207	0.281	0.001	0.196	0.356	0.072	0.001	0.020	0.206
	Waitrose	0.241	0.046	0.548	0.239	0.001	0.161	0.314	0.052	0.001	0.009	0.186
	Single Ingredient Foods	0.382	<0.001	1.000	0.383	0.000	0.353	0.397	<0.001	<0.001	<0.001	<0.001
	Across All Retailers	-0.229	0.043	0.533	-0.221	0.001	-0.289	-0.138	0.056	0.001	0.010	0.226
	Cook					Not Enough Data Points						
	Iceland					Not Enough Data Points						
	Morissons	-0.007	0.981	0.000	0.007	0.003	-0.304	0.257	0.762	0.005	0.235	1.000
Drinks	Ocado	-0.176	0.497	0.000	-0.171	0.002	-0.252	0.091	0.507	0.003	0.327	0.996
	Sainsbury	-0.661	0.044	0.637	-0.636	0.001	-0.673	-0.515	0.056	0.001	0.039	0.133
	Tesco	-0.150	0.593	0.000	-0.142	0.001	-0.214	-0.061	0.614	0.002	0.442	0.832
	Tesco Ireland	-0.393	0.396	0.000	-0.396	0.001	-0.571	-0.214	0.392	0.001	0.200	0.662
	Waitrose	-0.252	0.430	0.000	-0.279	0.002	-0.392	-0.091	0.385	0.003	0.210	0.783
	Single Ingredient Foods	-1.000	0.333	0.000	-1.000	0.001	-1.000	-0.500	0.334	0.001	0.333	1.000
	Across All Retailers	0.103	0.019	0.913	0.098	0.000	0.072	0.125	0.029	<0.001	0.004	0.101
	Cook	0.230	0.128	0.038	0.187	0.002	-0.011	0.373	0.247	0.005	0.012	0.943
	Iceland	-0.200	0.783	0.000	-0.378	0.006	-0.700	0.100	0.590	0.007	0.233	1.000
	Morissons	0.136	0.195	0.000	0.127	0.001	0.052	0.195	0.236	0.003	0.061	0.618
Foods and Drinks	Ocado	-0.077	0.456	0.000	-0.087	0.001	-0.156	-0.028	0.406	0.003	0.128	0.784
	Sainsbury	0.122	0.308	0.000	0.121	0.001	0.058	0.175	0.315	0.002	0.141	0.628
	Tesco	0.025	0.830	0.000	0.027	0.001	-0.031	0.083	0.814	0.003	0.486	1.000
	Tesco Ireland	0.265	0.064	0.055	0.248	0.001	0.159	0.308	0.085	0.001	0.030	0.269
	Waitrose	0.154	0.170	0.000	0.152	0.001	0.086	0.209	0.183	0.002	0.061	0.444
	Single Ingredient Foods	0.397	<0.001	1.000	0.398	0.000	0.376	0.412	<0.001	<0.001	<0.001	<0.001

Table S7. Spearman’s Rho regression results across Aisles in each Retailer, using only products where full ingredient composition information was provided, and further separated by correlations on only food products (top), only drinks (middle), or food and drinks (bottom). We did not include seasonal foods (e.g. Halloween confectionaries) to avoid skewing results from products that are not consistently available for purchase, and did not include alcoholic beverages because NutriScore does not score these. Correlations left of the vertical line were conducted on the estimated mean environmental and nutrition impact for each Aisle. Correlations right of the vertical line were conducted by randomly sampling environmental and nutrition impact data from within the observed impacts of each Aisle, and repeating this 1,000 times.

		Correlations on mean impacts for each Aisle		Corrleations on randomly sampled impacts for each Aisle								
	Retailer	Spearman's Rho	P-Value	Proportion of correlations that were significant	Mean Spearman's Rho	se Spearman's Rho	Minimum Observed Spearman's Rho	Maximum Observed Spearman's rho	Mean P-value	se P-Value	Minimum Observed P-value	Maximum Observed P-value
Foods	All Retailers	0.324	<0.001	1.000	0.294	0.001	0.222	0.350	<0.001	<0.001	<0.001	0.002
	Morissons	0.262	0.170	0.012	0.247	0.002	0.011	0.396	0.220	0.004	0.033	0.955
	Ocado	0.230	0.115	0.017	0.205	0.001	0.020	0.321	0.183	0.004	0.026	0.892
	Sainsbury	0.476	0.006	0.889	0.429	0.002	0.148	0.625	0.027	0.001	0.000	0.418
	Tesco	0.354	0.090	0.004	0.333	0.001	0.194	0.426	0.117	0.001	0.038	0.364
	Tesco Ireland	0.377	0.124	0.030	0.365	0.002	0.182	0.533	0.146	0.002	0.023	0.469
	Waitrose	0.132	0.456	0.001	0.105	0.002	-0.105	0.365	0.560	0.007	0.034	0.998
Drinks	All Retailers	-0.229	0.113	0.014	-0.237	0.001	-0.294	-0.124	0.106	0.001	0.040	0.395
	Morissons	-0.575	0.136	0.000	-0.537	0.001	-0.635	-0.443	0.172	0.001	0.091	0.272
	Ocado	-0.291	0.359	0.000	-0.319	0.002	-0.557	-0.081	0.319	0.003	0.060	0.803
	Sainsbury	-0.294	0.442	0.000	-0.289	<0.001	-0.294	-0.269	0.451	<0.001	0.442	0.484
	Tesco	-0.643	0.139	0.000	-0.624	0.001	-0.643	-0.429	0.154	0.001	0.139	0.354
	Tesco Ireland	0.657	0.175	0.000	0.643	0.002	0.143	0.829	0.193	0.002	0.058	0.803
	Waitrose	-0.126	0.788	0.000	-0.083	0.002	-0.126	0.144	0.856	0.003	0.758	0.969
Foods and Drinks	All Retailers	0.160	0.014	0.880	0.147	0.001	0.081	0.199	0.029	0.001	0.002	0.215
	Morissons	0.077	0.649	0.000	0.076	0.001	-0.049	0.192	0.658	0.005	0.255	0.999
	Ocado	0.124	0.347	0.000	0.112	0.001	-0.046	0.199	0.407	0.005	0.128	0.991
	Sainsbury	0.160	0.317	0.000	0.127	0.001	-0.075	0.256	0.442	0.005	0.106	1.000
	Tesco	0.185	0.320	0.000	0.173	0.001	0.092	0.238	0.356	0.002	0.197	0.624
	Tesco Ireland	0.444	0.030	0.784	0.431	0.001	0.301	0.568	0.039	0.001	0.004	0.152
	Waitrose	0.018	0.911	0.000	0.002	0.002	-0.195	0.188	0.766	0.005	0.222	1.000

Dataset S1 (separate file). Most common ingredients sorted into each of the environmental database food categories.

Dataset S2 (separate file). Search terms used to sort ingredients into the environmental database food categories.

Dataset S3 (separate file). Data used to create the figures in the manuscript.

SI References

1. Harrington RA, Adhikari V, Rayner M, Scarborough P (2019) Nutrient composition databases in the age of big data : foodDB , a database infrastructure. *BMJ Open* 9(e026652). doi:10.1136/bmjopen-2018-026652.
2. Poore J, Nemecek T (2018) Reducing food's environmental impacts through producers and consumers. *Science (80-)* 360:987–992.
3. HESTIA: Food Sustanability Analytics Available at: www.hestia.earth.
4. Gephart JA, et al. (2021) Environmental performance of blue foods. *Nature* 597(August):360–366.
5. Smith MR, Micha R, Golden CD, Mozaffarian D (2016) Global Expanded Nutrient Supply (GENuS) Model : A New Method for Estimating the Global Dietary Supply of Nutrients. *PLoS One* 11(1):e0146976.
6. Standardization) I (International O for *Life cycle assessment principles and framework 14040.* (Geneva, Switzerland) Available at: www.iso.org.
7. FAO (2020) *The State of the World Fisheries and Aquaculture 2020. Sustainability in Action* (Rome, Italy).
8. Boulay AM, et al. (2018) The WULCA consensus characterization model for water scarcity footprints: assessing impacts of water consumption based on available water remaining (AWARE). *Int J Life Cycle Assess* 23(2):368–378.
9. Vitousek PM, et al. (2009) Nutrient Imbalances in Agricultural Development. *Science (80-)* 324:1519–20.
10. Röös E, Karlsson H, Witthöft C, Sundberg C (2015) Evaluating the sustainability of diets – combining environmental and nutritional aspects. *Environ Sci Policy* 47:157–166.
11. Santé publique France (2019) *Usage Regulation for the "Nutri-Score" Logo*.
12. Julia C, Etilé F, Hercberg S (2018) Front-of-pack Nutri-Score labelling in France: an evidence- based policy. *Lancet Public Heal* 3:1730474.
13. Jacobs A (2019) Consommer plus sainement: le "Nutri-Score" débarque officiellement en Belgique. *RTBF Info*.
14. Sota I (2018) Cómo funciona NutriScore, el nueve etiquetado de alimentos: críticas y virtudes del semáforo nutricional. *El Pais*. Available at: https://elpais.com/elpais/2018/11/13/buenavida/1542132354_229696.html.
15. Best D (2019) Dutch government announces support for Nutri-Score. *Just Food*.
16. Morrison O (2019) Germany plans to introduce Nutriscore: "This is a milestone in nutrition policy." *FoodNavigator*.
17. Santi P (2018) Le logo nutritionnel arrive dans les rayons des supermarchés. *Le Monde*.

18. Le gouvernement lance officiellement son étiquetage nutritionnel coloré (2017) *Ouest-France*.
19. Julia C, Hercberg S (2017) Nutri-Score : Evidence of the effectiveness of the French front-of-pack nutrition label. *Ernahrungs Umschau* 64(12):181–187.
